# Cells and Cytokines in Lung Inflammation

**DOI:** 10.1155/S0962935194000116

**Published:** 1994


Mediators of Inflammation 3, 61–99 (1994)

### Cells and Cytokines in Lung Inflammation

### Role of $TH_2$ cells in the genesis of atopic asthma and mechanisms involved in their development

*S. Romagnani*
Division of Clinical Immunology and Allergy, University of Florence, Italy.

The cellular and cytokine network that regulates the human IgE synthesis has recently been clarified. In contrast to intracellular bacteria, that induce the activation of helper T cells producing IFN-γ, but not IL-4 ($TH_1$ cells), allergens and helminths preferentially activate helper T cells that produce IL-4, but not IFN-γ ($TH_2$ cells). $TH_2$ cells provide the B cells with both signals (T/B cell contact and IL-4) required for the IgE isotype switching. Furthermore, $TH_2$ cells produce IL-5, which is a selective maturation and activation factor for eosinophils, and therefore play a key role in the triggering of the allergic cascade.

The mechanisms involved in the differentiation of helper T cells specific for bacterial constituents into the $TH_1$, and of allergen-specific helper T cells into the $TH_2$, phenotype of cytokine production have also been investigated. The presence of high concentrations of IFN-γ and the absence of IL-4 at the recognition triad (APC-Ag-TH cell) level favour the development of $TH_1$ cells, whereas the presence of IL-4 with no, or low concentrations of IFN-γ, favours the $TH_2$ differentiation. IL-12 and IFN-α production by APC and IFN-γ, produced by NK cells are probably responsible for the microenvironmental conditions favouring the $TH_1$ response, whereas early production of IL-1 by APC and of IL-4 by cells belonging to the mast cell/basophil lineage may be involved in the induction of the $TH_2$ response. Thus, the cytokine profile of the 'natural' immune response probably determines the $TH_1$ or $TH_2$ profile of the subsequent specific immune response.

These findings may provide a means for new therapeutic interventions in patients with IgE-mediated disorders aimed to inhibit the development of allergen-specific $TH_2$ responses.

### The human mast cell as a source of proinflammatory cytokines

*M. K. Church*
Immunopharmacology Group, Southampton General Hospital, Southampton S09 4XY, UK

The mast cell has long been recognized as the initiating cell of the early allergic response, histamine, prostaglandin $D_2$ and leukotriene $C_4$ giving rise to the symptoms characteristic of the immediate phase of the response. It would seem logical if the mast cell were also able to secrete cytokines capable of initiating allergic inflammation.

Studies with rodent mast cells and mast cell lines have shown messages for IL-3, IL-4, IL-5 and TNF-α to increase on cell activation. We have used immunocytochemistry, ELISA, *in situ* hybridization and the polymerase chain reaction (PCR) to explore the association of cytokines with human mast cells. Examination of bronchial biopsies from asthmatic and normal subjects and of nasal biopsies from normal and rhinitic subjects have shown the presence of immunoreactive IL-4, IL-5, IL-6 and TNF-α in mast cells with IL-4 and TNF-α in particular being up-regulated in both allergic conditions. Using immunogold, IL-4 has been localized to the mast cell granule. Using ELISA and cell culture assays we have preliminary evidence of cytokine release from purified human mast cell preparations and of IL-4 degradation, probably by chymase. *In situ* hybridization has provided evidence of IL-4 message whereas PCR has shown consistent message for IL-4 and TNF-α in stimulated mast cells.

These studies suggest that the mast cell is a source of proinflammatory cytokines capable of initiating allergic inflammation.

### Molecular control of human B lymphocyte growth and differentiation

*J. Banchereau, D. Blanchard, F. Brière, A.-C. Fluckiger, P. Garrone, S. Lebecque and F. Rousset*
Schering-Plough, Laboratory for Immunological Research, 27 chemin des Peupliers, BP 11, 69571, Dardilly, France

B lymphocytes express at their surface the CD40 antigen which belongs to the NGF receptor superfamily. The crosslinking of the CD40 antigen using a mouse fibroblastic cell line expressing the human Fc receptor (FcγRII/CDw32) and anti-CD40 monoclonal antibody induces resting B lymphocytes to enter a state of sustained proliferation. Addition of IL-4 or IL-13 results in the proliferation of human B cells and in the secretion of IgE following isotype switching. Addition of IL-10 permits limited cell proliferation but most importantly results in very high immunoglobulin production following differentiation of B cells into plasma cells. In response to IL-10, unseparated B cells cultured in the CD40 system produce $IgG_1$, $IgG_2$ and $IgG_3$ in ratio comparable to those observed in the serum. IL-10 induces naive B cells to secrete low but reproducible amounts of IgG and IgA. The combination of IL-10 and TGFβ induces naive B cells to secrete $IgA_1$ and $IgA_2$ as a consequence of isotype switching. The extracellular domain of CD40 binds with high affinity and

© 1994 Rapid Communications of Oxford Ltd high specificity to a ligand transiently expressed on activated T cells. This interaction of the CD40 antigen on B cells with its counterstructure on T cells represents a key step in T cell dependent B cell activation.

### Modulation by rminterferonγ (rmIFNγ) and CD4+ T-lymphocytes of allergic eosinophil accumulation in the mice peritoneal cavity

*C. Zuany-Amorim, D. Leduc, B. B. Vargaftig and M. Pretolani*
Unité de Pharmacologie Cellulaire/Unité Associée
Institut Pasteur-INSERM n° 285, 25, rue du Dr. Roux, 75015 Paris, France

Balb/C mice immunized to ovalbumin developed a neutrophilia and a marked eosinophilia in the peritoneal cavity 6 and 24 h, respectively, after antigen challenge (1 g, intraperitoneally, i.p.). The subcutaneous (s.c.) pretreatment with rmIFN (5 000–10 000 U/mouse) 1 h before and 6 h after antigen challenge inhibited the eosinophil infiltration by 56 and 61%, respectively. Under those conditions, the possible contamination by LPS was ruled out, since its s.c. administration failed to affect the intensity of antigen-induced eosinophilia. The s.c. treatment of sensitized mice with a monoclonal antibody anti-IFN (XMG 1.2, 1 mg/mouse) 1 h before and 6 h after ovalbumin provocation potentiated the eosinophil response. These results suggest that endogenous IFN is involved in the down-regulation of antigen-induced eosinophilia. Interestingly, rmIFN did not modify the neutrophil infiltration observed 6 h after ovalbumin provocation. In separate experiments, the s.c. treatment of sensitized animals with the immunosuppressive agent FK-506 (1.0–2.0 mg/kg) administered 1 h before and 6 h after antigen challenge reduced significantly the number of eosinophils by 54 and 60%, respectively. Finally, pretreatment of sensitized mice with a specific antibody directed against CD4+ T-lymphocytes (GKI.5, 500 g/mouse; i.v., three times), which depleted by 93% the proportion of CD4+ cells without affecting that of CD8+ cells, reduced by 53% the eosinophil infiltration in the mice peritoneal cavity induced by antigen challenge. Taken together, our results demonstrate that the eosinophil accumulation in the mice peritoneal cavity is modulated by endogenous release of IFN and by the production of substances (probably cytokines) originating from CD4+ T-lymphocytes.

We thank Dr G. Bordenave and Miss C. Denoyelle for help in the fluorescent analysis of lymphocytes and CD4+ cells exhaustion and CNPq for the fellowship of C. Zuany-Amorim.

### Effect of $\beta_2$-adrenoceptor agonists on IgE and cytokine production

*O. Coqueret, V. Lagente, C. Petit Frère, J. M. Mencia-Huerta and P. Braquet*
Institut Henri Beaufour, 1 avenue des Tropiques, 91952 Les Ulis, France

The present study examined the regulatory effect of $\beta_2$-adrenoceptor agonists on IgE and cytokine production in human and mouse. In human, salbutamol and fenoterol potentiated the *in vitro* IL-4-induced IgE synthesis by peripheral blood mononuclear cells (PBMC). These drugs inhibited IFN-γ production by phytohaemagglutinin-activated PBMC suggesting that the blockade of the production of this cytokine was involved in the enhancement of IgE synthesis. Salbutamol and fenoterol potentiated the IL-4 induced production of the soluble form of the low-affinity receptor for IgE (sCD23) although no effect on CD23 expression was observed.

In the mouse, salbutamol potentiated the *in vitro* IL-4-induced IgE synthesis from LPS-activated murine B lymphocytes. This effect was dose-dependent and maximal at 10 M and no IgE synthesis was observed when this drug was added in the absence of IL-4. When Balb/C mice are sensitized with ovalbumin (OA) and treated with daily injection of salbutamol (1 mg/kg), an increase of the anti-OA IgE levels in the serum as compared to the effect of OA alone was observed. No effect of salbutamol on plasma IgE was observed in non-sensitized mice. To investigate the *in vivo* effect of salbutamol on the release of cytokines, splenocytes obtained from treated and untreated mice were incubated for 24 h with concanavalin A (Con-A, 4 g/ml). When sensitized mice are treated with salbutamol, a significant increase of the *ex vivo* production of IL-4, IL-5, IL-6 and IL-10 was noted as compared to that obtained with splenocytes obtained from untreated sensitized mice. No effect on IL-2 and IFN- synthesis was observed. When non-sensitized mice were treated with salbutamol, a significant increase in the release of IFN-, IL-4, IL-5, IL-6 and IL-10 was noticed as compared to control mice.

These results demonstrate that $\beta_2$-adrenoceptor agonist stimulation results in an increase in IgE production both in human and mouse. In the latter species, this effect that was also observed *in vivo* could be explained by an alteration of the production of lymphokines from THl and TH2 type lymphocytes. Further studies are required to determine whether $\beta_2$-agonists exhibit similar effects *in vivo* in humans.

### Interleukin-4 (IL-4) is both released and inactivated by human mast cells

*J. M. Tunon de Lara,[1] Y. Okayama,[1] A. R. McEuen,[1] C. H. Heusser,[2] M. K. Church[1] and A. F. Walls[1]*

[1]Immunopharmacology group, Southampton General Hospital, S09 4XY; [2]Ciba Geigy, Basel, Switzerland

It has recently been suggested that mast cells are an important source of inflammatory cytokines in allergic disease. Rodent mast cells have been shown to produce and release IL-4 and it has recently been demonstrated that human mast cells contain IL-4. On activation, mast cells also release substantial quantities of proteases including tryptase, chymase, cathepsin G and carboxypeptidase, which could help to control the bioavaibility of cytokines. We have investigated the potential of mast cells to release IL-4 and have determined the extent to which this cytokine may be degraded by mast cell proteases.

Mast cells were dispersed from foreskin or lung tissue, using collagenase and hyaluronidase. Skin mast cells were purified using a discontinuous Percoll gradient (mast cell purity >85%) and lung mast cells using an immunomagnetic procedure with anti-c-kit monoclonal antibody (YB5B8) coupled to Dynabeads (mast cell purity >75%). Purified mast cells were challenged with anti-IgE (10%) and IL-4 was measured in the supernatants by a specific ELISA, and histamine using a spectrofluorometric procedure. Lysates of purified mast cells were incubated with human recombinant IL-4 at 37°C and the IL-4 concentrations were measured by ELISA or by bioassay with an IL-4-dependent cell line (C-rh4S).

After an anti-IgE activation inducing 17 ± 6% of histamine release, 6.4 ± 3.6 ng of IL-4 were measured in the supernatants from $10^6$ lung mast cells, but there was no detectable IL-4 in that from skin mast cells. A dose-dependent decrease in IL-4 concentration following addition of mast cell lysates was observed by both ELISA and bioassay. The rate of IL-4 catalysis by the skin mast cell lysates was significantly higher than that for the lung mast cell lysates (20 pg and 6 pg of IL-4 degraded/min per $10^4$ cells, respectively) and was related to the chymotryptic activity as measured with the chromogenic substrate Suc-Ala-Ala-Pro-Phe-NA. The degradation of IL-4 was inhibited by the protease inhibitors aprotonin, chymostatin and SBTI, but not by TLCK, TPCK, or $\beta_2$-macroglobulin.

These data indicate that a chymotryptic mast cell protease can degrade IL-4 and thus, may control the biological actions of IL-4 following its release from the mast cell itself.

### Induction of differentiation of human mast cells from bone marrow and cord blood progenitor cells by factors produced by a mouse stromal cell line

*M. Arock,*[1] *F. Hervatin,*[2] *J. J. Guillosson*[1] *and D. Thierry*[2]
[1]Laboratoire d'Hématologie, Faculté de Pharmacie, 4, Ave de l'Observatoire, 75006 Paris; [2]L.I.R.B., Hôpital Saint-Louis, 1, Ave C. Vellefaux, 75475 Paris Cedex 10, France

Human bone marrow or cord blood progenitors (i.e CD34+ cells are now easily purified by immunological methods involving, immunomagnetic beads. These CD34+ cells can be cultivated on normal human bone marrow stromal cells for limited periods. In this condition, the number of the surviving progenitors declines in a few weeks and these cells disappear completely in less than 8 weeks, a fact suggesting that this culture system is deprived of growth factor(s) able to support the self-renewal of stem cells. We have developed in our laboratory the culture of immunomagnetically purified bone marrow or cord blood derived CD34+ cells on a supportive mouse stromal cell line named MS-5. The long term survival of clonogenic cells was analysed in these cultures and compared with results obtained by culture on human bone marrow stromal cells. Results demonstrated that only coculture of CD34+ cells on MS-5 layers allows the survival of clonogenic progenitors for at least 12 weeks. Cytospin smears were regularly made during, the culture period and cell morphology was examined after classical staining, methods (i.e M.G.G. and toluidine blue staining). This analysis revealed the growth of metachromatic cells resembling, mast cells from the second week of incubation on the MS-5 layer. The highest percentage of these cells was observed at 8 weeks and reached at least 30% for cord blood cells and 70% for bone marrow cells. To further confirm the nature of these metachromatic cells, immunohistochemical staining of tryptase was performed on the same samples and similar percentages of tryptase+ cells were observed. In the view to determine whether the contact between MS-5 layers and CD34+ cells was an absolute requirement for the appearance of mast cells, CD34+ cells were cultured in the presence of MS-5 conditioned medium. This condition allowed the same development of mast cells when compared with the coculture experiments. Whatever the soluble factor(s) responsible for this mast cell growth activity, our culture system allows us to obtain significant amounts of highly enriched normal human mast cells populations useful for further studies on the reactivity of this cell subset.

### Cytokines and airway inflammation

*P. Howarth*
Department of University Medicine, University of Southampton, UK

Cytokines released from activated cells within the bronchial and nasal mucosa orchestrate the pattern of allergic tissue inflammation in asthma and rhinitis. On account of their profile of actions, emphasis has focused on the role of IL-3, IL-4, IL-5, TNFα and GMCSF. Between them these cytokines promote progenitor cell development and growth, adhesion molecule expression on the vascular endothelium, cell chemotaxis and cell activation.

Gene expression studies, either through RNA extraction and PCR amplification or by *in situ* hybridization identify upregulation of IL-4 and IL-5 in asthma and rhinitis. Immunohistochemical staining of tissue biopsies reveals IL-4, IL-5, TNFα and GMCSF product in the preformed state IL-4, IL-5 and TNFα are localized to mast cells and GMCSF to the airway epithelium. No cytokine product can be localized to T lymphocytes although T lymphocytes, recovered by bronchoalveolar lavage have upregulated gene expression for IL3, IL-4, IL-5 and GMCSF in asthma and elevated levels of IL-4, IL-5 and GMCSF along with IL-1 and IL-6 have been reported in bronchoalveolar lavage in allergic asthma.

A model is thus developing of the mast cell as the acute responder within the airways, having preformed cytokines stored within its cytoplasmic granules, and following immunological insult initiating the inflammatory process. The release of IL-4 from this cell population acts as a stimulator for activation of the CD4+ T lymphocytes. This subsequent T lymphocyte activation, with enhanced gene expression and product production and release (but not storage), contributes to the chronicity of the response by maintaining the airway inflammation. This chronicity will also be promoted by epithelial production of GMCSF, as this cytokine prolongs tissue persistence of eosinophils.

### Growth- and colony-stimulating factors mediate eosinophil/fibroblasts interactions in chronic airway inflammation*

*J. Gauldie, M. Jordana, I. Ohno, G. Cox and H. Kirpalani*
Departments of Pathology, Medicine and Pediatrics, McMaster University, Hamilton, Ontario, Canada L8N 3Z5

In many states of chronic inflammation of the tissues of the airways, such as seen in asthma, chronic rhinitis, nasal polyposis and to some extent pulmonary fibrosis, there is a notable accumulation of eosinophilic granulocytes which have been implicated in the reactivity of the tissue and which likely contribute to tissue destruction and remodelling. We are now aware that the cells of the tissue, including fibroblasts, endothelial cells, epithelial cells and smooth muscle cells are able to release a number of growth and differentiating factors that have a profound influence on the accumulation and function of granulocytes in the tissue. Thus fibroblasts are stimulated by cytokines such as IL-1 and TNF to release the colony stimulating factors (M-, G and GM-CSF), factors affecting eosinophil behaviour, as well as TGFβ and other factors which are chemotactic for granulocytes. In turn, eosinophils in the tissue are known to release TNF, TGFβ and PDGF, factors which will impact on the behaviour and differentiation of the tissue cells. We will present details of the characterization of these cell and cytokine networks with examples of both paracrine and autocrine ftmction in the cellular communication between the tissue cells and granulocytes. Such interactions are undoubtedly crucial in the initiation of tissue remodelling and in the chronic nature of the inflammatory response of the tissue.

*Supported by MRC Canada and Astra Sweden.

### Rantes, a novel eosinophil chemotactic cytokine

*J.-M. Schröder, Y. Kameyoshi and E. Christophers*
Department of Dermatology, University of Kiel, Schittenhelmstr. 7, 2300 Kiel, Germany

In a number of allergic diseases such as asthma eosinophils (Eo) are known to be prominent effector cells. Reasons for the appearance of this leukocyte type are yet speculative, and could come from the local generation of Eo-attractants such as PAF, LTB4, C5a or IL-5 and GMCSF.

Recently a novel family of leukocyte-selective cytokines termed 'chemokines' has been detected. Members of this family are neutrophil-selective (IL-8, Gro, NAP-2) or monocyte-selective (MCP-1) chemotaxins. We addressed the question whether also eosinophil-chemotactic chemokines do exist. In order to test this working hypothesis we stimulated human peripheral blood leukocyte preparations with mitogen together with bacterial lipopolysaccharide and phorbolester and analysed supernatants after RP-HPLC for Eo-chemotactic proteins, which we detected. It became clear that these Eo-chemotactic cytokines at least in part originated from contaminating platelets. Therefore platelets were stimulated with thrombin and Eo-chemotactic activity present in supernatants was purified to homogeneeity by the use of HPLC-techniques. $NH_2$-terminal sequence analyses revealed two polypeptides showing a sequence, which is identical with that of the cytokine Rantes, which is a member of the 'C-C'-branch of the chemokine family.

Rantes appeared to be a potent attractant for human eosinophils ($ED_{50}$: 4 nM), which did not show any chemotactic properties for neutrophils. Moreover cross-desensitization studies revealed that Rantes appears to bind to a receptor distinct from those known for other Eo-attractants. We conclude that Rantes could be an important cytokine in platelet-associated Eo-accumulation seen in some inflammatory conditions.

### Chronic treatment with the interleukin-1 receptor antagonist (IL-1ra) inhibits pulmonary hypertension in monocrotaline (MCT) treated rats

*N. F. Voelkel, J. Bridges, M. A. Voelkel, W. Franklin and W. Arend*
Pulmonary and Rheumatology Divisions, Dept of Pathology, UCHSC, 4200 E. 9th Ave., Denver, Colorado, 80262, USA

Cytokines are released together with lipid mediators in the lungs of rats during the inflammatory response following the single s.c. injection of monocrotaline (an alkaloid that produces pulmonary hypertension in rats). Lung levels of platelet activating factor (PAF) are measurably increased after MCT and treatment with a PAF-antagonist prevents the MCT-induced pulmonary hypertension and lung vascular remodelling.[1] Since PAF can lead to the formation of IL-1, we measured IL-1 in lung tissue following MCT injection and injected 2 mg/kg IL-1ra i.p. b.i.d. into MCT-and into sham (saline) injected Sprague–Dawley rats. We hypothesized that MCT would cause an increase in lung IL-1 and further that IL-1ra treatment would inhibit the development of MCT-induced pulmonary hypertension. MCT increased lung IL-1 levels (measured by ELISA, using a rat-specific IL-1 antibody), but less than was observed after i.p. endotoxin priming (E). In lung homogenates from saline injected rats IL-1 was undetectable. MCT also caused increased gene expression of IL-1 and IL-1ra when compared with control animals. Using a rat-specific monoclonal antibody against IL-1 we found that MCT caused specific binding of the antibody to macrophages and to lung vessels. Chronic IL-1ra treatment inhibited the MCT-induced pulmonary hypertension. We concluded that IL-1 participates in the development of pulmonary hypertension in this model of chronic pulmonary inflammation.

Supported by a Grant in AID of the American Heart Association. IL-1ra was kindly provided by Synergen Boulder, Colorado.

### References

1. *J Appl Physiol* 1991; **71**: 2492.

### Analysis of cytokine transcripts in the bronchoalveolar lavage cells of patients with asthma

*S.-K. Huang, G. Krishnaswamy, S.-N. Su, H.-Q. Xiao and M. C. Liu*
Johns Hopkins University School of Medicine, Johns Hopkins Asthma and Allergy Center, Baltimore, ND 21224, USA

Cytokines have been shown to play a critical role in the pathogenesis of atopic asthma. We have analysed a panel of steady-state cytokine mRNAs in the bronchoalveolar lavage (BAL) cells from allergic asthmatic subjects or patients challenged with ragweed allergen. This was achieved by combining both qualitative and quantitative assays using the reverse transcription polymerase chain reaction (RT-PCR). Analysis of BAL cells from six mild allergic asthmatic and five non-asthmatic, non-allergic subjects showed no qualitative differences in the profile of cytokine mRNAs (including IL-5, and IFN-γ), except for TNF-α which was detected in three out of six asthmatic BAL samples, but none of the controls.

A key cytokine, IL-5, has been implicated in the pathogenesis of allergic inflammation through the recruitment of eosinophils. We found a significant enhancement of steady-state IL-5 transcripts in the BAL cells from ragweed allergen-challenged as compared with the saline-challenged control sites of four patients; furthermore, the cellular source for IL-5 mRNA was identified in the mononuclear-cell fraction, but not in the purified eosinophils, of the allergen-challenged BALS. These results suggest that the significant increase of IL-5 transcripts is primarily from the infiltrating mononuclear cells.

### The three dimensional structures of interleukin-5 at 2.4 Å resolution, implications for the structures of other interleukins

*M. V. Milburn,[1] A. M. Hassel,[1] M. H. Lambert,[1] S. R. Jordan,[1] C. Y. Arod,[2] A. E. I. Proudfoot,[2] P. Graber[2] and T. N. C. Wells[2]*
[1]Glaxo Research Institute, Department of Structural and Biophysical Chemistry, 5 Moore Drive, Research Triangle Park, N.C. 27709, USA; [2]Glaxo Institute for Molecular Biology, 14 chemin des Aulx, 1228 Plan-les-Ouates, Geneva, Switzerland

Interleukin-5 (IL-5) is a lineage-specific cytokine for eosinophilpoesis and plays an important role in diseases that involve elevated levels of eosinophils, such as asthma. Human IL-5 is a disulphide-linked homodimer of 115 amino acid residues in each chain. The crystal structure at 2.4 Å resolution reveals a novel two domain structure with each domain showing a striking similarity to the cytokine fold exhibited by granulocyte–macrophage colony stimulating factor (GM-CSF), macrophage colony stimulating factor (M-CSF), interleukin-2 (IL-2), interleukin-4 (IL-4), and human and porcine growth hormone (hGH and pGH). IL-5 is unique in that each domain requires the participation of two chains. The core of the IL-5 structure consists of two left-handed bundles of four helices laid end to end and two short β-sheets on opposite sides of the molecule. Surprisingly, the C-terminal strand and helix of one chain complete a bundle of four helices and a β-sheet with the N-terminal three helices and one strand of the other chain. The structure of IL-5 provides a molecular basis for the design of antagonists and agonists that would delineate receptor recognition determinants critical in signal transduction. This structure determination extends the family of the cytokine bundle of four helices and emphasizes its fundamental significance and versatility in recognizing its receptor.

### Identification of interleukin-4 as the major *in vivo* determinant of helper T cell IL-5 production (TH2 phenotype commitment) by direct gene targeting in mice

*G. P. Anderson,[1] B. Hengstier,[1] G. LeGros,[1] M. Kopf[2] and M. A. Bray[1]*

[1]Ciba-Geigy AG, Basel, Switzerland; [2]Max Planck Institut für Immunologie, Freiburg, Germany

Eosinophilic inflammation is driven by IL-5 produced predominantly by helper CD4+ T cells of the TH2 phenotype (IL-4, IL-5 but little IFNγ) but the determinants of CD4+ commitment to TH2 IL-5 production are unknown. IL-4 is essential to induce T cell IL-4 synthesis *in vitro* suggesting a role in TH2 commitment.[1] To study this we have used embryonic gene targeting to delete the genomic IL-4 gene in mice (C57BL6 × 129cv) *in vivo*. IL-4 gene deleted (0/0) and wild type control (+/+) mice were infected with 750 stage 3 larvae of the helminth *Nippostrongylus brasiliensis* by s.c injection. *N. brasiliensis* infection causes an intense T cell- and IL-5-dependent pulmonary and systemic eosinophilia and IL-4-dependent elevated IgE . Differential counts of peripheral blood made over 13 days showed attenuated eosinophilia in gene targeted (0/0) mice (+/+, 6.0E3; 0/0, 2.3E3 eos/ml, day 13, $p < 0.05$, *t*-test). Marked differences were observed in BAL eosinophils (+/+,1.8E6; 0/0, 4.0E5, 1.2 ml lavage, day 13, $p < 0.05$) and fewer eosinophils were observed histologically in lung inflammatory cell infiltrates in (0/0) mice. Serum IgE was undetectable in (0/0) mice. Lung cytokine mRNA levels (quantitative PCR, transcripts/μg RNA) measured on day 7, before the onset of peak eosinophilia, showed reduced IL-5 and IL-6 in gene targeted mice. mRNA Levels of IL-2, IL-1α, and IFNγ were not changed. Cytokine protein determined in (0/0) mice by bioassay or ELISA from supernatants of CD4 or CD8 lymphoid T cells restimulated *ex vivo* with anti-CD3 showed weak CD4+ IL-5 production and induction of CD8+ T cells weakly producing IL-5.

| mouse | IL-4 | IL-5 | IL-6 | IL-2 |
|---|---|---|---|---|
| +/+ | 50 | 150 | 2 000 | 1.6 |
| 0/0 | 0 | 20 | 400 | 1 |

Neutralizing antibodies to IL-5 prevented and antibodies to IFNγ did not augment eosinophilia. These data suggest that (a) IL-4 is a major, but replacable, determinant of T cell commitment to TH2 phenotype *in vivo*. (b) transmigration of eosinophils into the lung can occur in the absence of IL-4 and IgE (c) the identification of IL-5 producing CD8+ cells indicates that eosinophilia may not be strictly governed by CD4+ TH2 cells and has implications for the pathogenesis and therapy of eosinophil mediated diseases.

**Reference**
1. LeGros *et al. J Exp Med* 1990; 172: 921-929.

### Effects of anti-adhesion antibodies in primate models of acute and chronic lung inflammation

*L. G. Letts, R. H. Gundel, L. Churchill and C. D. Wegner*
Boehringer Ingelheim Pharmaceuticals Inc., 900 Ridgebury Road, Ridgefield, CT 06877 USA

We have developed several primate models of lung inflammation in which cell infiltration into the airways appears associated with the onset and progression of altered lung function. In each of these models the role of cell migration and their activation has been examined using specific monoclonal antibodies to adhesion proteins. The 'acute' model involves a single exposure to inhaled antigen whereas the 'induced' model involves three exposures on alternate days. The results on several variables are shown in the table:

| | Acute | | Induced | |
|---|---|---|---|---|
| mAb | LPR | PMN | AHR | Eos. |
| ICAM-1 | - | - | +++ | +++ |
| CD11b | NT | NT | +++ | - |
| E-Selectin | +++ | +++ | - | - |
| VCAM-1 | NT | NT | - | - |

(LPR, Late phase response; AHR, airway hyperresponsiveness; NT, not tested).

In addition BAL fluid, lung tissue histology and immunohistochemistry was used to monitor cell activation, cytokine/mediator production and adhesion protein expression. The 'acute' model involves an early and late phase airway obstruction, whose intensity and time to peak correlates with a PMN influx. The 'induced' model involves a predominant eosinophil influx associated with increases in airway responsiveness. Collectively, the data emphasize the importance of the eosinophil and its mediators in lung inflammation. In addition, discussion will involve recent studies identifying T cell subsets and their potential role in the initiation of the early events in these models. Also, the effects of mAbs on a murine model of RSV-induced lung inflammation will be presented.

### Interactions between respiratory epithelial cells and cytokines: relationships to lung inflammation

*K. B. Adler,[1] L. A. Cohn[1] and S. Becker[2]*

[1]North Carolina State University, Raleigh, NC; [2]TRC Environmental Corp., Chapel Hill, NC, USA

Epithelial cells lining respiratory airways can participate in inflammation in a number of ways. They can act as target cells, responding to exposure to a variety of inflammatory mediators and cytokines by altering one or several of their functions, such as mucin secretion, ion transport, or ciliary beating. Aberrations in any of these functions can affect local inflammatory responses and compromise pulmonary defence. For example, oxidant stress can increase secretion of mucin, and depress ciliary beating efficiency, thereby affecting the ability of the mucociliary system to clear potentially pathogenic microbial agents. Recent studies have indicated that airway epithelial cells also can act as 'effector' cells, synthesizing and releasing cytokines, lipid mediators and reactive oxygen species in response to a number of patholologically-relevant stimuli, thereby contributing to inflammation. Many of these epithelial-derived substances can act locally, affecting both neighbouring cells and tissues, or, via autocrine or paracrine mechanisms, affect structure and function of the epithelial cells themselves. Studies in our laboratories utilized cell cultures of both human and guinea-pig tracheobronchial and nasal epithelial cells, and isolated human nasal epithelial cells, to investigate activity of respiratory epithelial cells *in vitro* as sources of cytokines and inflammatory mediators. Primary cultures of guinea-pig and human tracheobronchial and nasal epithelial cells synthesize and secrete low levels of IL-6 and IL-8 constitutively. Production and release of these cytokines increases substantially after exposure to specific inflammatory stimuli, such as TNF or IL-1, and after viral infection.

Supported in part by grant HL 36982 from NIH, a grant from the state of North Carolina, and grants from Hoffmann La Roche, Inc., Nutley, NJ, and Glaxo, Inc., RTP, NC. This abstract of a proposed presentation does not necessarily represent EPA policy.

### Chemical mediators and adhesive molecules involved in eosinophil accumulation in allergic reactions *in vivo*

*T. J. Williams, V. Weg, S. Nourshargh, D. A. Griffiths-Johnson, D. T. Walsh, P. D. Collins and P. J. Jose*
Department of Applied Pharmacology, National Heart and Lung Institute, Dovehouse St, London SW3, UK

We have been investigating mechanisms underlying the accumulation of eosinophils in allergic inflammatory reactions in the guinea-pig using cells labelled with $^{111}$In.[1] In passive cutaneous anaphylactic (PCA) reactions $^{111}$In-eosinophils accumulated rapidly with a peak rate over the first 30 min, accumulation continuing for 4 h. Plasma protein leakage, measured using $^{125}$I-albumin, was dissociable from $^{111}$In-eosinophil accumulation; virtually all leakage occurring from 0 to 30 min.

To investigate the mechanisms involved in selective eosinophil, as opposed to neutrophil accumulation *in vivo*, we have used an antibody to the $\beta_1$ integrin VLA-4 (HP1/2, a gift from Dr. Roy Lobb, Biogen).[2] Pre-incubation of $^{111}$In-eosinophils with the antibody prior to i.v. injection, blocked their accumulation in PCA sites. Accumulation in response to i.d. C5a, PAF and LTB, was also blocked effectively. HP1/2 administered i.v. also blocked the accumulation of $^{111}$In-eosinophils in the skin, with no effect on plasma leakage.

None of the antagonists to known mediators blocked eosinophil accumulation in PCA reactions although plasma leakage could be blocked by combinations.[3] We have therefore set up experiments to identify eosinophil chemoattractants generated *in vivo*. Bronchoalveolar lavage fluid from sensitized guinea-pigs challenged with ovalbumin was injected into guinea-pig skin and the accumulation of $^{111}$In-eosinophils was measured. Using this technique a potent, selective eosinophil chemoattractant, 'eotaxin', has been purified. The source of this protein is under investigation.

We thank the National Asthma Campaign, The Wellcome Trust and CAPES (Brazil).

**References**

1. Faccioli LH, Nourshargh S, Moqbel R, *et al. Immunology* 1991; **73**: 222–227.
2. Weg VB, Williams TJ, Lobb RR, Nourshargh S. *J Exp Med* 1993; **177**: 561–561.
3. Weg VB, Watson ML, Cordeiro RSB, Williams TJ. *Eur J Pharmacol* 1991; **204**: 157–163.

### Transendothelial migration of eosinophils in allergic inflammation

*R. Moser[1] and P. L. B. Bruijnzeel[2]*

[1]Department of Internal Medicine/Hematology, University Hospital A-Hof 143, CH-8091 Zurich, Switzerland; [2]Department of Pharmacology, Medical Biological Laboratory, TNO, Rijswijk, The Netherlands

Beside lymphocytes, eosinophils are the most prominent cell type in allergic inflammation. Why particularly eosinophils transmigrate the endothelial barrier is still unclear. Our investigations in this phenomenon led to the following data. (A) 'Priming' of circulating human eosinophils from non-allergic donors by pM concentrations of IL-3, IL-5 and GM-CSF renders eosinophils the capacity to transmigrate IL-1-activated human umbilical vein endothelial cell (HUVEC) monolayers, whereas non-primed eosinophils do not. In contrast, eosinophils from allergic individuals (already 'primed' *in vivo*) spontaneously transmigrated the IL-1-activated endothelial barrier. Using HUVEC monolayers on microporous filters, this transmigration was severely impaired by anti-CD18 mAbs, but not by the mAb BP2/1 against VLA4. (B) Using bilayer vascular constructs (BVC), activation with IL-4 led to selective transmigration of eosinophils. Again, this transmigration was restricted to *in vitro* or *in vivo* 'primed' eosinophils and was partially blocked by mAbs against CD11/CD18 and VLA-4. IL-1-induced transmigration of eosinophils was partially the result of enhanced ICAM-1 expression. The lack of inhibition by mAb HP2/1 points against the involvement of VCAM-1, despite VCAM-1 and ELAM-1 were both presented by IL-1-activated HUVE. It remains a role for ELAM-1. In contrast, IL-4 activation was followed by selective expression of VCAM-1. No induction of ELAM-1 was detectable. Together with constitutive ICAM-1, VCAM-1 is thought to be the basis of the selective eosinophil transmigration. (C) The monolayer-on-filter system allowed non-selective passage of neutrophils and eosinophils in response to IL-1 and TNF. Whereas in the BVC IL-1 and TNF only provoked transmigration of neutrophils and not of eosinophils. These findings allow the important question: why do eosinophils fail to transmigrate IL-1-activated BVC in the presence of elevated ICAM-1 and VCAM-1 levels?

### Expression of ELAM-1, ICAM-1 and VCAM-1 on bronchial biopsies from allergic asthmatic subjects

*P. Gosset, I. Tillie-Leblond, A. Janin, C. H. Marquette, M. C. Copin, B. Wallaert and A. B. Tonnel*

CJF 90-06, Institut Pasteur and CHR, Lille, France

Eosinophil and lymphocyte infiltration has been demonstrated in the lung of asthmatic patients. The involvement of adhesion molecules in the leucocyte migration has been shown by *in vitro* experiments and in an animal model of allergic asthma. Moreover, supernatants of alveolar macrophages from allergic subjects developing a LAR amplified the *in vitro* endothelial expression of ICAM-1 and ELAM-1 by a TNF-dependent mechanism. The aim of this study was to evaluate in patients with allergic asthma, the ELAM-1, ICAM-1 and VCAM-1 expression on pulmonary endothelium and bronchial epithelium. Thirteen patients with allergic asthma and ten control subjects were biopsied under fibreoptic bronchoscopy. After paraffin embedding, cellular infiltrate was evaluated by May–Grünwald–Giemsa staining and adhesion molecule expression by immune-histochemistry study using mouse monoclonal antibodies (British Biotechnology, Oxford, UK). The results were expressed as the percentage of positive cells. Before the bronchoscopy, we evaluated each patient for asthma severity by the score of Aas, lung function and treatment. Local accumulation of eosinophils and lymphocytes was detected in eleven asthmatic subjects whereas none was observed in control subjects. Using immunohistochemistry, a low expression of ICAM-1 was revealed on biopsies from control subjects. ICAM-1 expression on epithelial and endothelial cells and ELAM-1 expression on endothelial cells were significantly increased compared with the control subjects ($p < 0.05$ in all cases). A significant correlation was obtained between the mucosal eosinophilia and ICAM-1 ($r = 0.66$, $p < 0.01$) and ELAM-1 expression on endothelium ($r = 0.71$, $p < 0.02$) whereas no relation was shown between the adhesion molecule expression and the score of Aas. No change in cellular infiltration or in adhesion molecule expression was observed in patients treated by inhaled corticotherapy compared to untreated patients. In conclusion, an increase of adhesion molecule expression on epithelial and endothelial cells was observed in allergic asthmatics compared to control subjects. The possible involvement of TNF in this process is under investigation.

### Endotoxin-induced neutrophil adherence to endothelium: relationship to CD11b/CD18 and L-selectin expression and matrix disruption

*A. Finn, S. Strobel, M. Levin and N. Klein*
Division of Cell and Molecular Biology, Institute of Child Health, 30 Guilford St., London WCLN 1EH and Department of Paediatrics, St. Mary's Hospital Medical School, South Wharf Rd., London, W2 1NY, UK

Vascular endothelial injury observed in severe bacterial infection may be caused by neutrophil-derived enzymes. Adherence to the endothelium, a prerequisite for this process, is mediated sequentially by the neutrophil adhesion molecules L-selectin and the $\beta_2$ integrins including CD11b/CD18. We have explored the relationship between expression of these molecules, neutrophil adherence, endothelial activation and consequent endothelial injury, as assessed *in vitro* by changes to heparan sulphate and fibronectin matrices which colocalize. Endothelial prestimulation with lipopolysaccharide (endotoxin) caused an increase in adherence and a generalized reduction in heparan sulphate; disruption of the fibronectin matrix only occurred on the further addition of fMet-Leu-Phe. Although maximal disruption of these matrices was associated with elevation of neutrophil CD11b/CD18 and reduction in L-selectin expression, these changes did not determine either the nature or extent of endothelial damage. CD11b/CD18 expression was similar in both adherent and non-adherent neutrophils, while L-selectin was shed in association with adherence in the absence of other stimuli. These changes in expression were thus independently regulated. This model may provide further insights into the interrelationship between neutrophil adhesion and activation and endothelial damage in infection with Gram-negative bacteria.

### LFA-1 is a key mediator of eosinophil migration to the airways in a murine model of allergic airways inflammation

*C. A. Hébert,[1] P. Jardieu,[1] T. Terrell,[1] B. Lyon,[1] R. J. Holmes,[1] G. Hatami,[1] G. Brusselle[2] and R. Pauwels[2]*

Genentech, SO San Francisco CA 94080, USA; [2]Roche Research/University of Gent, 9000 Gent, Belgium

Balb/c mice were sensitized i.p. with ovalbumin/alum. Fourteen days after sensitization the mice were subjected to daily aerosolizations of 0.1% ovalbumin in PBS for 7 days. Twenty-four hours after the last aerosolization, the lungs were lavaged. Differential counts of the cells in the lavage fluid indicate that mice that were sensitized and aerosolized (*group* +) developed a profound eosinophilia (50-75% of lavage cells were eosinophils). In contrast, no eosinophils were present in total lung lavages from naive mice or from mice which received aerosol challenge without antigen sensitization or aerosol challenge only. Histological examination indicates that the eosinophil infiltration occurs at the level of the major airways and is accompanied by changes in the morphology of the bronchial epithelium which becomes hypertrophied. Alcian blue/PAS staining suggests that the airways epithelial cells of *group* + mice are actively secreting mucus and glycoproteins. Furthermore, mucus plugs can be seen in the airways of non-lavaged animals from *group* +. Additional characteristics of this model include a profound mucosal infiltrate of macrophages, lymphocytes and plasma cells and the development of an ovalbumin-specific IgE titre. Two separate experiments (six animals per group) indicate that treatment of the mice with M17, a monoclonal antibody against CD11a, (100 μg i.v. on the day of the first aerosol challenge) causes a 91% and a 92% decrease, respectively, in the number of eosinophils found in the total lung lavages. The number of eosinophils found in the lavages of mice treated with an isotype control antibody was equal to the number of eosinophils found in the mice which did not receive antibody treatment (*group* +). These results indicate that LFA-1 is a key component in the cascade of events which lead to the migration of eosinophils to the airways.

### Role of macrophages in pulmonary diseases

*A. B. Tonnel, P. Gosset, D. Vanhee and B. Wallaert*
CJF INSERM 90.06, Institut Pasteur de Lille, 59019 Lille cédex, France

The alveolar macrophage (AM) is a critically important cell playing a prominent role in lung inflammation by producing oxygen radicals, enzymes, arachidonic acid metabolites but also a large panel of cytokines. Among interstitial lung disorders, our interest was recently focused on Coal Worker's Pneumoconiosis (CWP), which is one of the most widespread fibrotic lung diseases. Although its physiopathology remains incompletely understood several lines of evidence suggest the participation of AMs at least in the initiation of the alveolitis.

In coal miners, bronchoalveolar lavage showed a large influx of mononuclear phagocytes with a spontaneous increased production of oxidants, fibronectin, neutrophil chemotactic factor, but also of interleukin-1 (IL-1), and tumour necrosis factor α (TNFα). This spontaneous cytokine release was higher in patients with progressive massive fibrosis (PMF) than in simple pneumoconiosis (SP). However, we also observed a spontaneous high cytokine release by AMs from coal miners still working and exposed to coal mine dust, indicating that TNFα may participate in the early phase of lung inflammation.

*In vitro* exposure of AMs (obtained from healthy subjects) to coal dust particles triggered a significant release of TNFα and interleukin-6, by comparison with titanium oxide used as a biologically inert control dust. Moreover, it appeared that coal mine dust (i.e. a mixture of silica and coal particles) was more aggressive than similar concentrations of pure silica, suggesting that cytokine secretion induced by coal mine dust was not exclusively related to the presence of silica but resulted from a complex interaction between the different components.

In the last part of this study we concomittently evaluated in AM supernatants from pneumoconiotic patients, two pro-fibrotic factors (PDGF and IGF-1) and the production of transforming growth factor (TGFβ): interestingly, while PDGF and IGF-1 levels were largely secreted in patients with PMF, TGFβ production was predominant in AMs obtained from patients with SP, suggesting a potential protective effect of TGFβ on the development of pulmonary fibrosis.

### The role of macrophage-derived PDGF in lung fibroblast proliferation

*A. R. Brody,*[1,2] *J. C. Bonner*[1] *and L. Blalock*[1]
[1]Laboratory of Pulmonary Pathobiology, National Institute of Environmental Health Science, N.C. 27709;
[2]Department of Pathology, Tulane University Medical Centre, 1740 Tulane Ave., New Orleans, La. 70112, USA

Lung interstitial and alveolar macrophages have been shown to synthesize and secrete a wide array of cytokines and growth factors which could mediate pulmonary inflammation and fibrosis. The most potent known inducer of mesenchymal cell proliferation is platelet-derived growth factor (PDGF). Thus, we have been studying the biology, biochemistry and molecular characteristics of macrophage-derived (MD)-PDGF *in vitro* and *in vivo* in our model of asbestos-induced lung fibrosis. Initially, we showed that macrophages from rats secrete increased amounts of PDGF *in vitro* after asbestos treatment in a concentration-dependent fashion. This dimeric protein (~30 kDa) was predominantly the BB isoform of PDGF, although all three isoforms (AA, AB, BB) were synthesized by macrophages. This was determined by an ELISA using several monoclonal antibodies against PDGF and by *in situ* hybridization of the mRNAs for both PDGF A- and B-chains in macrophages *in vitro*. The MD-PDGF proved to be an excellent mitogen for early passage rat lung fibroblasts, and cell proliferation was blocked by a polyclonal antibody to PDGF. Since we showed that brief asbestos inhalation by rats and mice caused up to 30-fold increases in epithelial, mesenchymal and vascular cell proliferation, we asked if the PDGF A- and B-chain mRNAs were expressed in the lungs of exposed animals. *In situ* hybridization revealed that both mRNAs were up-regulated in lung macrophages, epithelial and interstitial cells by 2 weeks after asbestos exposure. Unexposed animals, exhibited few labelled cells while tissues hybridized with a 'randomer' probe were completely negative. In summary, we have shown that lung macrophages synthesize and secrete all three PDGF dimers, and the BB isoform is a potent mitogen for lung fibroblasts. After asbestos inhalation, the mRNAs which code for the PDGF A- and B-chains are up-regulated in the lung at sites associated with initial fibre deposition and macrophage accumulation. Our ongoing studies are focused on the potential of PDGF as a primary mediator of mesenchymal cell proliferation in lung fibrogenesis.

### The role of alveolar macrophages in the regulation of lung inflammation

*T. Thepen,*[1] *G. Kraal*[2] *and P. G. Holt*[3]
[1]Department of Dermatology, AZU, Utrecht, Heidelberglaan, 100 3584 CX Utrecht, The Netherlands;
[2]Department of Histology, Vrije Universiteit, 1081 BT, Amsterdam, The Netherlands and [3]WARICH Princess Margaret Hospital Perth, Western Australia

Because of their strategic positioning in the lung, alveolar macrophages (AM) have been assigned an important role in lung inflammation. Their active participation in the effecter arm of pulmonary immunoinflammatory responses, via such functions as receptor-mediated phagocytosis and secretion of pro-inflammatory mediators has been recognized for many years. However, recent studies performed in our laboratories show that, after *in vivo* depletion of AM via intratracheal administration of a liposome encapsulated drug dichloromethylene diphosphanate (DMDP) experimental animals become hyper-responsive to inhaled allergens. The animals show both an increased primary as well as secondary pulmonary immune response. In the latter, the IgE response increases dramatically with the numbers of plasma cells, including IgE producing plasma cells, and T cells entering the lung. The experiments indicate that this control is excerted at the T cell level. In addition to this, we show that the regulatory function of AM is not limited to this level. The antigen presenting cell function of pulmonary dedritic cells is also down-regulated by AM thus preventing the presentation of allergen to T cells in the lung after allergen inhalation. Next to their role in preventing inflammation in lung tissue as described above, we present evidence that AM might be involved in the regulation of pulmonary immune responses in the draining lymph nodes. We show that AM are able to migrate from the alveolar space into the draining lymph nodes and in particular to the paracortical T cell area. This migration pattern is aberrant from that of other types of macrophages that migrate to subcapsuler sinus and medulla of the lymph nodes and suggests an additional immunoregulatory function of AM in the draining nodes, stressing the importance of AM in the control of pulmonary immune responses.

### Role of nitric oxide radicals in asbestos-induced lung inflammation

*G. Thomas, T. Ando, K. Verma and E. Kagan*
Departments of Pathology and Physiology and Biophysics, Georgetown University School of Medicine, Washington, D.C. 20007, USA

It has been shown recently that alveolar macrophages (AM) can synthesize nitric oxide (NO•), an effect which is inducible by interferon-γ. Because asbestos workers have elevated levels of intrapulmonary interferon-α, asbestos exposure conceivably may stimulate the production of NO* by AM. To test this postulate, pooled AM were obtained by bronchoalveolar lavage from Sprague–Dawley rats. Thereafter, $5 \times 10^5$ AM, suspended in RPMI 1640

medium containing 10% fetal bovine serum, were added to 24-well tissue culture plates. After allowing the AM to attach for 2 h at 37°C, the AM were incubated in 5% $CO_2$ at 37°C for 4–48 h in the presence (10 g/ml) or absence of either crocidolite (amphibole) or chrysotile (serpentine) asbestos fibres. Nitrite ($NO_2^-$) production, a measure of NO* formation, was assayed in conditioned medium by the Griess reaction. Confirmation of NO* production was shown by relaxation of rat aortic smooth muscle. Both types of asbestos exposure induced significantly greater $NO_2^-$ production than was produced by untreated cultures ($p < 0.001$). The presence of 250 or 500 IU/ml of recombinant rat interferon-γ markedly increased the synthesis of NO* by AM. However, the effects of asbestos and interferon-γ were synergistic, were statistically significant after crocidolite exposure ($p < 0.001$) and were seen maximally in 48 h cultures. The addition of the NO* synthetase inhibitor, *N*-monomethyl-L-arginine (500 μg/ml), significantly reduced $NO_2^-$ production by AM. Asbestos exposure is known to induce the formation of superoxide anion ($O_2^{\cdot-}$), but the addition of superoxide dismutase (150 units/ml) to asbestos-containing cultures markedly stimulated the production of NO* by AM. Since $O_2^{\cdot-}$ can react with NO* to form peroxynitrite anion ($ONOO^-$), the induction of NO* formation may represent a novel mechanism of asbestos-related injury.

### Platelet-activating factor-induced interleukin-6 production by alveolar macrophages involved both cyclooxygenase and 5-lipoxygenase pathways

*M. Thivierge and M. Rola-Pleszczynski*
Immunology Division, Department of Pediatrics, Faculty of Medicine, Université de Sherbrooke, Sherbrooke QC Canada JlH 5N4

We have recently shown that platelet-activating factor (PAF) could stimulate interleukin-6 (IL-6) production by rat alveolar macrophages (AM) and that its effect was dependent on 5-lipoxygenase (5-LOX) activity.[1] Because indomethacin was also capable of inhibiting PAF-induced stimulation of IL-6 production we investigated the potential involvement of both cyclooxygenase (COX) and 5-LOX pathways in these events and compared them with modulation of tumour necrosis factor (TNF) production by PAF. Treatment of AM with PAF concomitantly enhanced their IL-6, TNF, prostaglandin (PG) $E_2$ and leukotriene (LT) $B_4$ production two- to five-fold with peak effects at $1 \times 10^{-10}$M. Inhibition of COX with indomethacin, aspirin or ibuprofen ($1 \times 10^{-6}$M) abrogated the PAF-induced augmentation of both IL-6 and $PGE_2$ production, but further enhanced PAF-stimulated TNF production. In parallel, exogenous addition of $PGE_2$ ($3 \times 10^{-6}$M) or dibutyryl-cAMP ($1 \times 10^{-3}$M) to AM resulted in enhanced (five- to ten-fold) IL-6 production and a concomitant suppression of TNF production. On the other hand, PAF induced augmentation of IL-6 production was abrogated by the 5-LOX inhibitors AA 861 ($1 \times 10^{-5}$M) and MK 886 ($2 \times 10^{-6}$M), while TNF production was affected marginally. Exogenous $LTB_4$ markedly enhanced IL-6 (three- to five-fold) and $PGE_2$ (two-fold) production with a peak effect at $1 \times 10^{-12}$M, but stimulated TNF production to a lesser degree. In contrast, indomethacin abrogated the $LTB_4$ effect on IL-6 production, while further enhancing its effect on TNF production. PAF was also found to stimulate inducible COX mRNA accumulation, but had no effect on mRNA levels of either 5-LOX or 5-LOX-activating protein (FLAP). Taken together, our data indicate a differential regulation of cytokine production by COX and 5-LOX metabolites in response to PAF and suggest that PAF induces both positive and negative feedback mechanisms in its interactions with macrophages and their cytokine networks. These findings may have relevance in PAF-mediated events in the lung, such as the cellular components of late-phase asthma.

Supported by the Medical Research Council of Canada and the Fonds de Recherche en Santé du Québec.

**Reference**

1. *J Allergy Clin Immunol* 1992; **90**: 796.

### Compartmentalized response of alveolar macrophages in unilateral community acquired pneumonia

*M. Deboux, A. Boutten, J. Ostinelli, N. Seta, B. Crestani, M. C. Dombret, C. Rolland and M. Aubier*
Hôpital Bichat, Inserm U226, 46 rue Henri Huchard 75877 Paris Cedex 18, France

TNFα, IL-1β and IL-6 which can be released by peripheral blood monocytes (PBM) and alveolar macrophages (AM) are the main cytokines found during an inflammatory process. Many of their activities indicate a local rather than a systemic production. The aim of this study was to assess the response of AM and PBM to a local infection in patients with non-treated unilateral community acquired pneumonia (CAP). Nine patients suffering from a CAP with a unilateral consolidation or an infiltrate on chest X ray and without bilateral purulent bronchial secretion, were studied before antibiotherapy. During a bronchofibreoscopy, two small bronchoalveolar lavages (BAL) were performed: one in the non-infected side and then one in the territory of the pneumonia. A blood sample was simultaneously collected. BAL and blood from eight healthy volunteers served as control. The *in vitro* spontaneous and LPS-induced secretion of IL-lβ, IL-6, and TNFα of AM from the infected lung and from the non-infected lung and of PBM were studied (ELISA Medgenix). AM or PBM ($10^6$ cells/ml) were incubated for 18 h in the presence or absence of LPS (*E. coli* 10 μg/ml). When AM from infected lung were cultured in the absence of LPS, spontaneous release of TNFα, IL-lβ and IL-6 were higher than in the non-infected lung and than in healthy controls. No significant difference were found beetwen the spontaneous cytokine secretion of AM from the non-infected lung and from the healthy controls. After LPS-induced activation, cytokine secretion of AM was not significantly different beetwen the non-infected and the infected lung. Interestingly, a profound decrease of LPS-induced TNFα, and IL-6 production of AM was observed in CAP (non-infected $p < 0.05$; infected $p < 0.05$) as compared to healthy subjects. Cytokine secretion of unstimulated PBM from CAP were higher than healthy control ($p < 0.05$). After LPS activation cytokine secretion by PBM from CAP was higher than control but the difference between the two groups did not reach statistical significance. Our data indicate a compartmentalized response of AM within the lung and indicate an *in vivo* activation of both AM and PBM during CAP. Furthermore, the different responses of AM and PBM to an *in vitro* activation suggests that local cytokine production by AM during CAP are regulated differently than the systemic production by PBM.

### Eosinophils, cytokines and allergic inflammation

*R. Moqbel*
National Heart and Lung Institute, London SW3, UK

Eosinophils are prominent cells in the blood, sputum and bronchoalveolar lavage (BAL) of asthmatic subjects and eosinophil numbers have been shown to correlate with disease severity and the degree of bronchial hyperreactivity. The presence of eosinophils in the airways and their subsequent activation and exocytosis are thought to be important elements in the damage to the airways and the subsequent development of airway irritability. Eosinophils secrete a number of basic (cationic) granule proteins, following activation, which been shown to be cytotoxic to lung tissue. Activated eosinophils (i.e. undergoing exocytosis) are increased in numbers in association with asthma and bronchial hyperreactivity. Furthermore, stimulated eosinophils express and translate mRNA for a number of these inflammatory cytokines, including granulocyte/macrophage colony-stimulating factor GM-CSF, IL-3, IL-5 and IL-6. Human eosinophils can be specifically identified by immunostaining with mAb BMK-13, which binds to eosinophil granule major basic protein, thus providing total eosinophil counts at the site of inflammation and $EG_2$, which recognizes the secretory form of eosinophil cationic protein (activated eosinophils). There is now evidence to support the hypothesis that T cells, especially those of the helper phenotype, are involved in the immunological and inflammatory reactions associated with eosinophil-mediated allergic inflammation. T cells can influence the inflammatory process through the release of cytokines including interleukins-3, 4, 5 and the granulocyte/macrophage colony-stimulating factor (GM-CSF). IL-4 is an important cofactor required for the induction of IgE synthesis from B cells while IL-3, IL-5 and GM-CSF are important cytokines in the growth, maturation and terminal differentiation (IL-5) of eosinophils. They are also known to prime, activate and prolong the survival of eosinophils. Recent studies on bronchial biopsies and BAL of mild asthmatic subjects, using the technique of *in situ* hybridization, have demonstrated increase in the expression of mRNA IL-4 and IL-5, as well as IL-3 and GM-CSF, suggesting a pattern associated with $TH_2$-type lymphocytes. The precise source of IL-5 mRNA in sites of allergic inflammation has also been studied using a simultaneous combination of immunocytochemistry and *in situ* hybridization. These studies have suggested that $CD3^+$ cells were the principal source of IL-5 in nasal allergen-induced late-phase reactions, while mast cells and eosinophils, a small percentage of which expressed mRNA for IL-5, were less prominant. Thus, cytokines which are active on eosinophils, derived from T lymphocytes, eosinophils or mast cells, may play an important role in the orchestration, control and augmentation of the inflammatory reaction known to be associated with allergic responses.

### IgE-dependent activation of human granulocytes

*M. Capron, M.-J. Truong and A. Capron*
C.I.B.P., Unité Mixte INSERM U167 CNRS 624, Institut Pasteur, 1, rue du Prof. Calmette, 59019 Lille, Cédex, France

Eosinophils represent one of the major cell populations involved in IgE-dependent diseases such as asthma. Not only mast cells and basophils but also eosinophils and more recently neutrophils, can be activated by IgE antibodies in the presence of the specific allergen. Eosinophils express different types of functional IgE receptors, among which CD23 and the S-type lectin Mac2/εBP. They are involved in IgE binding and in IgE-mediated release of cytotoxic granule proteins. Recently, it has been shown that IgE-dependent activation of eosinophils could iduce the release of IL-5, the main factor for terminal differentiation of eosinophils, suggesting a local autocrine pathway of activation. More recently, we could show[1] that neutrophils, the only leukocyte population with no detectable Fcε receptor could express Mac2 and be activated by IgE antibodies and the specific antigen. The nature of mediators released by neutrophils upon IgE-dependent activation is presently under investigation. However, these results suggest that all granulocytes might participate directly to IgE-mediated mechanisms during inflammation.

### Reference

1. M. J. Truong *et al. J Exp Med* 1993; **177**: 243–248.

### Chemotaxis of eosinophilic granulocytes to cytokines in allergic disorders

*P. L. B. Bruijnzeel,[1] G. R. Dubois[2] and L. Koenderman[3]*
[1]Department of Pharmacology, Medical Biological Laboratory, Rijswijk, Norway; [2]Departments of Dermatology and [3]Pulmonary Diseases, University Hospital Utrecht, Utrecht, The Netherlands

Eosinophils are important effector cells in allergic inflammation. Increased numbers of activated eosinophils are found in allergic inflamed lesions. Cytokines a.o. IL-3, IL-5 and GM-CSF have been identified as important modulators and inducers of eosinophil chemotaxis. In this study we have investigated the chemotactic potency of these cytokines for eosinophils from the peripheral blood of normal individuals (N), allergic asthmatics (AA) and allergic atopic dermatitis (AD) patients using a microchemotaxis Boyden chamber assay. Optimal chemotaxis was reached for most cytokines at a concentration of 10 nM in all investigated groups with one exception; in AD patients the optimal chemotactic concentration for GM-CSF was already reached at 1 nM . Dose ranges of these chemoattractants demonstrated that eosinophils from AA generally showed a decreased response to these cytokines compared to eosinophils from N. In contrast eosinophils from AD patients showed an increased response. This phenomenon was most pronounced in case of GM-CSF. These findings indicate that different signalling pathways are initiated during priming of eosinophils *in vivo* in allergic inflammation, resulting in a different modulation of eosinophil responses towards cytokines.

### IL-4 induced mobilization of eosinophils in allergic inflammation

*G. R. Dubois and P. L. B. Bruijnzeel*
Department of Dermatology, University Hospital Utrecht, Heidelbergln 100, 3584 CX Utrecht, The Netherlands

Eosinophils are considered important effector cells in both asthma and eczema. They are abundantly present in e.g., lung tissue and show signs of activation. T lymphocytes present in allergically inflamed tissue synthesize and secrete the cytokines IL-3, IL-4, IL-5 and GM-CSF. IL-3, IL-5 and GM-CSF act as chemotaxins on eosinophils. However, IL-4 also may act as a chemotaxin on eosinophils. In contrast to the former cytokines IL-4 is only chemotactic for eosinophils from peripheral blood of patients with allergic disorders and not for eosinophils from normal individuals. IL-4 has the same chemotactic potency as the other cytokines. The optimal chemotactic potency is reached at a concentration of 10 nM. In contrast, neutrophils do not respond chemotactically to IL-4. Checkerboard analysis, inhibition studies with monoclonal anti-IL-4 Abs and desensitization experiments indicated specific interaction of IL-4 with eosinophils. In eosinophils from normal individuals IL-4 responsiveness could be induced by pretreatment of the cells with GM-CSF. Since IL-4 may be responsible for selective eosinophil trans-migration across the endothelium, IL-4 may further direct eosinophil migration and function within allergically inflamed tissue.

### Modulation of the enhanced migration of eosinophils from the airways of sensitized guinea-pigs: role of IL-5

*E. Coëffier, D. Joseph and B. B. Vargaftig*
Unité de Pharmacologie Cellulaire, Unité Associée Institut Pasteur-INSERM n° 285, 25, Rue du Dr. Roux, 75015, Paris, France

PAF, $LTB_4$ and interleukin (IL)-5 are potent chemoattractants for guinea-pig eosinophils,[1,2] which may be involved in eosinophil recruitment and upregulation in allergic diseases. Eosinophils from the bronchoalveolar lavage fluid (BALF) of guinea-pigs sensitized to ovalbumin (OA) were collected 24 h after antigen provocation, isolated on a metrizamide gradient and were studied with respect to migration by PAF, $LTB_4$ and rhIL-5. Total BALF content and distribution of eosinophils were greater ($p < 0.05$, $n = 35$) in sensitized, OA-challenged guinea-pigs ($5.0 \pm 0.8 \times 10^6$/guinea-pig; 12 ± 1%) than in sensitized saline-challenged animals ($3.0 \pm 0.7 \times 10^6$/guinea-pig; 7 ± 1%). Spontaneous, PAF and $LTB_4$-induced migration of eosinophils from sensitized and OA-challenged guinea-pigs were significantly enhanced, as compared to sensitized and saline-challenged animals (170 ± 36 *vs* 35 ± 9 migrating eosinophils for 10 nM PAF-acether; 271 ± 60 *vs* 110 ± 19 for 1 nM $LTB_4$). TRFK-5, an antibody to IL-5, reduced eosinophil recruitment in BALF of antigen-challenged sensitized animals as well as the enhanced responsiveness of eosinophils from the challenged animals, suggesting a role for IL-5 in the priming of eosinophils *in vivo*. Nedocromil sodium reduced to a similar extent eosinophil, macrophage and lymphocyte recruitment in the BALF of antigen-challenged and of saline-challenged sensitized animals, but failed to downregulate the enhanced responsiveness of eosinophils from the challenged animals. Our results support the concept IL-5 is essential for recruitment and priming of eosinophils from the guinea-pig BALF.

### References

1. E. Coëffier *et al. Int J Immunopharmacol* 1991; **13**: 273–280.
2. *J Immunol* 1991; **147**: 2595–2602.

### Effects of interleukin-5 inhibition on antigen-induced airway hyperresponsiveness and cell accumulation in guinea-pigs

*A. A. Y. Milne and P. J. Piper*
Department of Pharmacology, The Royal College of Surgeons, London WC2A 3PN, UK

Asthma is characterized by bronchial hyperresponsiveness (BHR) which is correlated with increased numbers of eosinophils in bronchoalveolar lavage fluid (BALF). Interleukin-5 (IL-5) has many actions specific for eosinophils, and is present in increased levels in BALF from asthmatics.[1] Blockade of IL-5 *in vivo* using the antibody TRFK5 results in a reduction of airway eosinophilia in guinea-pig models of airway inflammation[2,3] but the effect on BHR was not studied. We have used TRFK-5 to evaluate the importance of IL-5 and eosinophils in mediating BHR in antigen-induced airway inflammation in the guinea-pig. Animals were sensitized and challenged with ovalbumin, resulting in BHR to acetylcholine (Ach) (three-fold change in $EC_{50}$) and an increase in total cells (three-fold) and eosinophils (16-fold) in BALF 24 h post challenge. TRFK-5, or control antibody (rat IgG) were given 1 h before challenge (1 mg/kg i. v. $n = 5$). Control antibody had no effect on cell numbers in BALF, or on BHR compared to untreated animals. TRFK-5 reduced eosinophilia ($11.4 \pm 2.6$ to $2.8 \pm 0.9 \times 10^6$/ml (mean ± S.E.M.)) in BALF, and reduced BHR (airway resistance to 10 g/kg Ach, 1 141 ± 93 to 494 ± 96 cm $H_2O$/l per s) . These results confirm that IL-5 mediates the eosinophilia and show further that this cytokine is also involved in the development of BHR in this model.

**References**

1. Walker *et al. Am Rev Respir Dis* 1992; **146**: 109–115.
2. Chand *et al. Eur J Pharmacol* 1992; **211**: 121–123.
3. Gulbenkian *et al. Am Rev Respir Dis* 1992; **146**: 263–265.

### Effects of inhaled steroids on blood eosinophils in moderate asthma

*M. Laviolette, C. Ferland, L. Trépanier, H. Rocheleau, A. Dakhama and L.-P. Boulet*
Unité de recherche Centre de Pneumologie (Université Laval). Hôpital Laval, 2725 Chemin Sainte-Foy, Sainte-Fay, Québec, Canada G1V 4G5

Inhaled steroids could improve asthma by decreasing the bronchial release of chemotactic factors and cytokines and consequently inhibiting eosinophil activation and recruitment. We studied the effect of a low dose of beclomethasone on asthma symptoms, peak expiratory flow rates (PEFR), methacholine response (PC20), blood eosinophil counts and eosinophil leukotriene $C_4$ ($LTC_4$) production. Twelve asthmatics, using $\beta_2$ agonists on demand, were randomized in a double-blind cross-over study to take 400 μg Beclodisk® or a placebo twice daily for 6 weeks. Blood eosinophils were purified with a 1.078/1.100 g/ml Percoll gradient after a 10 min incubation with FMLP 10 nM. Mean (S.E.M.) eosinophil recovery and purity were 77 ± 4% and 97 ± 2%. Eosinophils were incubated ± GM-CSF (100 U) for 30 min and then ± 2 μM calcium ionophore A23187 for 15 min. $LTC_4$ was measured in the cell supernatants by EIA. Compared to placebo, inhaled steroids increased morning PEFR from 391 ± 30 to 472 ± 41 l/min ($p = 0.007$). decreased asthma symptoms and the $\beta_2$ agonist use from 3.1 ± 0.9 to 0.72 ± 0.5 daily inhalations ($p = 0.007$), increased PC20 methacholine from 0.38 to 1.9 mg/ml (geometric mean) ($p = 0.02$) and decreased blood eosinophil counts from $0.35 \pm 0.06$ to $0.26 \pm 0.04$ cells × $10^9$/l ($p = 0.084$). In the absence of A23187, eosinophils released no detectable $LTC_4$. However stimulation with ionophore provoked the release of large amounts of $LTC_4$ by eosinophils, and GM-CSF further enhanced the ionophore-induced $LTC_4$ production. Inhaled steroids decreased the eosinophil A23187-induced $LTC_4$ release from 5 571 ± 899 to 3 129 ± 462 pg/250 000 eosinophils ($p = 0.04$) and the GM-CSF-enhanced $LTC_4$ production, from 11 177 ± 1597 to 5950 ± 531 pg/250 000 eosinophils ($p = 0.003$). These results show that clinical asthma improvement is associated with a reduction in eosinophil $LTC_4$ production. Since the effects on blood eosinophils were obtained with a low dose of inhaled steroids. they were more likely due to the local effect of inhaled steroids on the bronchial mucosa than to a systemic effect.

Supported by 'l'Association pulmonaire du Québec' and Glaxo Canada Inc, 'Bureau d'affaires du Québec'.

### A role for cytokines in resolution and repair of pulmonary inflammation

*P. Henson*
National Jewish Center for Immunology and Respiratory Medicine, 1400 Jackson Street, Denver, Colorado 80206, USA

The inflammatory process is incomplete without the resolution and consequent repair of the tissue, optimally by an restoration of normal structure and function, but all too often by fibrosis. Since inflammation is characterized by influx of migratory cells, alterations in vascular permeability and usually by local tissue damage, the first step in resolution has to involve removal of fluid proteins, cells and cell debris—not to mention any microorganisms or exogenous material that might have initiated the response. It is suggested that incoming monocytes mature along a number of different and mutually exclusive pathways to orchestrate these processes. (1) Phagocytosis of digestible particulate matter leads to expression of new surface receptors for phosphatidylserine as well as to increased synthesis of TGF-β and PDGF by mechanisms that involve autocrine action of lipid mediators, TGF-β, PKC and perhaps engagement of surface integrins. The PS receptors are suggested to participate in removal of debris and cells that have undergone apoptosis a process that is accompanied by surface expression of phosphatidylserine. This could account for the removal of inflammatory cells from the lesion preparatory to the repair process. (2) Exposure of maturing macrophages to connective tissue elements such as hyaluronic acid induces on the other hand a pathway that leads to increased expression and release of IGF-1. Acting through CD44, this stimulus initiates its own cytokine-driven sequence involving the autocrine action of TNFα. (3) Stimulation with polyribonucleotides (or LPS) induces yet another pathway of maturation that results via mechanisms that involve calcium and tyrosine and serine/threonine phosphorylation in a cytocidal cell. Here too a requirement for autocrine TNFα action has been demonstrated, and yet the pathway is quite exclusive of that induced by hyaluronidate depending whether or not the cells synthesize interferon. In its presence the cytocidal pathway is induced by TNF, in its absence, cells that synthesize IGF-1. These various pathways can proceed independent of external cytokines because of the extensive autocrine feedback processes. However, they are also modulated by cytokines derived from other cells in the environment. Equally importantly, the cytokines and growth factors produced by the macrophages during the maturation diffuse into the inflamed lung to help orchestrate the processes of resolution and repair.

### Modulation of IL-1ra and TNF soluble receptors produced by alveolar macrophages and blood monocytes

*L. P. Nicod, B. Salve de Rochemonteix and J.-M. Dayer*
Division of Respiratory Diseases and Division of Immunology and Allergy, University Hospital, Geneva, Switzerland

Alveolar macrophages (AM) are not only able to produce several inflammatory substances such as IL-1α and β or TNFα, but also inhibitors of these same cytokines which are the IL-1 receptor antagonist (IL-1ra) and soluble receptors of TNF. Comparison of *in vitro* kinetics of IL-1ra, IL-1α and IL-1β in the presence of phorbol myristate acetate revealed that their mRNA expression was assynchronous. IL-1α and IL-1β mRNA were expressed after as little as 15 min, whereas IL-1ra mRNA was detectable after 3 h in culture. Twenty-four hours later, the amount of extracellular IL-1ra was equal to that of intracellular IL-1ra and was 30 and 100 times higher than IL-1α or β, respectively. In the presence of IL-4 or GMCSF IL-1ra was markedly increased whereas that of IL-1α or β was decreased or unchanged with the former and slightly enhanced with the latter.

AM were then tested for their ability to release the inhibitors of TNFα, the soluble receptors of 55 and 75 kDa. Although unstimulated AM express low levels of TNF-R55 and TNF-R75; they shed more TNF-sR75 as measured by ELISA. PMA stimulation increased mostly the expression and the release of TNFsR75 and had little effect on TNFsR55. Among the cytokines analysed IFN increased by three-fold the shedding of TNFsR75. This suggests that the expression of the two TNFsR are ruled by separate mechanisms and differ in shedding sensitivity.

Supported by the FNRS N° 31-31019.91 (LN) and 31-33786.92 (JMD)

### Cells and cytokines in chronic bronchial infection

*J. Eller, J. R. Lapa e Silva, H. Lode and P. J. Cole*
Host Defence Unit, Royal Brompton National Heart & Lung Institute, London, UK; Section of Infection & Immunology, Department of Pulmonology I, Krankenhaus Zehlendorf, Berlin, Germany; Servigo de Pneumologia, Hospital Universitario Clementino Fraga F° UFRJ, Rio de Janeiro, Brazil

Bronchiectasis is a well defined model of chonic bronchial sepsis. It is regarded as the inflammatory final common pathway from several causes. Over 60% of all cases are idiopathic and less than 5% result from immunodeficiencies. The bronchial secretions are usually infected with non-virulent bacteria. The damage to the bronchi seen in the condition is stimulated by the colonizing bacteria but it is induced by a host-mediated 'vicious circle' of inflammatory events. Progressive bronchial damage can result from direct effects of toxins released by luminal bacteria and subversion of the normally protective host inflammatory response to a damaging one. The host response is characterized by intense neutrophil traffic through the bronchial wall to luminal bacteria-laden mucus and by a cellular immune response within the affected bronchial wall. This cellular infiltrate is mainly composed of macrophages and activated T-lymphocytes, mostly CD8+ cells, some of them presenting cytolytic potential. In follicular bronchiectasis, CD4+ cells are found predominantly within the bronchial lymphoid aggregates. Production of cytokines by these cells is a likely possibility in this situation, some of which could have chemotactic activity for neutrophils and other inflammatory cells. We quantitated the cytokine profile in luminal secretions. Twelve consecutive bronchiectasis patients with acute infective exacerbations were treated with antibiotics. IL-1, TNF-α and IL-8 were measured by ELISA in plasma and sputum pre- and post-therapy. IL-8 was also determined 2 weeks after treatment. All patients showed a marked reduction in sputum volume, purulence and cell counts at the end of treatment. Cytokine levels in plasma were below the limit of detection. All three cytokines were found in bronchial secretions before treatment: IL-1 and TNF levels decreased after therapy but IL-8 levels remained raised, even 2 weeks into remission. We conclude that a major chemoattractant signal for neutrophil traffic in bronchiectasis remains raised, despite clinically effective antimicrobial therapy. The stimulus for IL-8 production and its source(s) in this condition remain to be determined.

### Cytokine gene and peptide regulation in lung microvascular injury: new insights on the development of adult respiratory distress syndrome (ARDS)

*R. Rabinovici, L. Neville and G. Feuerstein*
Department of Surgery, Jefferson Medical College, Philadelphia, PA 19107 and SmithKline Beecham, King of Prussia, PA 19406, USA

We have recently shown that the combined administration of non-injurious doses of LPS and PAF in the rat produce ARDS-like lung injury characterized by neutrophil adhesion to lung capillary venules, neutrophil accumulation in lung parenchyma, pulmonary oedema, and increased protein in BAL fluid. This new paradigm of lung injury was associated with elevated serum TNFα and pretreatment with anti-TNFα mAb dose-dependently prevented these responses. Also, the combined administration of LPS and PAF induced lung mRNA levels of TNFα (five-fold vs. LPS or PAF alone), IL-1β (80-fold), KC (60-fold) and IL-6. Taken together, these data suggest that this new paradigm of lung injury is cytokine-mediated and that LPS/PAF *in vivo* can functionally couple to the activation of gene expression of a multi-cytokine network system, all of which may be involved in the pathogenesis of ARDS.

### Relations between neutrophils and cytokines in patients with adult respiratory distress syndrome (ARDS)

*S. Chollet-Martin, P. Montravers, C. Gibert, C. Elbim, J. M. Desmonts and M. A. Gougerot-Pocidalo*
Laboratoire d'Immunologie et d'Hématologie et INSERM U 294, Services de réanimation, CHU X. Bichat, 75877 Paris Cedex 18, France

To gain further insight into the pathogenesis of ARDS, we studied possible relationships among the activation status of circulating neutrophils, cytokine levels and the severity of lung injury in 31 patients: 15 with ARDS, nine with severe pneumonia and seven mechanically ventilated with neither ARDS nor pneumonia. Using flow cytometry, we identified a subpopulation of neutrophils with an increased capacity to generate hydrogen peroxide after stimulation *ex vivo* in all patient groups; significantly higher values were found in those with ARDS. This was correlated with elevated TNFα levels in plasma and produced by cultured monocytes, and with elevated IL-8 levels in bronchoalveolar lavage fluid (BAL). The neutrophil activation status and TNFα parameters correlated with indices of the severity of lung injury, as IL-8 levels were related to a fatal outcome and the presence of shock. The BAL fluid to plasma ratio of IL-8 was significantly greater than that of TNFα, indicating higher local production of IL8. Thus, TNFα and IL-8-primed neutrophils may play a major role in the pathogenesis of ARDS-associated lung injury.

### The effect of airborne particulates on *in vitro* interactions of human basophils and neutrophils

*H. Behrendt, B. Hitzfeld and K. H. Friedrichs*
Medical Institute of Environmental Hygiene at the Heinrich-Heine-University, PO Box 103751, D-4000 Düsseldorf, Germany

The effects of extracts of airborne particulate matter (APM) on the morphology of and the mediator release from enriched basophils (HBC) and from purified neutrophils (PMN) of pollen allergic and non-allergic individuals were investigated. For this purpose APM was collected on glass fibre filter sheets using high volume samplers HVS150. After preparing an aqueous extract, in which allergens are absent, followed by extraction of organic substances, the mediator releasing activity of the APM extracts was studied under defined and physiological *in vitro* conditions.

The results indicate that APM potentiates anti-IgE induced histamine release (HR) and that this enhancement is significantly higher in HBC samples from pollen allergic donors than from non-allergic individuals. This is a non-cytotoxic event, it does not depend on extracellular calcium and does not affect the calcium dependency of HR induced subsequently by anti-IgE. Electronmicroscopy of APM exposed HBC reveals basophil degranulation in addition to strong activation of platelets and neutrophils, the latter sticking on to the surface of HBC and forming clusters with them. We therefore also investigated the effects of APM on mediator release of purified PMN. The results show that organic APM extracts preferentially induce $LTC_4$ and IL-8 release. Incubation of purified PMN with APM prior to zymosan stimulation showed that these substances also enhance the release of $LTB_4$, $LTC_4$, $PGE_2$ and IL-8.

This indicates that substances adsorbed to APM interfere with mediator cells of the allergic inflammation and exhibit 'priming' effects on them. Therefore, exposure of individuals to high concentrations of APM can provoke enhancement of symptoms of allergy and continuation of allergic inflammation.

### New therapeutical approaches in the treatment of asthma

*Ph. Godard*
Hopital Arnaud de Villeneuve, Montpellier, France

Bronchial asthma is a chronic inflammatory disorder of the airways. It's a disabling condition and all the epidemiological data indicate an increase of prevalence, morbidity and mortality. Despite the high efficiency of inhaled corticosteroids (ICS), there is a need for new therapeutical approaches. We need to increase our knowledge of the pathophysiology. During a long period of time, allergen challenge was the gold standard to test the efficacy of a new drug. However, it is clearly established now that chronic asthma is quite different and some drugs which can prevent or reverse allergic reactions are not very active on a chronic basis. Chronic asthma is an inflammatory disorder of the airways; the cellular influx is related to the severity of the disease; the vascular component of the disease could be linked to the activity, in other words to variability or instability of airway obstruction.

The inflammatory cascade is not well known. However, it can be hypothetized that resident cells are able to initiate it, either spontaneously or after specific or non-specific targeting. In that context alveolar macrophages could be good target cells for drug: their suppressive activity on lymphocytes is decreased; they express high quantities of HLA-DR and ICAM-1; specific antagonists could be tried; they appear to be in a high activation state (high releasibility); this could be the consequence of a priming effect or of an intrinsic defect (low intracellular cAMP, high turnover of phosphoinositides, translocation of the PKC could be good candidates). Mast cells are present in normal airways and it has been suggested that they could participate in the defence mechanisms. It could be proposed to act specifically at this level, for example by targeting aspecific drug. Epithelial cells are present all along the bronchial tree; it is clear that they do not act only as a passive barrier. They express abnormally high levels of ICAM-1. In animals, it has been shown that anti-ICAM was able to prevent eosinophil recruitment.

Corticosteroids are able to reverse airway obstruction, to clear inflammatory cells and to restore a nominal epithelial line. However, the repair is not totally satisfactory: the pseudo thickening of the basement membrane appears not to be modified; can it be suggested that inflammatory process has not to be totally abolished? their efficacy is not identical in all patients; the heterogeneity of airway inflammation has been suggested, but not fully investigated and not well understood. However, it can be thought that the importance of lymphocytes has to be taken into account; the results which have been obtained with cyclosporine sustain this hypothesis; there are some corticoresistant asthmatic patients; some authors suggested the role of specific cytokine; but additional data are required.

In the near future, the therapeutical approaches of bronchial asthma could be as follows: 1. better use of the drugs which are currently available; this means that we have to educate the patients and their doctor(s); 2. prevent the onset or worsening of asthma. Indeed environmental factors are critical and several possibilities of action exist at an individual and general level; 3. develop more effective ICS; this could be done by developing new ICS with more topical activity or which could be targeted to specific cell(s); 4. discover new drugs which could act at a specific cellular level in order to down-regulate its high releasibility; 5. introduce adjuvant drugs which could 'prime' the effect of corticosteroids at the receptor level.

### Perspectives and diagnostic potential for cytokines and adhesion proteins

*J. L. Gordon and A. J. H. Gearing*
British Biotechnology, Oxford, OX4 5LY, UK

The co-ordinated expression of cytokines and adhesion molecules (CAMs) is essential for the development of immune and inflammatory responses. There is considerable interdependence in the functions of cytokines and CAMs – for example, the expression of adhesion molecules E-selectin, VCAM-I and ICAM-I is regulated by the pro-inflammatory cytokines IL-1 and TNF, and the affinity of $\beta_2$ and $\beta_1$ integrins is increased following stimulation of leucocytes with chemotactic cytokines such as MIP-1β and GMCSF. Recent experiments also show that MIP-1β bound to the adhesion molecule CD44 on endothelial cells can stimulate lymphocytes to upregulate the affinity of their 14 integrin for its endothelial ligand VCAM-1.[1]

The potential diagnostic or prognostic value of measuring blood levels of cytokines has been explored in a number of pathological states but the significance of these measurements can be limited because cytokines are usually produced (and act) locally, and are cleared rapidly from the circulation. Specific inhibitors of cytokines can also be measured in the blood (e.g., soluble receptors for TNF and IL-1, which can be shed from the surface of cells), and measuring the balance between cytokines and their inhibitors may be more meaningful than measuring cytokine concentrations alone.

We speculated that soluble CAMs might also be released from cell surfaces, and by using specific ELISAs and immunoprecipitation analysis we have demonstrated that endothelial cells can be stimulated to release soluble ICAM, VCAM and E-selectin. These soluble CAMs are all found in the serum or plasma of healthy individuals, and levels of some or all of them can be elevated in renal, vasculitic, diabetic, transplant and septic shock patients, and during cytomegalovirus infection. The cellular sources of these molecules, and the mechanism(s) by which they are released, have not yet been determined, but in some instances the levels measured apparently reflect the clinical prognosis.

### Reference

1. Tanaka *et al. Nature* 1993; **361**: 79–82.

### Correlation between membrane marker CD23 — soluble CD23 and cytokines in atopic patients

*A. Sabbah and C. Buchman*
Laboratoire d'Immuno-Allergologie, CHU 49033 ANGERS Cedex 01, France

IgE has two types of receptors: high affinity RFcΣI and low affinity RFcΣII. The latter may be studied by flow cytometry by the membrane marker CD23 (mCD23), expressed on B and T cells monocytes, macrophages, Langerhans cells, eosinophils and platelets. Soluble CD23 (sCD23) corresponds with the extracellular C-terminal domain of mCD23.

The aim of the study was to search for a correlation between the levels of mCD23 and sCD23, comparatively with IgE, eosinophils, eosinophil cationic protein (ECP) and to the cytokines IL-2, IL-4 and IL-6. Seventy-eight patients took part in the study, divided into two groups:

group 1: atopics ($n$ = 61)
group 2: controls ($n$ = 17)

The majority presented with rhinitis, asthma, atopic dermatitis isolated or associated. The results showed a highly significant correlation between CD23 and sCD23 compared with the control group. There was also a statistically significant increase for mCD23, sCD23, cytokines IL-2, IL-4 and IL-6, mediator ECP in parallel with eosinophils and total IgE.

The study confirms amongst other things the rise in IL-4 in atopy and the correlation between the level of ECP and that of eosinophils, and that there is an overall correlation between mCD23 and sCD23

### Inhibition of GM-CSF gene transcription by glucocorticoids in the Jurkat T cell line: selection of resistant clones from Jurkat cells treated with methylprednisolone

*M. Bossé, M. Laviolette and M. Audette**,
Unité de recherche, Centre de pneumologie de l'Hôpital Laval, Réseau de Centres d'excellence en Santé Respiratoire/Laboratoire d'Endocrinologie Moléculaire*, Centre de Recherche du CHUL, Ste-Foy, Québec, Canada

In asthma, T cells modulate the inflammatory response by producing haematopoietic cytokines like IL-3, IL-5 and

GM-CSF. Glucocorticoids are potent anti-inflammatory drugs, they inhibit the transcription of various cytokine genes including IL-2, IL-5, IL-6. However, the dexamethasone inhibition of the synthesis of IL-2 by mitogen-stimulated T lymphocytes was significantly decreased in asthmatic subjects resistant to glucocorticoid treatment. First, we verified the transcriptional inhibition of GM-CSF by methylprednisolone (MP) with RT-PCK in the Jurkat T cell line. T cells were incubated 20 h with mitogens (PHA 10 g/ml, PMA 5 ng/ml) in RPMS 10% at $10^6$ cells/ml with concentrations of MP ranging from $10^{-5}$ to $10^{-9}$M. Total RNA was immediately extracted by the guanidium chloride method. One μg of total RNA was used for reverse transcription (5 units r*Tth*, 200 mM NTPs, 1 mM $MnCl_2$, 100 pmol of the reverse primer 5′GCACAGGA-AGTTCCGGGGT3′, 1 min 95°C; 2 min 50°C; 15 min 72°C). PCR reaction was performed using the forward primer 5′GCTGCTGAGATGAATGAAAC3′ (100 pmol) in the following conditions: 10 mM $MgCl_2$, 30 s 95°C, 30 s 55°C, 1 min 72°C, 30 cycles, 10 min 72°C. The amount of PCR product evaluated by ethidium bromide staining showed a dose dependent inhibition and was barely detected at $10^{-5}$M of MP. Second, in attempt to establish an *in vitro* model of steroid resistance, Jurkat T cells were incubated at $10^5$ cells/ml with $10^{-3}$M of MP sodium hemisuccinate and grown up to $10^6$ cells/ml. MP-treated Jurkat cells were maintained for up to 15 passages. The inhibition of mitogen-induced proliferation of the MP-treated cells was compared to untreated Jurkat cells. The MP-treated cells were less inhibited by steroids. The transcription of GM-CSF evaluated by RT-PCR was not inhibited up to $10^{-4}$ M of MP. The transcription of GM-CSF was inhibited by MP in the Jurkat T cells. The pool of MP-treated T cells was resistant to the glucocorticoid according to mitogen-induced proliferation and GM-CSF transcription activity, fourteen clones were obtained from this MP resistant pool. In conclusion, glucocorticoids inhibit gene transcription of GM-CSF and we obtained resistant clones to study the molecular mechanisms of the resistance to glucocorticoids.

### Azelastine modulates T cell function

*N. Chand and R. D. Sofia.*
Wallace Laboratories, Cranbury, NJ 08512 USA

T cell recruitment, proliferation and activation in the lungs play a critical role in the pathogenesis of chronic asthma. Drugs which interfere with the proliferation, blastogenesis and/or activation of T cells may be effective in the management and treatment of chronic asthma. Azelastine (40 ng/ml, 12 h preincubation with dendritic cells) inhibited antigen (*Dermatophagoides farinae*, 10 μg/ml) induced IL-2 responsiveness (proliferation response) in T lymphocytes obtained from asthmatic children. Azelastine (40 ng/ml) also inhibited purified protein derivative-induced IL-2 responsiveness in normal human peripheral blood lymphocytes but did not influence Con-A-induced expression of IL-2 responsiveness. These data demonstrate that azelastine exerts a specific T cell, suppressive effect. It appears to act on an antigen-presenting pathway.[1] It, also inhibited LPS-stimulated IL-1 generation in human monocytes.[2] Azelastine (1-100 μM, 3–5 day incubation) inhibited food allergen (ovalbumin)-induced blastogenesis of lymphocytes obtained from bronchial asthmatics complicated with atopic dermatitis ($IC_{50}$<5 μM). Azelastine (5–500 μM, 24 h preincubation) also inhibited IL-2-induced T cell blastogenesis in IL-2-dependent NKC-3 cells ($IC_{50}$<5 μM). However, it did not influence antigen-induced IL-2 generation in the peripheral blood monocytes.[3] The chemotactic activity of calcium pyrophosphate-induced pleural exudate on rat T cells was also inhibited by azelastine and dexamethasone. Both the drugs also inhibited IL-1 release in pleural exudate and leukocyte chemotactic activity in mice.[4] Furthermore, azelastine inhibited aeroallergen induced (T cell-dependent, IL-5-mediated) bronchial eosinophilia in guinea pigs.[5] Therefore, these activities of azelastine would suggest a steroid-sparing effect in asthmatics.

**References**

1. *Jap J Allergol* 1990; **39**: 1509.
2. *Agents & Actions* 1991; **32**: 243.
3. *Alergy Ptact* 1991; **11**: 44.
4. *Agents & Actions* 1991; **32**: 56.
5. *Intl Arch Allergy Immunol* 1992; **97**: 229.

### Effect of cyclosporin A on the infiltration of T lymphocytes and eosinophils in lungs of sensitized rats

*F. Kyriacopoulos, C. Carré, V. Lagente, J-M. Mencia-Huerta and P. Braquet*
Institut Henri Beaufour, 1 avenue des Tropiques, 91952 Les Ulis, France

The immunosuppressive compound cyclosporin A has been demonstrated to inhibit lung eosinophil infiltration induced by antigen challenge in sensitized guinea-pigs (Lagente *et al.*, submitted), suggesting a link between activation of T lymphocytes and the recruitment of this cell type. The effect of treatment of the animals with cyclosporin on the eosinophil and T lymphocyte accumulation and activation induced by antigen challenge in the lung of sensitized rats, was investigated. Brown Norway rats were sensitized by i.p. and s.c. injections of ovalbumin (OA) in $Al(OH)_3$, at a 15-day interval. Seven days after the last injection, aerosol challenge was performed. Histological preparations of lung specimens using Luna staining showed a marked and significant increase of eosinophils in lung tissue 24–96 h following OA challenge. Pretreatment of the rats with cyclosporin (10 mg/kg twice a day for 2 days and 10 mg/kg orally, 1 h before OA challenge totally inhibited the accumulation of eosinophils observed in the lungs of sensitized rats at 48 h. Cyclosporin also reduced the increase in eosinophil number in the bronchoalveolar lavage fluid after OA challenge (54% inhibition). In order to characterize the T lymphocyte subsets possibly involved in this process, cryostat sections of lung tissue were stained by various mouse monoclonal antibodies. Antigen challenge of sensitized rats provoked a significant accumulation of CD3+, CD4+ and CD25+ T lymphocytes in lung tissue that was inhibited when the animals are treated with cyclosporin. These results demonstrate that cyclosporin treatment reduces the antigen-induced accumulation of eosinophils and T lymphocytes in sensitized rats and further strengthen the fact that T lymphocyte activation and eosinophil recruitment are related events.

### Effects of FK-506 and nedocromil sodium on the bronchial inflammatory infiltrate of actively sensitized guinea-pigs

*J. R. Lapa e Silva, D. Baker, R. J. Scheper, B. B. Vargaftig, M. Pretolani*
University Hospital, Federal University of Rio de Janeiro, Brazil; Unité de Pharmacologie Cellulaire, Institut Pasteur, Paris, France; Royal College of Surgeons of England; Free University, Amsterdam.

The bronchial wall from guinea-pigs sensitized by 2 s.c. injections of ovalbumin at a 2 week interval exhibit marked infiltration by CD4+ T-lymphocytes/eosinophils. We have now investigated the effects of nedocromil sodium and of FK-506, a novel immunosupressive agent, on the bronchial inflammation in sensitized guinea-pigs. Four groups of six Hartley guinea pigs received s.c.injections of 10 g ovalbumin in 1 ml $Al(OH)_3$ at day 0 and 14, and were killed at day 21: two groups received from day 14 s.c. injections of FK-506 (1 mg/kg) or nedocromil sodium (30 mg/kg) daily. The other two groups received daily injections of the diluent (ethanol+Tween 80 or saline, respectively). The animals were killed, the lungs frozen and cryostat sections stained with immunohistochemical methods using monoclonal antibodies: CT5= T cells, macrophages; H159=T cells; H155=CD4; CT6=CD8. Cyanide-resistent eosinophil peroxidase activity (EPO) was used to stain eosinophils. Sections were coded, and positive cells were enumerated in the lamina propria:

| | CT5 | H159 | H155 | CT6 | EPO |
|---|---|---|---|---|---|
| FK-506 | 10.9(1.8) | 8.0(0.9) | 6.7(1.1) | 7.1(0.6) | 14.8(0.8) |
| DIL | 42.1(5.8) | 42.7(1.3) | 43.3(4.9) | 22.8(3.1) | 29.2(4.8) |
| p value | <0.05 | <0.01 | <0.01 | <0.01 | <0.05 |
| NEDO | 37.5(8.7) | 41.3(1.3) | 40.7(6.3) | 26.7(6.1) | 24.5(3.5) |
| DIL | 39.7(6.3) | 52.2(4.4) | 47.1(5.8) | 20.7(3.1) | 51.3(7.3) |
| *p* value | NS | NS | NS | NS | <0.05 |

mean (SEM) of positive cells per unit area Student's *t*-test.

FK-506 abolished the T-cell/eosinophil invasion in the bronchial wall, whereas nedocromil sodium was ineffective in protecting the lungs from T-cell infiltration, although it reduced the eosinophil influx. These findings suggest that independent mechanisms may be involved in the recruitment of eosinophils to the bronchi in this model.

### Reference

1. Lapa e Silva *et al. Am Rev Respir Dis* 1992; **145**: A39.

### Evidence of cytotoxic activity of CD8+ T lymphocytes in the chronic inflammatory infiltrates of bronchiectasis

*J. R. Lapa e Silva, L. W. Poulter, P. J. Cole*
University Hospital, Federal University of Rio de Janeiro; Royal Free Hospital School of Medicine, London, UK; Host Defence Unit, Royal Brompton National Heart & Lung Institute, London, UK.

The chronic bronchial inflammatory response in bronchiectasis (Bx) includes a large number of T-lymphocytes, many CD8+ cells with evidence of activation and immunological commitment (Lapa e Silva et al. Am.Rev.Respir.Dis.1992; 145: A335) . The question as to whether these CD8+ T cells possess cytolytic potential and damage the bronchial mucosa or possess suppressor function and control the T cell response in the mucosa, has not been clarified. We have investigated the expression of a protein, recognized by a novel monoclonal antibody (TIA-1), which may distinguish cells exhibiting cytolytic activity (Anderson et al., Immunol.1990; 144: 574) in the cellular infiltrate of Bx. Bronchial biopsies were obtained from 12 Bx patients and six controls (C), snap frozen in isopentane cooled by liquid nitrogen, and cryostat sectioned for conventional histology and immunohistochemistry. Consecutive sections were stained with CD8 or TIA-1 using immunoperoxidase. Double immunofluorescence was employed to study co-expression of both antigens, using fluorescein and rhodamine as fluorochromes. The positive cells were counted in the bronchial lamina propria:

| | CD8 | T\IA-1 | CD8/TIA-1* |
|---|---|---|---|
| Bx | 7.9 (0.8) | 1.9 (0.3) | 16.0 (1.7) |
| c | 1.2 (0.3) | 0.03 (0.01) | 0.9 (0.2) |
| *p* | <0.0001 | < 0.001 | < 0.001 |

mean (S.E.M.) positive cells per unit area; *mean (S.E.M.) % double-positive cells; *p* values= Student's *t*-test

A significant increase in the co-expression of CD8 and TIA-1 was seen in Bx. However, expression of TIA-1 was not restricted to the CD8+ cells, but was also found in putative CD4+ cells. These findings are compatible with a proportion of CD8+ T cells having cytolytic potential in Bx and could account for some of the damage seen in this disease.

### Reference

1. Lapa e Silva *et al. Am Rev Respir Dis* 1992; **145**: A335.

### The immunosuppressive activity of riminophenazines is mediated by lysophospholipids

*R. Anderson*
Department of Immunology, University of Pretoria, South Africa.

The relationship between the phospholipase-stimulating and immunosuppressive properties of the riminophenazine antimycobacterial agent clofazimine and its experimental analogue, B669, has been investigated *in vitro*. At concentrations of 0.6 μM and upwards, both agents caused dose-related inhibition of mitogen- and alloantigen-stimulated uptake of tritiated thymidine by human mononuclear leukocytes (MNL), while in short-term assays both agents, particularly B669, caused dose-related enhancement of the release of lysophosphatidylcholine (LPC) and arachidonic acid from these cells. Arachidonate *per se* at concentrations of 20 μM did not affect mitogen-activated lymphocyte proliferation, while cyclooxygenase and 5'-lipoxygenase inhibitors as well as water- and lipid-soluble oxidant scavengers and anti-oxidant enzymes failed to protect the cells against the anti-proliferative effects of clofazimine and B669. However, LPC caused dose-related inhibition of lymphocyte proliferation. Moreover, co-incubation of MNL with alpha-tocopherol (Vitamin E), a lysophospholipid-complexing agent, or with lysophospholipase, protected the cells against clofazimine and B669, as well as against LPC. $Na^+$ $K^+$ ATPase was identified as the primary target of riminophenazine/LPC-mediated inhibition of lymphocyte proliferation. Excessive release of anti-proliferative lysophospholipids during clofazimine or B669 treatment of mitogen- or antigen-activated lymphocytes, leading to inhibition of $Na^+$, $K^+$-ATPase activity, is the probable biochemical mechanism of the immunosuppressive activity of these agents.

### Ribomunyl modulates the expression of DR and IL-2R antigens on blood cells in patients with severe immune disorders

*I. Khomenko and V. Chukanov*
Central TB Research Institute, Yauzskaya alley, 2, Moscow, 107564 Russia.

The aim of the investigation was to assess Ribomunyl's (Pierre Fabre) influence on the expression of 'activation' antigens ($DR^+$and IL-$2R^+$) on blood cells of patients with severe immune disorders (secondary). Thirty-two adult patients with active, sputum-positive lung tuberculosis were under observation. Immunological blood testing in the method of indirect immunofluorescence revealed severe secondary immune disorders: decreased $CD3^+$ content (42.3 ± 1.4), $CD4^+/CD8^+$ ratio (1.2 ± 0.06) and increase in $DR^+$ and IL-$2^+$ cells (36.8 ± 2.1 and 14.2 ± 1.7 respectively). In the treatment of 20 patients in addition to antituberculosis chemotherapy Ribomunyl was applied (group 1), while other 12 patients received only chemotherapy. The effect of Ribomunyl application on immune indices was confirmed by repeated blood testing. In group 1 the level of $CD3^+$ lymphocytes was normalized, $CD4^+/CD8^+$ ratio considerably improved, the levels of 'activated' lymphocytes decreased ($DR^+$ cells from 36.8 ± 1.3 to 21.0 ± 1.4; $p < 0.05$; IL-$2R^+$ cells from 14.2 ± 1.1 to 7.3 ± 1.0; $p < 0.05$) in comparison with that in the group receiving only chemotherapy. The data given above approve Ribomunyl application in patients with severe secondary immune disorders.

### Interleukin-2 therapy in the treatment of malignant lymphoma

*A. A. Umnyashkina, F. M. Abdullayev and R. F. Umnyashkina*
Institute of Phtisiology and Pulmonology, Quarter 2514, Zardabi Street, Baku 370IIS, Azerbaijan Republic.

Interleukin-2 (IL-2), produced by stimulated lymphocytes, is thought to hold promise in the treatment of malignant lymphoma. Twenty-eight patients with mean age 19.5 years affected by malignant lymphoma were studied. All received intravenous IL-2 (30 000 to 70 000 U/kg) every 8 h for 9–12 days. All patients had major adverse reactions to IL-2, but only one had to withdraw from the trial. In addition to multiple laboratory abnormalities, at least 80% had fever, chills, cough, anorexia, malaise, hypotension, or skin rash. Overall clinical improvement was modest. The 15 patients improved dramatically during therapy but relapsed within 8 weeks. Seven patients had a 4-month remission, the increase of a number of CD1, CD21 lymphocytes, phytohaemagglutinin-induced blastogenesis and normalization of CD4/CD8 indices was detected. These outcomes, especially the side effects, are disappointing for a therapy that once held high hope. On the basis of these results, it is possible to conclude that perhaps long-term therapy with lower doses of IL-2 in patients with malignant lymphoma will prove more valuable.

### Mast cell heterogeneity and hyperplasia in crocidolite-induced pulmonary and pleural fibrosis of rats

*M. Respondek,[1] B. Voss,[2] T. Kereyni,[1] Th. Wiethege,[2] and K.-M. Müller.[1]*
[1]Institute of Pathology, Bergmannsheil Hospital, Ruhr University Bochum and [2]Professional Associations' Research Institute for Occupational Medicine at the Ruhr University Bochum.

The presence of mast cells in lung is of considerable interest because these cells contain a wide variety of chemical mediators of importance in health and disease. Although pulmonary mast cell hyperplasia has been identified in lung fibrosis, the histochemical and functional characteristics of these mast cells have not been analysed. By means of different experimental animal studies we tried to investigate the formal and causal pathogenesis of crocidolite fibres-related pulmonary and pleural tissue reaction pattern. Groups of eight animals received a single intratracheal instillation of saline buffered crocidolite (2 mg crocidolite) or physiological saline as control. Animals were sacrified 1, 3, 7, 14, 38, 64, 119, 169, 206, 364, 400 and 540 days after instillation of crocidolite. Lungs were proceeded for light and electron microscopical examinations. The mast cell density was significantly increased in the rat alveolar wall, the subpleural parenchyma and in the diffuse sclerosing pleura. The mast cell hyperplasia occurs on day 38 after the single intratracheal instillation of crocidolite until at least day 540 . In the lung parenchyma of crocidolite treated rats we found between 20 and 125 mast cells per middle power field (obj. × 20), in contrast to less than five mast cells in the control group. The lung parenchymal and pleural mast cells were histochemically of the connective tissue type (CTMC). Interestingly, CTMC showed a chronic process of partial degranulation which differs from that found in anaphylaxis. In summary, thus pronounced mast cell hyperplasia occurs during the evolution of experimental pulmonary fibrosis. This model provides a powerful tool to study pulmonary mast cells and to identify their role in fibrotic disease.

### Dexamethasone diminishes stem cell factor-induced serotonin release by rat peritoneal mast cells

*A. M. Taylor, S. J. Galli*, and J. W. Coleman,*
Department of Pharmacology, University of Liverpool, P.O. Box 147, Liverpool L69 3BX, UK and *The Departments of Pathology, Beth Israel Hospital and Harvard Medical School, Boston, MA 02215, USA

Stem cell factor (SCF), the ligand of the c-*kit* receptor, is an important mast cell growth and differentiation factor that has recently also been shown to exert direct or synergistic mediator releasing effects on tissue mast cells *in vivo* or *in vitro*. Accordingly, SCF may modulate mast cell function during tissue hypersensitivity reactions. We have investigated the effects of culture *in vitro* with or without a corticosteroid on the responsiveness of mast cells to stimulation by SCF.

Rat peritoneal mast cells were purified to >95% through 72.5% Percoll and cultured at 1–5 × $10^5$/ml in complete DMEM (containing 5% FCS) at 37°C in 5% $CO_2$ for 1 or 24 h, beforè challenge for 30 min at 37°C with recombinant rat SCF (4–500 ng/ml). The cans released a substantially greater proportion of granule-associated [$^3$H]serotonin (5-HT) after 24 h compared to 1 h of culture in drug-free medium (100 ng/ml SCF gave 28.1 ± 9.8% 5-HT release after 24 h of culture compared with 0.6 ± 0.2% release after 1 h of culture; $p < 0.05$). The development of responsiveness to SCF was virtually abolished by the presence of $10^{-7}$M dexamethasone during the 24 h culture period. The drug caused 85% inhibition of 5-HT release induced by 100 ng/ml SCF compared to 50% inhibition of release induced by either 48/80 (2.5 g/ml) or anti-IgE (1/100). Our observation that the acquisition by mast cells of responsiveness to SCF during culture is reversed by dexamethasone suggests that *in vivo* corticosteriods may down-regulate inflammatory responses induced by this cytokine.

### Mechanism of mast cell TNFα-dependent cytotoxicity and its modulation by sulfasalazine

*E. Y. Bissonnette, J. A. Enciso, and A. D. Befus.*
Department of Medicine, University of Alberta, Edmonton, AB, Canada T6G 2S2.

Mast cells (MC) are an important source of the multipotent cytokine tumour necrosis factors (TNFα). We investigated the mechanism of action of MC derived TNFα-dependent cytotoxicity (WEHI-164 target) and its modulation by anti-TNFα and sulfasalazine. Rat peritoneal MC (PMC) were pretreated for 4 h with and without anti-TNFα and/or sulfasalazine, washed to eliminate free anti-TNFα or sulfasalazine and tested for their cytotoxic activity. Sulfasalazine pretreatment inhibited by 49% PMC cytotoxicity and anti-TNFα pretreatment reduced PMC cytotoxicity by 64%, raising the possibility of active TNFα on the membrane of PMC as demonstrated on macrophages. To further investigate this hypothesis, PMC were fixed with 1% paraformaidehyde. Fixed PMC exhibited TNFα-dependent cytotoxicity (48.6 ± 6.3 $LU_{20}/10^6$ compared to 136.2 ± 8.2 $LU_{20}/10^6$ for control) without releasing TNFα, but which could be eliminated by anti-TNFα in the assay. Interestingly, the binding of anti-TNF to membrane TNF increased the levels of mRNA for TNFα, suggesting that the binding of membrane TNF to its receptor on the target cell stimulates TNF mRNA in PMC. By contrast, sulfasalazine treatment inhibited by 77% the levels of mRNA for TNF. Thus, PMC cytotoxicity is mediated by an increase in the levels of mRNA for TNFα and by soluble and membrane TNFα. Sulfasalazine treatment reduces TNFα-dependent cytotoxicity by depressing the levels of mRNA for TNFα as well as the release of TNFα.

Supported by Alberta Heritage Foundation for Medical Research and Medical Research Council, Canada.

### TNFα release during systemic reaction in cold urticaria

*I. Tillie-Leblond, P. Gosset, A. Janin, B. Wallaert and A. B. Tonnel*
Institut Pasteur Lille, France.

Primary cold urticaria (PCU) characterized by urticaria, and sometimes a shock-like reaction after cold exposure, is usually considered to be linked with histamine and $PGD_2$ release by mast cells. To determine the involvement of mast cell cytokines, we studied the release of Tumor necrosis factor alpha (TNF) in the blood after immersion of the hand in chilled water. Five patients with PCU were compared to a control population. Among patients with PCU, submitted to the cold immersion test, two exhibited a shock-like reaction with a large urticarial plaque, one only a mild cutaneous reaction and two did not have any reaction. Patient 1 was reevaluated after treatment by antihistamine; he did not respond to this challenge. All controls were strictly negative. Histamine was released within the first minute following the challenge in the three patients with urticaria. TNF was undetectable in the blood of the patient with only a mild cutaneous reaction, whereas a TNF release was observed in the blood of two patients with a systemic reaction, 2 and 6 min after the challenge, suggesting that the secreted TNF was a preformed protein. The two other patients, the patient 1 under treatment and the control subjects released neither histamine nor TNF. Pathological and immunohistochemical (with a rabbit anti-TNF antibody) studies were performed on skin biopsies collected 10 min after ice-cube test (ICT). Four patients with PCU presenting a positive ICT were studied as well as the patient after treatment with antihistamine which did not respond to the ICT. Sections stained with MGG revealed that mast cells were the only cells infiltrating the dermis. Ultrastructural studies demonstrated that most mast cells were degranulated in the four patients with positive ICT, whereas they were still quiescent in patient 1 with a negative ICT. Quantitative studies showed that 90.2 ± 5% mast cells were stained with anti-TNF antibody using cryocut sections of the four patients and patient 1 under treatment. No other cells were positive for TNF. Moreover, the detection of TNF in the blood seems to be related to the urticarial plaque area, since similar numbers of degranulated TNF positive mast cells per field were found in all four patients although two of them did not develop a shock-like reaction. This study demonstrates a release of TNF by mast cells in a clinical situation, as already observed in experimental data. Since hypotension has been demonstrated after TNF infusion, this cytokine might be implicated in the shock-like reaction in addition to histamine in cold urticaria.

### Production of cytokines in a human mast cell line

*M. Buckley [1], J. Thompson [2], J. Coleman [1], K. Ray [2] J. H. Butterfield [3].*
[1]Department of Pharmacology, University of Liverpool, P.O. Box 147, Liverpool L69 3BX, England, [2]Department of Cellular Science, Glaxo Group Research, Greenford Rd, Greenford, Middlesex, UB6 OHE, England; [3]Department of Allergic Diseases and Internal Medicine, Mayo Clinic, Rochester, Minnesota, 55905, USA.

Activation of mast cells leads to the release of a number of mediators such as histamine, tryptase, prostaglandin $D_2$ ($PGD_2$), leukotriene $C_4$ ($LTC_4$), and platelet activating factor (PAF). These can produce many of the phenomena seen in the acute asthmatic response such as bronchoconstriction, oedema, and excessive mucus production. Studies with mouse mast cells have shown that these cells produce multiple cytokines, and these have been implicated in the late phase of allergic responses. Using the techniques of reverse transcription and the polymerase chain reaction (PCR) we have detected the mRNA for interleukins-3, 4, and 6 (IL-3, 4, and 6) granulocyte–macrophage colony stimulating factor (GM-CSF) and tumour necrosis factor alpha (TNFα) in an unstimulated human mast cell line, RMC-1. This cell line does not appear to express a functional high affinity IgE receptor as demonstrated by lack of IgE binding and response to IgE priming followed by triggering with anti-human IgE. However, the production of IL-3, IL-4, IL-6 and GM-CSF mRNA appeared to be increased when the cells were activated with the calcium ionophore ionomycin. The time course for this induction spanned several hours but appeared to peak by 4 h in the case of IL-3, IL-6, and TNF. The HMC-1 cells also express mRNA encoding cytokine receptors for IL-4, interferon gamma (IFN-γ), and stem cell factor (SCF). Overnight treatment of these cells with IL-4 resulted in an enhancement of their constitutive and ionophore-induced production of mRNA for IL-3, IL-4, IL-6, and GM-CSF.

The potential of human mast cells to produce cytokines supports the concept that these cells may be able to promote the late phase reaction of asthma, which is characterized by inflammation, infiltration by eosinophils, and airway hyperresponsiveness. In summary, these results suggest that human mast cells can act as a source of, and target for, cytokine action.

### Neuropeptides and cytokines in magnesium deficiency

*W. B. Weglicki, I. T. Mak, R. E. Stafford, B. F. Dickens, M. M. Cassidy and T. M. Phillips,*
Departments of Physiology and Medicine, The George Washington University Medical Center Washington, DC 20037, USA

Significant lesions develop in the heart and other tissues of young animals fed a diet deficient in magnesium (Mg). We have identified a role for oxygen derived free radicals in the pathobiology for this disorder.[1,2] Diminished RBC glutathione levels, increased serum lipid peroxidation and tissue protein oxidation occur. More recently we have discovered that the inflammatory cytokines (IL1, IL6, TNFα) are elevated significantly[3] during the third week of Mg-deficiency, when cardiovascular lesions are prominent. During the first week of Mg-deficiency we found significant serum elevations of the neuropeptides, substance P and calcitonin gene-related peptide. Microdissection revealed significant elevations of substance P in cardiac lesions suggesting an association of this neuropeptide with local inflammatory changes.[4] We have used several interventions to retard lesion formation: antioxidants, e.g. Vitamin E diminishes lesion formation without affecting circulating cytokines or Mg; substance P-receptor inhibition results in the inhibition of lesion formation as well as a significant decrease in TNFα levels. Our present hypothesis involves a role for neuropeptides, cytokines and oxygen-derived free radicals in the pathobiology of Mg-deficiency.

**References**
1. *BBRC* 1990; **170**: 1102.
2. *Am J Phys* 1992; **262**: 1376.
3. *Mol Cell Biol* 1992; **110**: 169.
4. *Am J Phys* 1992; **263**: 734.

### Changes in mast cell number and phenotype after syngeneic lung transplantation in the rat

*N. Frossard,[1] A. Buvry,[2] M. Garbarg,[3] V. Dimitriadou,[3] A. Rouleau,[3] G. F. J. Newlands,[4] R. Tavakoli,[1] V. Poaty,[1] A. Lockhart,[1] J.C. Schwartz[3]*

[1]UFR Cochin Port-Royal, [2]UFR Bobigny and [3]INSERM U109, Paris, France and [4]Moredun Research Institute, Edinburgh, UK

Transplantation causes the total section of vascularization and innervation followed by repairing processes. An increase in mast cell number is generally associated with tissue repair. We have studied the number, phenotype and distribution of mast cells 1 month after syngeneic unilateral left lung transplantation in the Lewis rat, without interference of inflammation, graft rejection or of any treatment (Tavakoli *et al. Am J Physiol* 1992, **242**: L322-6). Connective and mucosal mast cell phenotypes were characterized using antibodies directed against their specific rat mast cell proteases, RMCPI and RMCP2, respectively. Tissue levels of proteases were determined by ELISA. Localization of mast cell phenotypes was studied by immunofluorescence after double immunostaining on two sections and three levels per lung. Results obtained in left lungs from five transplant recipients and five control animals were compared. RMCPI and RMCP2 levels (13.1 ± 1.2 and 5.6 ± 1.0 μg/g in controls, respectively) increased by 172% and 239% after transplantation. The number of mast cells (cells/length unit) increased from 1.3 in controls to 6.3 after transplantation for RMCPI-immunoreactive (IR) cells and from 5.7 to 17.7 for RMCP2-IR cells. The increase was significant a) around bronchioles and arterioles for both subtypes, b) around large vessels and in the pleura for RMCPI-IR cells and c) in smooth muscle layer of large airways for RMCP2-IR cells. Some mast cells expressed both enzymes. Their number increased from 1.4 in control lung to 7.1 after transplantation: the increase was significant around bronchioles and arterioles and in the pleura. No significant increase of intraepithelial mast cells was observed. In conclusion, an increase in the number of both connective and mucosal mast cell phenotypes occurs in the lung after transplantation. Mast cells expressing both intragranular proteases also increased after lung transplantation, indicating the possibility of an intermediary state between connective and mucosal mast cells. This suggests a local differentiation/maturation of mast cells in the lung after transplantation. The function of mast cell phenotypes during the repairing processes after transplantation remains to be clarified.

This work was supported by INSERM (90-0503).

### Interleukin 8 mRNA expression in sheep alveolar macrophages experimentally infected with ovine lentivirus

*G. Cordier, I. Legastelois, T. Greenland, P. Arnaud*[1] *and J.F. Mornex*
Laboratoire d'Immunologie et de Biologie Pulmonaire (Hôpital Louis Pradel), et INSERM U80, Lyon-France and [1]Department of Microbiology and Immunology, Medical University of South Carolina, Charleston, SC, USA

Interstitial lung disease can be observed after infection by most lentiviruses including HIV-1 in human and visna-maedi virus (VMV) in sheep. The VMV-induced disease is a model to decipher the mechanisms of lung tissue damage. Both naturally and experimentally infected animals develop lung pathology showing peribronchovascular lymphoid nodules, lung parenchymal damage and alveolitis. In previous studies we demonstrated the alveolitis to be lymphocytic and neutrophilic and that alveolar macrophages release a neutrophil chemotactic activity. Interleukin 8 (IL-8) belongs to the chemokine superfamily and has been shown to be chemotactic for neutrophils and lymphocytes. In order to analyse the expression of IL-8 by sheep alveolar macrophages following natural and experimental *in vivo* and *in vitro* infection, we cloned and sequenced an ovine IL-8 cDNA probe after retrotranscription of LPS-activated macrophages mRNA and amplification of a fragment using primers established from consensus regions in the human and rabbit sequences. This probe showed strong positive hybridization signal on Northern blots prepared with RNA from LPS-activated macrophages, *in vitro* virus-infected cells and cells from naturally infected animals but less for untreated cells. IL-8 could account for at least a part of the neutrophilic and lymphocytic alveolitis observed early after infection.

### Modulation of interleukin 8 gene expression and protein production in human monocytes: effect of silica and lipid mediators

*M.-J. April, S. Denault and J. Stankova*
Immunology Division, Dept. of Pediatrics,, Faculty of Medicine, University of Sherbrooke, Sherbrooke, QC, J1H 5N4, Canada.

Silica is an inorganic dust well-known to cause inflammation and fibrosis of lung tissues. The phagocytosis of silica particles by pulmonary macrophages leads to the release of various substances which can affect other immune cells. IL-8 is a potent chemotactic factor for neutrophils and T-lymphocytes, produced by a variety of blood and tissue cells. This cytokine is believed to play an important role in the development of inflammation by its property of cell recruitment. In this report, we have examined the modulation of IL-8 mRNA expression and protein production in human monocytes stimulated with silica. Northern blot analysis demonstrated a concentration-dependent increase of IL-8 mRNA accumulation and protein production after *in vitro* stimulation with silica. This increase was partially dependent on endogenous prostaglandin (PG) production since the inclusion of indomethacin caused a noticeable reduction of IL-8 mRNA accumulation induced by silica. Morover, exogenously added $PGE_2$ induced an increase in IL-8 mRNA accumulation. Inhibition of 5-lipoxygenase activation did not change silica-induced IL-8 mRNA expression and exogenous $LTB_4$ did not modulate IL-8 mRNA accumulation. Another lipid mediator, platelet-activating factor (PAF), whose production can be induced by silica, augmented IL-8 mRNA expression in a time and concentration-dependent manner. PAF upregulated IL-8 mRNA with a peak at 5 h after stimulation, with 100 pM PAF showing a maximal increase. PAF-induced IL-8 mRNA accumulation was prevented with WEB 2170, a PAF receptor antagonist. Nuclear run-on experiments showed that PAF increased IL-8 gene transcription within 30 min. Experiments using actinomycin D suggested that PAF also slightly augmented (10–20%) IL-8 mRNA half-life. These studies indicate that silica and PAF modulate IL-8 gene expression and thus may contribute to IL-8 production at sites of pulmonary inflammation.

Supported by MRC and FRSQ.

### The effect of st andardized *D. farinae* immunotherapy on cytokine production in asthmatics

*C. Jones, E. Milgrom, Z. Haddad.*
Los Angeles, California, USA.

The purpose of this study was to evaluate the effects of allergen desensitization on cytokine production by peripheral blood mononuclear cells (PBMC) *in vitro*. Initially *in vitro* cytokine production in response to *D. farinae* (df) antigen was evaluated comparing allergic asthmatic patients ($n$=9) and non-allergic non-asthmatic control subjects ($n$=6). Peripheral blood mononuclear cells (PBMC) were cultured with and without df antigen, recovered, and stained by immunochemical techniques for the presence of IL1B, IL6, GMCSF, and IFNG. Lymphocytes and monocytes were counted independently to obtain a percentage of each cell type staining positive for the presence of cytokine above baseline levels. The allergic asthmatic patients were randomly assigned into two groups in a double blinded fashion. One group underwent a course of allergen desensitization with *D. farinae* antigen ($n$=5), and the other group received equal volume of placebo ($n$=4). After completion of the injection schedule culture experments were repeated, and cytokine production was reevaluated in response to the same specific df antigen. Allergic asthmatic patients demonstrated significantly higher baseline presence of IL1B, IL6, and GMCSF in response to co-culture with the df antigen as compared to non-allergic non-asthmatic controls. No difference was observed in IFNG levels. Following the course of desensitization injections, antigen stimulated cytokine production changed in the following ways: 1. Antigen treated patients showed a significant decrease in the production of IL6 and GMCSF by both lymphocytes and monocytes when compared to pretreatment levels. 2. With the exception of IL1B, no significant change in cytokine presence was seen in the placebo treated group. 3. Antigen treated patients showed significantly less monocyte IL6 and GMCSF production after treatment than the placebo group. 4. Antigen treated patients showed a trend toward suppression of IL6 and GMCSF production; there was a higher level of spontaneous cytokine present in the absence of antigen than when antigen was added *in vitro*. This pilot study supports the concept that specific allergen desensitization may affect the inflammatory process of allergic disease by decreasing and possibly suppressing immunoregulatory cytokine production as suggested by the cytokine data obtained using (specific) antigen stimulated PBMC.

### Interleukin-6 production by a pulmonary epithelial cell line (A549 cell).

*B. Crestani;[1] P. Cornillet;[2] M. Dehouxo;[1]*

*C. Rolland,[1] M. Guenounou,[2] M. Aubier.[1]*

[1]INSERM U226, Faculté X. Bichat, Paris, [2]Lab. d'Hématologie, Hôpital R. Debré, Reims, France.

Regulation of type II pneumocytes proliferation after injury is unknown. IL6 is a broad spectrum cytokine with growth-promoting effects. An autocrine effect of IL6 has been shown in myeloma cell lines. The aim of this study was 1) to characterize the regulation of the gene and protein expression of IL6 in A549 cells and 2) to detect any growth-promoting effect of IL6 on A549 cells. A549 cells grown to confluence were stimulated with LPS, recombinant human (rh) IL1β, TNFα, or interferon γ (IFN-γ). Supernatants were assayed for immunoreactive IL6 (ELISA) and biologically active IL6 (B9 cellproliferation assay). Total RNA was extracted, Northern-blots were performed and hybridized with $^{32}$P-IL6 cDNA. [$^3$H]-thymidine incorporation measured A549 cell proliferation. Unstimulated A549 cells did not express IL6-gene nor secrete IL6. IL1β strongly induced IL6-gene expression. The effect was maximal 2 h after stimulation and at 2 ng/ml IL1β concentration. Immunoreactive IL6 was maximal 4 h after stimulation and at 2 ng/ml IL1 concentration. Time course for biologically active IL6 was similar. TNFα was a much less potent inducer of IL6 gene expression and IL6 secretion (10 fold smaller than IL1β-induced).

LPS-stimulated human alveolar macrophage-conditioned media (AM-CM) strongly induced IL6-gene expression and IL6 secretion. This effect was mediated by IL-1β and TNFα since IL-6 mRNA expression was abolished when A549 cells were incubated with AMCM + anti-IL1β and anti-TNFα antibody. In some experiments, A549 monolayers were stimulated for 24 h with LPS (0.1–100 μg/ml) or IFNγ (0.02–20 ng/ml) or a combination of IFNγ (20 ng/ml) and LPS (0.1–100 g/ml). LPS alone did not induce the expression of IL-6 mRNA, whereas IFN induced a small expression of IL-6 mRNA. However, the combination of IFN and LPS induced a dose-dependent expression of IL-6 mRNA by A549 cells. rhIL6 (up to 20 ng/ml) did not induce IL6-gene expression. rhIL6 (0, 0.01, 0.1, 0.5 ng/ml) did not modify [$^3$H]-thymidine incorporation (192 ± 12, 207 ± 23, 196 ± 10, 202 ± 16 × $10^3$ dpm/dish respectively; mean ± SEM, $n$=4).

We conclude that IL6 gene expression and protein secretion by A549 cells is signal specific and that IL6 has no growth-promoting effect on A549 cells.

### Interleukin-5 production by pulmonary T-cell clones: inhibition by immunosuppressive drugs cannot be assessed with the B13 bioassay

*F. H. Krouwels, R. Lutter, H. M. Jansen and T. A. Out*
Clinical Immunology Laboratory and Department of Pulmonology, Academic Medical Centre, Meibergdreef 9, 1105 AZ, Amsterdam, The Netherlands.

Interleukin-5 (IL-5) is presumed to be an important cytokine in the regulation of the pulmonary inflammation in patients with allergic asthma (AA). We have studied the production of IL-5 by airway and peripheral blood derived T-lymphocyte clones (TLC) of AA patients and controls including the effects of the immunosuppressive drugs dexamethasone (DEX) and cyclosporin A (CyA). TLC were simulated with mAb αCD3 or αCD2+αCD28+PMA. IL-5 was measured both with a sandwich ELISA and with the B13 bioassay. In particular the application of the ELISA enabled us to study the effects of DEX and CyA. Results: 1) TLC ($1 \times 10^6$/ml) produced IL-5 ranging from 1.26 to 73.5 ng/ml (as measured in the ELISA). As soon as 16 h after stimulation IL-5 production up to 42 ng/ml was found. 2) IL-5 activity in TLC supernatants measured with the ELISA correlated closely with IL-5 activity in the B13 assay (Spearman rank test: rho = 0.86, $p < 0.001$). 3) The proliferation of B13 cells, induced by rhIL-5, was inhibited both by DEX and CyA. The drugs did not influence the ELISA, which means that only the ELISA can be used in evaluating modulation of IL-5 production by DEX and CyA. 4) DEX (375–1500 ng/ml) and CyA ($10^{-7}$M) inhibited IL-5 production by TLC up to 82% as measured in the ELISA. We conclude that lung TLC are able to produce IL-5. The inhibition of IL-5 production by DEX and CyA may contribute to the overall effects of these drugs on pulmonary inflammation in patients with asthma.

### Simple method for measuring *ex-vivo* cytokine production by alveolar macrophages

*R. M. Perenboom, P. Beckers, R. W. Sauerwein, A. van Schijndel, M. F. van de Broek, J. W. M. van der Meer*
Departments of General Internal Medicine and Medical Microbiology, University Hospital Nijmegen, Geert Grooteplein zuid 8, 6525 GA Nijmegen, The Netherlands.

To study cytokine production by alveolar macrophages in HIV seropositive patients, we developed a system for *ex-vivo* production, with minimal manipulation of the HIV positive specimen, thus minimizing accidental contact with HIV contaminated material and minimizing potential contamination (and subsequent unwanted stimulation) of the specimen. An additional advantage is that this simple, practical method can also be applied in developing countries where advanced laboratory technology is not readily available and HIV-related pulmonary problems pose immense problems. Broncho-alveolar lavage material, collected via a closed system is transported to the laboratory on ice. There the bottle is opened, 30 μl is taken for cell count and the remaining fluid is centrifuged at 1 200 **g** for 10 min. The supernatant is removed and frozen at -20°C until assay; the pellet is resuspended in Dulbecco's modified Eagle's medium at a concentration of $0.5 \times 10^4$ macrophages per ml; the tube is closed again and incubated with and without stimulant at 37°C for 24 h Following incubation the tubes are centrifuged at 1200 **g** for 10 min and the supernatant and cell pellets separately frozen until cytokine analysis can be performed. Data will be presented on cytokine production in whole blood cultures and these alveolar lavage cultures from healthy volunteers and patients with *Pneumocystis carinii* infection.

### Dehydroepiandrosterone and androstenediol: potential use in lung diseases

*R. M. Loria and D. A. Padgett.*
Virginia Commonwealth University, Medical College of Virginia Department of Microbiology and Immunology, Richmond, Va 23298, USA.

The levels of the steroid hormone dehydroepiandrosterone (DHEA) decline with ageing in humans and mammals, and has been associated with deterioration of the immune condition. Previous reports have shown that DHEA (5-androsten-3β-17-one) may function *in vivo* to regulating host immune resistance to protect mice from a variety of lethal infections. This includes, but is not limited to, infection with viruses (herpesvirus type 2, coxsackievirus B4-CB4). bacteria (*Enterococcus faecalis, Pseudomonas aeruginosa*), and parasites (*Cryptosporidium parvum*). We reported that androstenediol (5 androsten -3β-17β-diol, AED), derived from DHEA, in the skin and the brain, is strikingly more effective in up-regulating systemic resistance against CB4 infection than DHEA. Compared to DHEA, treatment with AFD was markedly superior in protecting mice against virus-induced myocardiopathy, pancreopathy, and mortality. In particular, AED prevented virus-infected target tissues from destruction by cellular infiltrates. Neither steroid, has any significant direct antiviral effects. The effects of DHEA's function *in vitro*, specifically on mitogen-induced spleen cell proliferation, were examined. The data illustrated that DHEA significantly decreased $^3$H-thymidine incorporation by ConA-stimulated spleen cells in a dose-dependent manner. However, the inhibition associated with DHEA was not as potent as that observed with hydrocortisone. Inhibition of spleen cells proliferation cultured with both DHEA and hydrocortisone was not additive and did not exceed the inhibition achieved with hydrocortisone alone. The *in vitro* results indicate that DHEA retains significant 'glucocorticoid' properties. Based on the data showing that AED is significantly more effective in regulating the *in vivo* immune response, it is likely that DHEA is converted to a more active metabolite(s) for its function as an immune up-regulator. In conclusions, these steroids may have significant potential for use in the treatment of lung diseases.

### The estimation of specific immunotherapy in asthmatic children

*L. S. Namazova and V. G. Ivanov*
Institut of Pediatrics RAMS/109172, Gontcharnaya nab., 3, 8, Russia.

The specific immunotherapy (SIT) is the most specific and pathogenetic treatment of bronchial asthma in children.

But due its long duration it's possible to estimate its efficacy only in course of time. We supposed that it was necessary to find some indexes which. were capable of showing the SIT efficacy in different stages of this treatment. So we examined in dynamics 27 treated asthmatic children: 12 by mite allergens (*Dermatophagoides pteronyssinus*) by parenteral way, eight by bacterial allergens (*Streptococcus pyogenus, Staphylococcus aureus*) by endonasal way, seven by corticosteroids as other immunotherapy group and 20 healthy children as controls. Treatment efficacy was estimate after its end. We investigated blood levels of IL-1, IL-2, 2-microglobulin (2-MG), LTB4, IgE, PgE, PgF2, C3a-C4a-C5a-des-Arg (by RIA Kits, 'Amersham', UK and ot.), IgA, IgM, IgG, CIC, E-ROC, EA-ROC (by classical studies) and IL-1, production by monocytes (in biological test). We found that IL-2 was a very informative index; its blood level in asthmatic chlldren was in 2.5–3 times more than in controls. After corticosteroid therapy IL-2 level decreased (and was not different from controls). SIT caused IL-2 level increasing after the first dilution (1:1 000 000), but its dynamics was dlfferent in groups of effective (1) and low-effective (2) SIT (in first group IL-2 level slowly decreased. in second group - increased). Good efficacy of therapy we observed if we began SIT in the moment of high blood level of IL-2. Other, immunological figures had different dynamics in these two groups too. There was the extension of the correlations number between all these figures after the SIT. We suppose it shows the inclusion of some compensative immune mechanisms during immunotherapy in children.

### Upregulation of lung fibroblast-derived PDGF A-chain and its alpha receptor by asbestos fibres

*J. A. Lasky, J. C. Bonner, A. L. Goodell and A. R. Brody*
National Institute of Environmental Health Sciences, Research Triangle Park, N.C., 27709, U.S.A.

We are investigating the role of platelet-derived growth factor (PDGF) in a model of asbestos-induced fibrosis in rats and mice. PDGF, a potent mitogen for mesenchymal cells, is a 30–40 kDa glycoprotein dimer composed of A and/or B chains. Mesenchymal cells possess alpha and/or beta PDGF receptors which dimerize and differentially recognize the three PDGF dimer isoforms. Mesenchymal cells and tumour cells have been shown to secrete PDGF *in vitro* and an autocrine growth response has been proposed for this mitogen. Because asbestos fibres interact with mesenchymal cells at the site of the fibrotic lesion *in vivo*, we investigated whether chrysotile asbestos alters PDGF secretion or PDGF receptor expression in rat lung myofibroblasts *in vitro*. An *in vitro* autocrine role for PDGF in rat lung fibroblasts (RLFS) exposed to asbestos was demonstrated. Medium and cell lysates from RLFs exposed to asbestos contained more PDGF than unexposed RLFs as detected by Western blot. We believe that the 36 kDa protein band is PDGF-AA since we could not detect any PDGF in a sensitive ELISA which recognizes only PDGF BB or AB isoforms, and because we could detect PDGF A-chain mRNA, but not PDGF B-chain mRNA on Northern blots. We are currently searching for PDGF B-chain mRNA in our RLFs by combining reverse transcription, PCR amplification and Southern blotting. In addition to detection of A-chain PDGF mRNA we also found on Northern blot an upregulation of the mRNA which codes for the PDGF alpha receptor, i.e., the only receptor type to which A-chain PDGF binds. Concordantly, there was an increase in the normally low PDGF alpha receptor protein according to a radioreceptor assay. A significant dose-dependent mitogenic effect was found when our RLFs were exposed to chrysotile asbestos under serum free conditions *in vitro*, and this effect was significantly blocked with an anti-PDGF antibody. Whether PDGF A-chain and its alpha receptor are upregulated in lung mesenchymal cells *in vivo* following asbestos inhalation is currently under investigation.

### Resistance to recombinant interferon alpha-2A in patients with pulmonary tuberculosis

*R. F. Umnyashkina, F. M. Abdullayev, A. A. Umnyashkina*
Institute of Phtisiology and Pulmonology, Quarter 25I4, Zardabi Street, Baku 370II8, Azerbaijan Republic.

To explain the haematologic deterioration occasionally observed during interferon therapy, we assayed serum specimens from 81 patients with pulmonary tuberculosis receiving treatment with recombinant interferon alpha-2a for the presence of anti-interferon antibodies. After a median of 6 months of therapy, anti-interferon antibodies were found in 33 patients. Seventeen of these patients had only non-neutralizing antibodies, but antibody from the other I6 neutralized the antibacterial effects of recombinant interferon alpha-2a *in vitro*. In no case, however, did neutralizing antibody inhibit the antibacterial effects of purified natural interferon alpha. Clinical resistance to interferon of various degrees was present in seven of 16 patients with neutralizing antibodies; the remaining nine patients and all 19 patients without antibody continue to respond after a minimum of 1 year of therapy. In all the patients with interferon resistance, antibody was present when it developed. These data suggest that the development of clinical resistance to interferon alpha-2a in pulmonary tuberculosis is not necessarily related to an altered cellular response to interferon. Treatment with other interferons, such as purified natural interferon alpha, may be useful in patients with clinically important neutralizing antibodies against interferon alpha-2a.

### The endotoxin bronchial challenge: an ethically acceptable model of acute lung inflammation in human subjects?

*O. Michel and R. Sergysels*
Clinic of Pneumology, Saint-Pierre Hospital (ULB), rue Haute 322, 1000 Brussels, Belgium.

An inflammatory response is associated with several lung diseases; its modulation is the main goal of related therapy. Endotoxins are potent pro-inflammatory substances present in domestic house dust[1] and responsible for several occupational lung diseases.[2] In asthmatics, 20 μg inhaled endotoxin induces a significant bronchial obstruction with an increase in nonspecific bronchial hyperreactivity, in absence of any clinical response.[3] This lung function response is associated with a blood inflammatory response (neutrophilia and rise in CRP)[4] (146: 352-357) and is inhibited after pretreatment with an anti-inflammatory agent, sodium cromoglycate (submitted). The lung function response to endotoxin is independent of the atopic status but is associated with the degree of non specific bronchial responsiveness.[5] Nevertheless, in normal subjects ($n = 8$) inhaled endotoxin induces a blood inflammatory response (activation with increase in neutrophils and rise in CRP and ACTH (as marker of IL-1) - submitted) in absence of any lung function response.

Thus, 20 μg inhaled endotoxin induces a blood inflammatory response without clinical effect in both asthmatics and normal subjects and, or the other hand, a lung function response (blocked with cromoglycate) in asthmatics. To confirm the validity of this model, the blocking effects of anti-inflammatory agents on the LPS-induced blood inflammation could be evaluated.

**References**

1. Michel O. *Clin Exp Allerg* 1991; **21**: 441–8.
2. Rylander. *Am J Ind Med* 1990; **17**: 1–148.
3. Michel O. *JAP* 1989; **66**: 1059–64.
4. Michel O. *ARRD* 1992; **146**: 352–57.
5. Michel O. *Thorax* 1992; **146**: 352–57.

### Intrapulmonary immune activation during CMV pneumonitis complicating lung transplantation

*M. Humbert, J. Cerrina, G. Simonneau, P. Galanaud and D. Emilie*
INSERM-UI31, 32, rue des Carnets, 92140 Clamart, France.

**Methods.** The immune status of lung recipients displaying cytomegalovirus (CMV) pneumonitis or allograft rejection was analysed in 44 lung recipients after the third post-operative week. The parameters studied included (i) serum markers of immune activation [neopterin/creatinine ratio (Neo/Creat), soluble interleukin-2 receptor (sIL-2R), and IL-6]; (ii) IL-6 concentration in the epithelial lining fluid (ELF); (iii) gene expression of monokines (IL-1β, and IL-6), and cytotoxic mediators (granzyme B, and perforin) analysed by *in situ* hybridization (ISH) in alveolar cells collected by bronchoalveolar lavage (BAL).

**Results.** (i) serum Neo/Creat ratio, sIL-2R levels, and IL-6 levels were increased during CMV pneumonitis [818 ± 78 (mean ± S.E.M.) mol/mol, 451 ± 85 pmol/l, and 61.2 ± 11.5 U/ml, respectively], as compared to rejection events (190 ± 27 mol/mol, $p<0.001$, 179 ± 27 pmol/l, $p<0.02$, and 59.3 ± 20.5 U/ml, NS, respectively), and controls (158 ± 47 μmol/mol, $p<0.001$, 159 ± 32 pmol/l, $p<0.01$, and 24.2 ± 3.3 U/ml, $p<0.01$, respectively); (ii) IL-6 concentration in ELF was increased during CMV pneumonitis (1 022.3 ± 467.5 U/ml ELF), as compared to rejection events (508.9 ± 111.3 U/ml ELF, $p<0.05$), and controls (241.6 ± 63.5 U/ml ELF, $p<0.02$); (iii) The IL-1β, the IL-6, the granzyme B, and the perforin genes were expressed in most samples from patients with CMV pneumonitis (898 ± 449, 92 ± 74, 37 ± 19, and 19 ± 4 gene-expressing cells per 10 000 cells, respectively). This gene expression was always significantly higher than that observed during rejection or in controls.

**Conclusion.** CMV pneumonitis is associated with a significant systemic and intrapulmonary activation of both cytotoxic cells (presumably, NMC-restricted cytotoxic T lymphocytes, and non-MHC restricted natural killer cells) and monokine-producing cells (presumably macrophages). Although contributing to the limitation of viral spreading, these activated cells may also be involved in acute and chronic post-transplantation lung damage.

### Bradykinin-induced increase in cytosolic free calcium in tracheal smooth muscle cells via B2 receptors: effects of pertussis toxin and various cytokines

*Y. Amrani, M. Mousli, Y.Landry and C. Bronner*
Laboratoire de Neuroimmunopharmacologie, INSERM CJF 9105, Faculté de pharmacie, BP 24, 6 7401 Illkirch Cedex, Strasbourg.

Bradykinin is an inflammatory peptide which induced bronchoconstriction only in asthmatic patients but the reason for this as well as the precise mechanism of action of bradykinin are still unclear. It has been reported that cytokines, such as tumour necrosis factor, can modulate calcium signal induced by bradykinin in various cell systems. Moreover it is well known that smooth muscle contraction depends essentially on the concentration of cytosolic calcium. Therefore, we have examined the possibility that cytokines generated by inflammatory cells can modulate bradykinin induced rise in cytosolic free calcium at the smooth muscle level and by this way contribute to explain the sensitivity of asthmatic patients to bradykinin.

For this purpose, we have developed tracheal smooth muscle culture which provides a novel preparation in studying the molecular mechanism underlying bradykinin-induced bronchoconstriction. In Fura-2 loaded guinea-pig tracheal smooth muscle cells, bradykinin ($10^{-8}$) induced an increase of cytosolic free calcium which is either completely inhibited by the B2 antagonist Hoel40 ($10^{-8}$M) or treatment with Pertussis toxin (500 ng/ml) for 6 h. In contrast, a 24-h pretreatment with interferon γ (1 000 U/ml), or interleukin-6 (1 000 U/ml) and tumour necrosis factor (20 ng/ml) did not inhibit or enhance calcium signal.

These results show that bradykinin induced calcium mobilization via B2 receptors coupled to a G-protein sensitive to Pertussis toxin and that this rise is not affected bv a pretreatment with the different tested cytokines. Further investigations are undertaken on the effects of cytokines on bradykinin-induced calcium mobilization in tracheal smooth muscle cells, including combination of different cytokines.

### *In vivo* role of adenylate cyclase-haemolysin and pertussis toxin during *Bordetella pertussis* infection

*N. Khelef,*[1] *C. M. Bachelet,*[2] *B. Vargaftig*[2] *and N. Guiso*[1]

[1]Unité de Bactériologie Moléculaire et Médicale et [2]Unité de Pharmacologie Cellulaire. Institut Pasteur. 28 rue du Dr. Roux, 75724 Paris Cedex 15. France.

*Bordetella pertussis*, the agent of whooping cough, synthesizes and secretes factors thought to play a role in the adhesion of the bacteria on to the respiratory epithelium, such as the filamentous haemagglutinin (FHA), pertactin, agglutinogens. Other factors such as PTX, an ADP-ribosylating toxin, dermonecrotic toxin, tracheal cytotoxin, or adenylate cyclase-haemolysin (AC-Hly) are involved with toxicity. PTX induces *in vivo* many biological effects, such as lymphocytosis, but the *in vivo* activity of AC-Hly is not well defined. Using a murine respiratory model, we have shown that AC-Hly is absolutely required by the bacteria to initiate infection whereas PTX is required for colonization.

After infection with virilent *B. pertussis* strains or adhesin$^-$ mutants, infected mice (like infected children) have a pronounced lymphocytosis. This lymphocytosis is due to PTX since it is not observed after infection with PTX$^-$ mutants and followed injection of purified PTX. However, we observed an increase in total cell number in bronchoalveolar lavage fluids of infected mice with an increase in macrophage number and an influx of polynuclear neutrophils and lymphocytes. This effect was not observed after infection with PTX$^-$ mutants but surprisingly, also not after infection with AC-Hly$^-$ mutants, which express PTX. This result confirms the role of AC-Hly in the initiation of infection and demonstrates that PTX is responsible for lymphocytosis, but is not the only factor involved in the influx of polynuclear neutrophils and lymphocytes in bronchoalveolar lavage fluids.

### Adhesion molecules in AIDS. Increased expression of ICAM-1, decreased expression of HLA-DR on AM

*K. Dalhoff, S. Bohnet, H. Kothe, J. Braun and K. J. Wießann.*
Department of Internal Medicine, Medical University, Lobeck, Germany.

AM are the primary cell targets chronically harbouring HIV-1 in the lung. The mechanisms leading to the enhanced ability of macrophages to serve as AC in AIDS-patients are still incompletely understood. In addition to soluble mediators like IL-1 and IL-6 molecules of the MHC class II complex and adhesion molecules like ICAM-1 play a major role in the regulation of these cellular interactions. We investigated ICAM-1 and HLA-DR expression on AM from 13 AIDS patients in context with other parameters of macrophage activation comparing the results to a group of healthy volunteers.

ICAM-1 expression was quantified using a sandwich ELISA technique. HLA-DR+ macrophages were identified by immunocytochemical staining. The spontaneous and NAF-triggered release of ROS was determined by a superoxide anion microassay, TNF-α secretion of AM in a standard bioassay using L929 mouse fibroblasts.

In AIDS-patients ICAM-1 levels were elevated (0.84 EU ± 0.25 vs 0.42 EU ± 0.12, $p<0.05$), while the number of HLADR+ AM was slightly decreased (80% ± 5 vs 89% ± 3, $p<0.05$). ICAM-1 levels in the patients group correlated strongly to the NAF-triggered release of ROS ($r$ = 0.7, $p<0.05$) but not to TNF-α secretion. No relation to the absolute numbers of CD4+ lymphocytes and serum levels of p24 could be established.

Highly elevated ICAM-1 levels on AM in AIDS patients lead to increased adhesiveness of those cells, thus enhancing the interaction between AM and lymphocytes and ultimately resulting in a depletion of CD4+ cells in the pulmonary compartment. Although TNF- is a potent inducer of ICAM-1 in AIDS different mechanisms are likely to contribute to the increased expression of ICAM-1 on AM.

### IL-4 and IL-13 exhibit comparative abilities to reduce pyrogen-induced expression of procoagulant activity in endothelial cells and monocytes

*P. Savi, M. Cl Laplace, A. Lalé, F. Dol, A. Dumas, A. Minty,*[1] *D. Caput,*[1] *P. Ferrara*[1] *and J. M. Herbert*

Sanofi Recherche, 195 Route d'Espagne, 31036 Toulouse and [1]Sanofi Elf-Bio recherches, 31676 Labège, France.

Tissue factor (TF) is an ubiquitous membrane-anchored glycoprotein that initiates blood coagulation by forming a complex with circulating factors VII and VIIa. Under normal circumstances, endothelial cells do not express TF activity while they constitutively express thrombomodulin (TM) which accelerates thrombin-catalysed activation of protein C thus contributing to the anticoagulant properties of the endothelium. In some pathological situations, when the endothelium or the monocytes are exposed to inflammatory mediators, they can acquire procoagulant properties.

Endotoxin (LPS), IL-1β and TNFα increased the expression of TF on the surface of cultured bovine aortic (ABAE) or human umbilical vein endothelial cells (HUVEC) and human monocytes while they simultaneously reduced the amount of TM on the endothelial cell surface.

On endothelial cells and monocytes, interleukin-4 (IL-4) and interleukin-13 (IL-13), a newly described cytokine (Nature, 362: 248-50, 1993 ) both strongly inhibited LPS-induced TF expression, a similar activity being also obtained with regard to the effects of IL1 or TNF. Simultaneously, IL-4 and IL-13 counteracted TM down-regulation consecutive to an incubation of endothelial cells with LPS, IL1 or TNF.

These results therefore show that IL-4 and IL-13 both protect the endothelial cell and the monocyte surface against inflammatory mediators-induced procoagulant changes.

### Soluble intercellular adhesion molecule 1 (sICAM-1) in bronchial lavage fluid (BALF) and serum in asthmatics and patients with chronic obstructive bronchitis, smokers, and non-smokers

*[1]G. Rauls, [1]K.-CH Bergmann and [2]U. Rabe*
[1]Allergy and Asthma Clinic Bad Lippspringe, FRG and
[2]Lungenklinik Beelitz, FRG

ICAM-1 may play a central role in airway infiamation. We compared two ELISAs produced by Boehringer Ingelheim Pharmaceuticals (A) and British Bio-Technology Products Ltd (B) in patients with COLD to quantitate the soluble form of ICAM-1. sICAM-1 was detectable by both assays in sera, BALF and saliva and the values of standard curves and samples were closely related. Handling and costs are similar.

Assay A was used to determine sICAM-1 in serum and BALF in patients with bronchial asthma (BA) and chronic obstructive bronchitis (COB), smokers (S) and non-smokers (NS). Values are given in ng/ml.

| Patients | Serum | | | | BALF | | | |
|---|---|---|---|---|---|---|---|---|
| | *n* | *x* | Median | Range | *n* | *x* | Median | Range |
| BA, S | 4 | 237 | 223 | 152–350 | 4 | 13 | 20 | 4–51 |
| BA, NS | 14 | 200 | 206 | 112–276 | 14 | 39 | 30 | 0–141 |
| COB, S | 9 | 278 | 213 | 146–534 | 4 | 13 | 9 | 0–34 |
| COB, NS | 8 | 182 | 178 | 140–248 | 3 | 10 | 10 | 1–21 |

Values given as mean value of double determinations.

Summary: Two assays are commercially available for quantitation of sICAM-1.

Non-smoking asthmatics have similar sICAM values in serum as patients with COB but smoking leads to higher values in both groups. BALF concentrations are higher in asthmatics. Comparison of sICAM-1 values in serum and BALF ($n$ = 14) showed no correlation ($p$>0.5).

### Contrasting effects of endothelins 1, 2 and 3 on vascular permeability in the rat lung.

*S. Lehoux, P. D'Orléans-Juste, P. Sirois and G. E. Plante*

The endothelins are a family of closely related vasoactive peptides. Endothelin (ET)-1 is formed from its precursor via the endothelin converting enzyme (ECE), a phosphoramidon (PA) sensitive metalloprotease. This study was undertaken to verify the effects of ET-1, 2, and 3 and their precursors, big-ET-1,2 and 3, on vascular permeability in the rat lung.

Male Wistar rats received an injection of Evans blue dye (EB), which binds to albumin, in the caudal vein 10 min before being killed. Big-ET-1, ET-1 (400 pmol/kg), big-ET-2 and 3, and ET-2 and 3 (1 nmol/kg) were administered with EB, while PA was injected 5 min earlier. The rats were decapitated and the lungs perfused with 15 ml of Krebs' solution via the pulmonary artery. The trachea (T), upper (UB) and lower bronchi (LB), and parenchyma (P) were removed and tissue content of EB (μg/g dry weight) after treatment was compared to control (*$p$<0.05)

Big-ET-2 and ET-2 did not modify vascular permeability significantly from control values. We conclude that big-ET-1 increases vascular permeability in the lung after its conversion to ET-1 via a PA-sensitive ECE, whereas the decreased EB extravasation by big-ET-3 is unaffected by PA. This suggests that big-ET-1 and big-T-3 are converted to their active moieties through two distinct ECEs. Increased vascular permeability in the lung induced by ET-1 could be involved in the pathophysiology of asthma.

| | EB | Bit-ET-1 | Big-ET-1+PA | ET-1+PA | Big-ET-3 | Big-ET-3+PA | ET-3+PA |
|---|---|---|---|---|---|---|---|
| T | 119±19 | 196±16* | 145±20 | 175±12 | 124±21 | 112±3 | 131±11 |
| BE | 61±8 | 94±13* | 60±4 | 97±9* | 73±13 | 43±4 | 39±2* |
| BI | 54±5 | 84±4* | 54±6 | 117±11* | 32±5* | 40±2* | 45±6 |
| P | 61±3 | 103±7* | 69±2 | 84±5* | 41±6* | 33±3* | 36±4* |

### Endothelin-1 and -3 increase albumin permeability in monolayers of bovine: aortic endothelial cells

*P. Farmer, T. Kawaguchi, M. G. Sirois,*
*P. D'Orléans-Juste and P. Sirois*
Department of Pharmacology, Faculty of Medicine, University of Sherbrooke, Sherbrooke, PQ, Canada, J1H 5N4.

As previous reports have demonstrated that endothelin-1 (ET-1) increases vascular permeability in conscious rats, the present study investigated the direct effects of endothelins on monolayers of bovine aortic endothelial cells (EC). The cells were cultured in medium M199 in the presence of 10% fetal bovine serum (FBS) and antibiotics, seeded on polycarbonate filters fixed in a modified Boyden chamber. Five days after seeding, the medium of the Boyden chamber was replaced with 700 μl of solution which included M199, 0.5% BSA and 3 × $10^6$ cpm of $^{125}$I-albumin. The chambers were placed in a beaker containing 25 ml of M199 and 0.5% BSA. Albumin transfer rate (ATR) through the monolayer was evaluated by the following equation: ATR= $C_0 \times V_0/C_i\Delta t$ where $C_0$= lower chamber concentration, $V_0$ = lower chamber volume and $C_i$ = initial Boyden chamber concentration. $C_0$ was evaluated by withdrawing 75 μl aliquots of medium every 90 s for a total period of 90 min. Endothelins were added to the Boyden chamber after 30 or 45 min. The variation of the permeability was evaluated by linear regression of the control and experimental slopes. ET-1 ($10^{-7}$,$10^{-6}$M) increased the slope value from 0.400 ± 0.017 to 0.900 ± 0.142

μl/min ($n$=4 $p$<0.05) and 0.280 ± 0.003 to 0.804 ± 0.128 μl/min ($n$=5, $p$<0.05) respectively. This represents an increase in permeability of 125 and 187%. ET-3 ($10^{-6}$M) increased the EC permeability in a similar manner: from 0.418 ± 0.013 to 1.13 ± 0.075 μl/min, (n=6, p<0.001; 170%). These results show that ET-I and ET-3 have direct effects on *in vitro* EC permeability and suggest that these phenomena are either mediated by a non-selective receptors for endothelins, or alternatively ET-1 and ET-3 stimulate different receptor types to increase EC permeability. (Supported by the MRC)

### $LTB_4$ increases the permeability of guinea-pig lung epithelial cells to albumin

*T. Kawaguchi, P. Farmer, S. Prie and P. Sirois,*
Department of Pharmacology, Faculty of Medicine, University of Sherbrooke, Sherbrooke, PQ, Canada J1H 5N4.

The effect of $LTB_4$, a potent inflammatory mediator, was studied on the permeability of monolayers of guinea-pig lung epithelial cells to $^{125}$I-labelled albumin. The epithelial cells were collected in Krebs' buffer with protease type XXIV. The isolated cells were suspended in DMEM/FI2 medium containing 10% FBS and seeded (3.0–6.0 × $10^5$ cells/cm$^2$) on fibronectin-treated polycarbonate filters glued at the bottom of modified Boyden chambers. The medium was removed 24 h after seeding and the cells were then maintained in medium without FBS. Thereafter the medium was changed every 48 h. At confluency, 700 μl of medium containing 0.5% bovine serum albumin (BSA) with $^{125}$I-labelled albumin was added to the chambers which were immersed in a beaker containing 25 ml of the same medium without labelled albumin. Aliquots were withdrawn from the beaker at 90 s intervals for 120 min. Albumin permeability was defined as the albumin transfer index: $TI_{Alb}$ = ($C_{t-\Delta t}$ $^{125}$I-Alb.beaker.$V_{t+\Delta t}$ – $C_i$ $^{125}$I-Alb.beaker.$V_t$)/$C_i$ $^{125}$I-Alb.Boydon/Δt. Four groups of cells were studied: 1) a control group (120 min), 2) a group treated with $LTB_4$ ($10^{-6}$M) for 60 min after a control incubation of 60 min, 3) a group that was treated with $LTB_4$ ($10^{-6}$M) after a 10 min preincubation with receptor antagonists, 4) a group treated with $LTB_4$ antagonists only. The $TI_{Alb}$ before and after treatment was compared. The permeability of epithelial cells did not change in the control group. However, the permeability of the cells to albumin was enhanced by 16% ($p$<0.005) following treated with $LTB_4$. Our results suggest that $LTB_4$ could play a role in the airway oedema associated with lung inflammatory and hypersensitivity reaction. (Supported by MRC.)

### Differential muscarinic liberation of $^3$H-arachidonic acid metabolites from epithelium-containing and epithelium-denuded isolated rat tracheae

*G. Brunn, I. Wessler[1] and K. Racke,*
Department of Pharmacology, J.W. Goethe-University of Frankfurt, W-6000 Frankfurt and [1]Department of Pharmacology, University of Mainz, W-6500 Mainz, F.R.G.

Recent experiments from our laboratories showed that prostanoids can modulate neurotransmitter release from isolated rat tracheas.[1] Additional experiments indicated that muscarinic modulation of neurotransmitter release involves indirect, indomethacin-sensitive mechanisms.[2] Therefore, a possible muscarinic liberation of arachidonic acid (AA) metabolites within the airways was tested.

Isolated rat tracheas were labelled with $^3$H-AA and the outflow of tritium was determined. Radioactive compounds in the incubation media were separated by gradient reverse phase HPLC. The spontaneous tritium outflow was 0.13 %/min of the tissue radioactivity. Acetylcholine (ACh) and the muscarinic agonist carbachol concentration-dependently increased tritium outflow maximally by about 30% at 300 and 30 μM, respectively. HPLC separation of the radioactivity showed that ACh and carbachol enhanced several fractions, but the most pronounced effect (three-to four-fold increase) was observed in the fractions representing $^3$H-PGE$_2$ and $^3$H-PGD$_2$. For comparison, the ionophore A 23187 (10 μM) increased the outflow of total tritium by about 100%, but the $^3$H-PGE$_2$/$^3$H-PGD$_2$ fractions about 10-fold. The effect of ACh was antagonized by the M3 selective muscarine receptor antagonist p-fluoro-hexahydrosiladiphenidol (1–10 μM), but not affected by the M2 selective muscarine receptor antagonist methoctramine (up to 10 μM). Removal of the epithelium either before or after the labelling period resulted in a large attenuation of ACh evoked release of total tritium and $^3$H-PGE$_2$/$^3$H-PGD$_2$. In conclusion, activation of muscarine receptors, most likely of the M3 subtype, can cause liberation of AA in the airway epithelium, resulting in an enhanced formation of prostanoids.

### References

1. Racké *et al. Am Rev Respir Dis* 1992; **146**: 1182.
2. Brunn *et al. Naunyn Schmiedeberg's Arch Pharmacol* 1991; **344**: R74.

### Granulocyte macrophage-colony stimulating factor (GM-CSF), interleukin 8 (IL-8) and 15 hydroxyeicosatetraenoic acid (HETE) production by human bronchial epithelial cells in asthmatic and control subjects

*F. Gormand, R. Hosni, S. Cheria, B. Chabannes, M. Lagarde, M. Perrin-Fayolle and Y. Pacheco.*
INSERM U 352, Service de Pneumologie, Centre Hospitalier Lyon-Sud, France.

Human bronchial epithelial cells (HBEC) are involved in bronchial inflammatory processes by secreting cytokines (EL6, IL-8, GM-CSF) and lipidic mediators (Prostaglandins,15 HETE). Biological and cytological abnormalities of bronchial epithelium have been described in asthmatic patients Thus GM-CSF production is increased by HBEC of asthmatic patients.

The aim of this study was to determine the production of GMCSF, IL-8, and 15 HETE by HBEC in control subjects ($n$=6) and asthmatic patients ($n$=3). Human bronchial tissues were obtained from either surgery or biopsy sources. HBEC were isolated from the tissue samples by protease treatment for 24 h at 4°C. Dissociated cells were resuspended in DMEM/HAMFI2 supplemented with growth factors, antibiotics, fetal calf serum (2%) and were plated on to collagen coated dishes at a density of $5 \times 10^3$ cells/cm$^2$. HBEC were stimulated by TNF (100 u/ml) for 0, 3, 12, 24 h and supernatants were collected for analysis. Immunoreactive GM-CSF, IL-8, 15 HETE were quantified by RIA or EIA.

In control subjects, GM-CSF, IL-8, 15 HETE were detected in basal conditions and the levels of cytokines increased with time. Upon TNF stimulation GM-CSF and IL-8 levels increased significantly at 3, 12, 24 h and 12, 24 h respectively. No difference was observed with 15 HETE. In asthmatic patients GM-CSF levels were significantly greater than controls at 24 h . For asthmatic patients TNF did not increase GM-CSF basal levels. In conclusion TNF increased the basal secretion of GM-CSF and IL-8 in control subjects The greater and spontaneous production of GM-CSF in asthmatic patients could be involved in the follow-up of bronchial inflammation in asthma.

### Endothelin and airway cells in asthma

[1]*A. M. Vignola., P. Chanez,* [2]*D. R. Springall, A. Campbell, M. Farce,* [2]*J. Polak. F. B. Michel, J. Bousquet and P. Godard*
Montpellier France, [1]CNR Palermo Italy and [2]London UK.

*Background*: Endothelins represent a family of peptides which might be implicated in asthma because of their potent bronchoconstrictive and proliferative effects. On bronchial epithelial cells endothelin immunoreactivity was upregulated in asthmatics as compared with normal subjects. We studied in asthmatics and normal subjects endothelin immunoreactivity in alveolar macrophages (AM) and bronchial epithelial cells (EC), and, the capability of these cells to release endothelin.

*Methods:* AM were obtained by BAL from 17 asthmatics and nine normal subjects. EC were recovered by brushing (>90% epithelial cells) from nine asthmatics and six normal subjects. Cytospin preparations of AM or EC were stained using a polyclonal antibody against endothelin using the APAAP technique. AM were stimulated with 10 μg/ml of LPS during 24 h in plastic dishes ($n$=26). EC were stimulated for 90 min by A23187 (0.5 μM) for 90 min. The release of endothelin was measured by RIA (Amersham).

*Results:*

| | asthma | controls | $p$ value |
|---|---|---|---|
| immunoreactivity (% positive cells) | | | |
| AM | 8.7 ± 6.5 | 3.2 ± 4.2 | $p$<0.03 |
| EC | 9.5 ± 5.8 | 1.5 ± 2.3 | $p$<0.02 |
| endothelin release (pg/ml) | | | |
| AM resting | 1.3 ± 3.6 | 0.3 ± 0.8 | NS |
| AM + LPS | 5.4 ± 9.6 | 1.8 ± 2.9 | NS |
| EC resting | 1.2 ± 0.3 | 7.4 ± 1.4 | $p$<0.001 |
| EC + A23187 | 8.6 ± 1.4 | 8.0 ± 2.3 | NS |

Statistical analysis by Mann–Whitney $U$-test

*Conclusion:* Endothelin is over-expressed on airway cells in asthma. However, its *in vitro* release, spontaneously or after non-specific stimulation, is not significantly increased in asthmatics.

### Heat stable products from alveolar macrophages of ovalbumin immune and non-immune guinea-pigs that stimulate eosinophils and early progenitors

*P. Elsas, M. I. Elsas, L. A. Silva, D. Joseph, N. Havet, B. Saliou and B. B. Vargaftig,*
Microbiologia UFRJ and FIOCRUZ, Rio de Janeiro, Brazil, Unité de Pharmacologie Cellulaire (U285 I. Pasteur-INSERM), 25 rue du Dr. Roux, 75015, Paris, France.

To define whether products from alveolar macrophages (AM) add to the eosinophilia of asthmatic lung, liquid and semisolid cultures of guinea-pig bone marrow (BM) were seeded in the presence of LPS-stimulated culture supernatants (SN) of 95% pure AM (1% or less T cells). The SN increase eosinophil (EOS) production in liquid culture and support formation of myeloid colonies containing EOS, but not of pure EOS colonies, by acting on purified progenitors devoid of mature EOS. This effect is not duplicated by natural or recombinant sources of GM-CSF which stimulate guinea-pig GM colony formation, and could not be attributed to residual LPS, even though LPS is required for induction of the activity (detectable at 30 min of culture, plateau levels reached at 6 h). The activity is heat resistant (30% residual activity after 30 min heating at 100°C, detected in colony formation assays), with an apparent m.w. of 43 kDa by FPLC on a Superose 8 column, one peak being active on both liquid and semisolid culture. AM SN are active across species barriers, promoting EOS production in liquid culture of human BM and increasing human EOS helminthotoxicity. Comparable levels of activity in liquid BM culture were found in LPS-stimulated AM SN from guinea pigs immunized twice (OVO-OVO) or once (OVO-ALUM) with ovalbumin in alum or from ALUM-ALUM control animals, cultured 48 h after the challenge injection, suggesting that LPS induces maximal secretion irrespective of immunity. Significant activity was found in one out of seven SN from OVO-OVO animals, but in none of 8 OVOALUM and 7 ALUM-ALUM AM SN. While ovalbumin (100 g/ml) induced low levels of activity, these were comparable in AM SN of immune and non-immune animals. The data suggest that a heat stable product of AM contributes to eosinopoiesis and ovalbumin immunization promotes its secretion in the absence of LPS, at least in some animals.

Supported by INSERM-I. Pasteur, CNPq/RHAE, FAPERJ, FINEP and WHO/TDR.

### Phospholipase C and D activation in rabbit alveolar macrophage stimulated by cotton dust tannin or zymozan

*D. Lauque, R. Aslane, M. C. Prevost, H. Chap*
INSERM U326, CHU Purpan, 31059 Toulouse Cedex.

Alveolar macrophages (AM) play a main role in the development of the acute lung inflammation observed after cotton dust inhalation. Tannin, present in large amounts in this respirable dust, promotes arachidonic acid and neutrophil chemotactor factors release by AM. We investigated the role of phospholipase C (PLC) and D (PLD) in the signal-transducting mechanisms of rabbit AM isolated by lung lavage, then stimulated by cotton dust tannin or zymozan. [$^3$H]Lyso-PAF was used to label the phosphatidylcholine (PC) pool of AM. Tannin or zymozan promoted PC hydrolysis, phosphatidic acid (PA) and diglycerides release after several minutes of stimulation. Since these metabolites may come from PC hydrolysis by a PLC or PLD, the exclusive property of PLD of transphosphatidylation in presence of ethanol was used in order to discriminate between these two routes. In presence of tannin and ethanol, phosphatidylethanol (Pet) formation and decrease of PA synthesis were seen. Preinhibition of proteine-kinase C (PKC) by GF 109203X suppressed tannin-induced Pet formation. No Pet synthesis was observed when AM were stimulated by zymozan in the presence of ethanol. We conclude that tannin-induced, but not zymozan-induced, PC, hydrolysis in rabbit AM is, at least partially, mediated through PKC and PLD activation.

### Cytochemical examination of acid phosphatase activity in alveolar macrophages obtained by BAL from patients with pulmonary sarcoidosis

*S. Zunic-Minic, P. Cvetkovic, V. Cemerikic, O. Duric, G. Dordevic-Denic, S. Zunic and D. Mandaric*
University Clinical Center, Clinic for Allergy and Immunology, Belgrade, Yugoslavia

This study was carried out to quantify and compare the presence of acid phosphatase (E.C. 3.1.3.2.) activity in alveolar macrophages (AM) obtained by bronchoalveolar lavage (BAL) from patients with pulmonary sarcoidosis and bronchitis. Thirty-four persons: 15 healthy subjects nine non-smokers and six smokers), nine patients with pulmonary sarcoidosis, (clinical grade I), and 10 patients with different inflamative pulmonary disorders, pathohistologically confirmed as bronchitis and peribronchitis chronic, were lavaged. Activity of alveolitis in sarcoid patients was estimated clinically and supported with the evidence of lymphocytosis and increase of CD4+/CD8+ ratio in BAL. Diagnosis of sarcoidosis was pathohistologically confirmed. The AM from smokers showed slight, but not significant increase of acid phosphatase activity in comparison with healthy non-smokers. Activity of acid phosphatase from sarcoid patients revealed decrease in comparison with the healthy subjects, as well as bronchitis. This finding supports an idea about the use of acid phosphatase activity as a possible non-specific prognostic indicator for the alveolitis in pulmonary sarcoidosis.

### Expression of surface markers on alveolar macrophages (AM) from patients with HIV-infection or sarcoidosis as detected by flow cytometry

*K. Wassermann, M. Subklewe and E. Schell-Frederick*
Med. Dept. I/III, University of Cologne, Joseph Stelzmann Str. 9, D-5000 Cologne 41, Germany.

The goal of this study was to quantify surface antigens on human AM by flow cytometry as an indirect indication of cell function. The primary antibodies were selected to measure monocyte-like cells (Leu M3, Leu M5, My 7, My 9), antigen presenting capacity (HLA-DP, DQ, DR) and the state of cell activation (Transferrin-R, Leu 15, 27El0, RM 3/1, IL-2R, ICAM-1). FITC-labelled F(ab')2 anti-Ig served as a second antibody. A CD2, 3, 19 cocktail was used to gate out lymphocytes. The results are expressed as the median linear fluorescent intensity of the test antibody divided by that of the non-reactive isotype control. This quotient indicates the intensity of specific antigen expression and corrects for differences in autofluorescence between patients. We have analysed cells from 32 HIV-infected patients, ten patients with newly diagnosed sarcoidosis and 13 normal controls. The results show significantly increased intensity of Leu M3, Leu M5, Leu 15, My 9 and HLA-DP expression in both patient groups. There is significantly higher expression of Transferrin-R in AM from patients I with sarcoidosis as compared to controls but no increase in AM from HIV-patients. A comparison of the results in HIV patients with or without Pentamidine aerosol prophylaxis suggests that Pentamidine decreases AM surface marker expression.

### Phagocytosis of viable *Candida albicans* by alveolar macrophages: flow cytometric quantification

*S. Rosseau, W. Seeger, H. Pralle and J. Lohmeyer*
Dept. of Internal Medicine, JLU, Klinikstrasse 36, 6300 Giessen, FRG.

Granulocyte, monocyte and macrophage phagocytic capacity has been assessed by flow cytometric techniques using FITC-labelled heat-killed yeasts and bacteria or BCECF/AM-labelled viable *Staphylococcus aureus* or *Candida albicans*. Application of this approach to AM is hampered or even rendered impossible by the strong autofluorescence of this cell type. This approximates or even surpasses the fluorescence intensity of the labeled bacteria or fungi. Furthermore, heat-killing of microorganisms may result in some overestimation of phagocytosis due to destruction of putative phagocytosis inhibitory factors by this technique. We loaded viable *C. albicans* with the membrane-permeable pH indicator carboxy-SNARF 2/AM, which is cleaved intracellularly to generate the membrane-impermeable derivative carboxy-SNARF 2. Fluorescence was excited with the 488 nm line of an argon-ion laser, with two emission peaks arising at 583 nm and 633 nm. Rabbit alveolar macrophages were labelled using a monoclonal mouse antibody and a secondary FITC-coupled anti-mouse antibody. After coincubation of macrophages and yeasts, paraformaldehyde (PFA, 4%) and 0.5% EDTA in PBS was used to stop the phagocytic process and to detach adherent yeasts from the macrophage surface. This procedure was shown to reduce the adherence-associated background staining to <5%. FITC-labelled alveolar macro-phages were gated for right angle light scatter and green fluorescence, and red fluorescent yeasts were detected with a 630/26 nm bandpass filter. Macrophage-associated yeasts induced a shift from monochromatic (green) to dual (green and red) fluorescence. The percentage of yeast-positive macrophages and the red fluorescence intensity per macrophage were quantified. 85 ± 5% of rabbit alveolar macrophages displayed phagocytic activity within a 60 min incubation period at an 8:1 yeast to macrophage ratio. Yeast opsonization with rabbit serum or rabbit anti-*Candida* IgG was a prerequisite for phagocytosis. Yeast engulfment was completely inhibited in the presence of 0.1 mM *n*-ethyl-maleimide and 10 mM Iodoacetic acid during the incubation period. Conclusion: Phagocytosis of viable *Candida albicans* can be feasibly quantified in alveolar macrophages by flow cytometric techniques designed to circumvene the autofluorescence of this cell population.

### Airway macrophage releasibility during different oestrogenic impregnation

*P. Mourlanette, R. Escamilla, M. C. Prevost, C. Cariven, H. Chap*
Service de Pneumologie et Allergologie, INSERM U 326, Hôpital de Purpan, 31059 Toulouse Cedex.

The sudden appearance or worsening of asthma symptoms in women during menstrual cycles, pregnancy or menopause is widely reported. Moreover, it is suggested that alveolar macrophages (AM) play an important role in physiopathology of human bronchial asthma. The aim of our study is to correlate AM activity in female rats with different oestrogenic blood levels. We investigated four groups of female rats: a control group, three ovariectomized groups, one with 1 oestrogen implant (1I) (corresponding to physiological blood oestrogen level in oestrus), the other with three implants (3I) and the last with an empty oestrogen implant (EI) during 20 days (about duration of four rat menstrual cycles).

We investigated in each group the AM releasability after non-specific stimulation by platelet activating factor (PAF). Under stimulation, [$^{14}$C]arachidonic acid labelled AM showed a decreased release of 20% in 1I group (no progesterone) and of 40% in 3I group (hyperoestrogen and no progesterone) compared to control group ($p$<0.05) and EI groups (no oestrogen and no progesterone) ($p$<0.001). We suggest an oestrogen and progesterone scale dose dependant activation of AM which may play a role in women respiratory failures.

### Allergen-induced skin eosinophilia is inhibited *in vivo* by interferon (IFN) alpha

*E. Hénocq, M. Raffard, M. Biderman-Fabas and B. Bizzini*
Hôpital des Diaconesses, 18 Rue du Sergent Bauchat, 75012, Paris, France

Striking eosinophilic responses, revealed by the Rebuck skin window test, have been observed in patients sensitive to common allergens. The accumulation of eosinophils reaches a peak 18–24 h after the application of the allergen to the denuded skin surface. Recently, it has been demonstrated that IL-4 enhances spontaneous IgE synthesis by peripheral blood mononuclear cells of atopic patients. This IgE synthesis was suppressed dose-dependently by IFNγ or IFNα. Furthermore, a highly significant decrease in the levels of IgE was observed in ragweed sensitive individuals, as compared to controls. In order to determine whether IFNα regulates allergen-induced eosinophil recruitment into the tissues, we studied the effects of human recombinant IFNα on eosinophil infiltration by means of the Rebuck skin window. In ten atopic patients, windows were performed with allergen alone and with allergen admixed with IFNα (50 000 I.U./0.5 ml, intra-dermal). Six patients were allergic to grass pollens, three to D. Pt. and one to candidin. With allergen alone, eosinophil recruitment was of 26.6% of the total cells, versus 4.6% when IFNα was admixed ($p$ = 0.0009). Immediate type wheal and flare reactions were uninfluenced by IFNα ($W$=8.2, $F$=27 versus $W$=9.3 and $F$=28.5). Total IgE were high (800 I.U.) and specific IgE (3.5–41.9 PRU). In three patients (grass pollen allergy), IFNα was injected at three decreasing concentrations, showing a dose-dependent blocking effect at only 50 000 I.U.. One patient with atopic dermatitis had a polysensitivity (grass, cat and food allergy to wheat) and a high IgE level (>6 000 I.U.). Eosinophils represented 40% of the total cell number with grass, versus 5% only with Grass + IFNα respectively and 18% versus 1% with Cat Feld I and Cat Feld I + IFNα respectively. However, the effect of wheat was not inhibited by IFNα suggesting different environmental conditions of allergen exposure and/or insufficient doses of IFNα in those specific conditions. In summary, the skin window assays can be used to study comprehensively the immunoregulatory capacities of cytokines *in vivo*.

### Interaction of PAF with the plasma membrane of eosinophils obtained from allergic asthmatic children

*A. Kantar, N. Oggiano, P. L. Giorgi and R. Fiorini*[1]
Departments of Pediatrics and [1]Biochemistry, University of Ancona, Ancona, Italy.

Platelet activating factor (PAF) is a potent phospholipid mediator involved in a variety of pathophysiological events, including inflammation and asthma. It exhibits a wide range of biological activities on different types of cells including eosinophils. The effect of PAF on the plasma membrane of human eosinophils was investigated by measuring the steady-state fluorescence anisotropy ($r_s$) and fluorescence decay of 1-(4-trimethylammonium-phenyl)6-phenyl-1,3,5-hexatriene (TMA-DPH) incorporated in eosinophils plasma membrane. TMA-DPH $r_s$ value reflects the packing of membrane lipid fatty acid chains, whereas fluorescence decay of TMA-DPH offers a good description of membrane heterogeneity. Eosinophils obtained from seven children with allergic asthma were labelled with TMA-DPH and $r_s$ was measured before and after PAF addition at a final concentration of $10^{-7}$M. The effect of PAF on TMA-DPH fluorescence decay was determined using multifrequency phase fluorometry and data were analysed by a model that assumes a continuous distribution of lifetime values characterized by a Lorentzian shape centred at time C and having a width C. Our results demonstrate that PAF induces a time-limited and significant increase in $r_s$ value, indicating an increase in lipid packing of the membrane. Fluorescence decay measurements show a narrowing in the distribution width of the long component, reflecting a decrease in membrane heterogeneity after the addition of PAF. These changes were blocked in the presence of the PAF antagonist, L-659,989.

### The metabolism of arachidonic acid by the guinea-pig tracheal epithelial cells is selectively modulated by eosinophils

*P. Sirois and S. Prié*
Department of Pharmacology, Faculty of Medecine, University of Sherbrooke, Sherbrooke, P.Q., Canada, JlH 5N4.

Airway epithelial cells are not only responsible for the mucociliary clearance of the airways but are also involved in the control of airway reactivity and tone by the release of a number of myotropic factors. Experimental evidence suggests that selected functions of these cells are modulated by inflammatory cells such as eosinophils, lymphocytes and neutrophils. The aim of the present study was to evaluate the influence of eosinophils on prostaglandin $E_2$ ($PGE_2$) and thromboxane $B_2$ ($TxB_2$) synthesis by the guinea-pig tracheal epithelial cells (tracheocytes). Guinea-pig tracheocytes were isolated by enzymatic treatment with 0.1% protease XXIV at 37°C for 1 h. Freshly isolated tracheocytes were cultured in a serum-free medium (DMEM/F12). Eosinophils were isolated by bronchoalveolar lavage, 24 h after i.v. Sephadex bead injection (G50, 24 mg/kg). The purification of eosinophils was performed on a continuous Percoll gradient (65%). Our results showed that unstimulated tracheocytes produced eight times more $PGE_2$ (176 ± 62 pg/ml ) than $TxB_2$ (21 ± 5 pg/ml). In contrast, eosinophils synthesized mostly $TxB_2$ (9300 ± 1850 pg/ml) and 26-fold less $PGE_2$ (360 ± 97 pg/ml) over a period of 2 h. In the presence of arachidonic acid (0.1–10 μM), the synthesis of $PGE_2$ and $TxB_2$ by tracheocytes increased up to 450-fold and 28-fold respectively. Eosinophils incubated in the presence of arachidonic acid released 22 and two times more $PGE_2$ and $TxB_2$ respectively. In coculture, the basal release of $PGE_2$ and $TxB_2$ was 163 ± 12 pg/ml and 430 ± 96 pg/ml respectively. In presence of arachidonic acid, the synthesis of $PGE_2$ and $TxB_2$ by tracheocytes cocultured with eosinophils increased up to 17- and 28-fold respectively. In brief, the presence of eosinophils strongly reduced the formation of $PGE_2$ but not that of $TxB_2$ from tracheocytes incubated with exogenous arachidonic acid. Similar results were obtained when tracheocytes were incubated with major basic protein (MBP; 100 μg/ml ) in presence of arachidonic acid (10 μM); the synthesis of $PGE_2$ was decreased whereas the synthesis of $TxB_2$ was not affected. These results suggest that eosinophils may release MBP which decreases the synthesis of $PGE_2$ by tracheocytes, without affecting synthesis of $TxB_2$. (Supported by the MRC.)

### Bronchial hyperreactivity and pulmonary eosinophilia in guinea-pigs

*P. Sirois and K. Maghni*
Département de pharmacologie, Faculté de médecine, Université de Sherbrooke, Sherbrooke P.Q., Canada.

The characteristic features of bronchial asthma are the bronchoconstriction, airway inflammation characterized by oedema, leukocyte migration and epithelial desquamation, and bronchial hyperreactivity. We have recently developed a model to study bronchial hyperreactivity and pulmonary eosinophilia by the intravenous injection of Sephadex beads (24 mg/kg) to conscious guinea-pigs. Significant cell migration was noted in the airways 24 h after the injection of Sephadex beads. Cell counts in bronchoalveolar lavage increased from $20 \times 10^6$ to $85 \times 10^6$. Guinea-pig bronchi of Sephadex treated guinea-pigs were hyperreactive to acetylcholine and histamine as evaluated *in vitro*. Pretreatment of the animals with aspirin (30 mg/kg) reduced by 43% the eosinophil infiltration but increased by 10–19% the bronchial hyperreactivity to histamine and acetylcholine. Compound MK866 (5 mg/kg) reduced the eosinophil infiltration by 49% and the hyperreactivity by 5–22%. Compound BW755c (15 mg/kg) reduced the eosinophil infiltration by 56% and the hyperreactivity by 8%. The PAF antagonist BN52021 (20 mg/kg) decreased the eosinophil infiltration by 41% and the hyperreactivity by 16–28%. Dexamethasone (5 mg/kg) decreased the eosinophil infiltration by 43% and increased the hyperactivity by 12–16%. Ketotifen (15 mg/kg) reduced the eosinophil infiltration by 66%, but increased the hyperactivity to acetylcholine by 68%. From these results, it does not appear that eosinophil infiltration is always correlated with bronchial hyperactivity. It is suggested that other cell types could also play a major role in this complex interaction. (Supported by the MRC.)

### New model of bronchial hyperreactivity and airway eosinophilia in guinea-pigs

*K. Maghni, C. Lanoue, S. Cloutier, J-P. Cristol, F. Blanchette, A. Cadieux and P. Sirois*
Department of Pharmacology/Faculty of Medicine, University of Sherbrooke, Sherbrooke P.Q., JIH 5N4 Canada.

Bronchial asthma is mainly characterized by airway inflammation and bronchial hyperreactivity (BHR). Experimental evidence suggests that eosinophils (EO) are involved in the pathogenesis of asthma. However, in some experimental models of asthma the BHR did not seem related to EO infiltration in alveolar walls and in some lung diseases, pulmonary eosinophilia was observed whereas some subjects do not present BHR to methacholine. Recently, we developed a new model of airway eosinophilia (AE) in guinea-pigs following intravenous injection of G-50 Sephadex beads (24 mg/kg). The AE was assessed by bronchoalveolar lavage (BAL). The BHR to histamine (His) and acetylcholine (Ac) was measured *in vitro* (bioassays) on airway tissue preparations. Histological studies were also performed. The injection of Sephadex beads induced a marked AE (around $36 \times 10^6$ EO recovered in BAL fluid) in guinea-pigs 1 day post-injection (p.i.). The number of EO decreased to 22% and 41% 7 and 14 days p.i.. The lower bronchi of Sephadex-treated animals presented a BHR to His with an increase of the maximal tension (Emax) of 4.6-, 2.6- and 3.2-fold at 1, 7 and 14 days respectively. BHR to Ac was also observed with a 4.8-, 2.3- and 2.6-fold increase of Emax 1, 7 and 14 days p.i., respectively. In the pulmonary parenchyma, there was no BHR to His and Ac 1 day p.l. whereas a significant increase of Emax was observed for the two agonists 7 and 14 days p.i. In Sephadex-treated animals an important infiltration of EO in the epithelium of the lower bronchi was noted in histological studies. A marked infiltration of EO was also found in the pulmonary parenchyma but few (if any) EO were seen in the bronchiolar epithelium 1 day p.i. However, 7 and 14 days p.i., a marked increase in the number of EO was observed in the epithelium. In conclusion, the Sephadex-induced BHR in guinea-pigs seems to be related to the degree of EO infiltration in the airway epithelium rather than to the presence of EO into the alveolar walls. Bronchial epithelium desquamation was not apparent. These results suggest that the airway eosinophils could reduce or inhibit the release of relaxing factors by bronchial epithelial cells according to mechanisms not yet understood. (Supported by the MRC.)

### Correlation of bronchoalveolar cell influx, cell activation and bronchial responsiveness after chronic inhalation of ovalbumin (OA) in sensitized guinea-pigs

*H. O. Heuer, B. Wenz, H. M. Jennewein, K. Urich*
Department of Pharmacology, Boehringer Ingelheim KG, W-6507 Ingelheim, and Faculty of Biology, Johannes Gutenberg University of Mainz, W-6500 Mainz

The aim of the following study was to examine the association between inflammatory cell influx, cell activation status and change of bronchial responsiveness to acetylcholine (ACh) after daily inhalation of ovalbumin (OA) in sensitized guinea-pigs (GPs). *Materials and Methods:* (a) Starting 3 weeks after sham (S-control; saline) or OA-sensitization (OA at 50 mg/kg s.c. + i.p.) GPs were inhaled daily with 2% OA (10 min; under cover of 0.5 mg/kg mepyramine i.p. 15 min before OA) or saline (S-control) for 2 weeks. (b) Concentration–response curves (CRCs) for inhaled ACh were performed 24 h after the last OA-challenge and 24 h after another single OA-inhalation 1 week later. (c) Bronchoalveolar lavage (BAL) with analysis of inflammatory cell numbers ($\times 10^6$/lung) and their activation status [eosinophil peroxidase, EPO ($\times$ absorption units $/10^7 eos^* ml^{-1}$); myeloperoxidase, MPO ($\times 10^{-6}$U MPO/PMN)] was performed after the last CRC. (d) Two treatment groups of OA sensitized/inhaled GPs received either methyl-prednisolone (NT group; 40 mg/kg i.p. 24 h before first and very last and 1 h before each OA challenge) or paf-antagonist WEB 2347 (WEB; 10 mg/kg i.p. 1 h before each challenge) ($n$=7–10/group). *Results* (see Table;*units in text): (1) BAL from repeatedly OA-sensitized/challenged GPs showed a significant increase of total cell count by about 10-fold and increases in eosinophils (eos) by about 20-fold, neutrophils (PMNs) by 30-fold, macrophages (macros) by about five-fold and lymphocytes (lymphs) by about 10-fold ($p<0.05$, multiple Wilcoxon-test). (2) In contrast, markers of cell activation (EPO, MPO) were significantly decreased ($p<0.05$). (3) MP almost completely and the paf-antagonist WEB 2347 partially reversed these changes in increased cell numbers and decreased cell activation (vs. OA contr., $p<0.05$). (4) CRCs for inhaled ACh were neither affected 24 h after the last OA challenge (daily for 2 weeks) nor 24 h after another OA-inhalation 1 week later.

| | S-contr. | OA-contr. | OA/MP | OA/WEB |
|---|---|---|---|---|
| total cells | 16.2±0.8 | 164.1±20.8 | 24.8±3.9 | 110.6±14.9 |
| eos | 4.9±0.6 | 98.7±15.8 | 7.1±1.5 | 66.0±10,7 |
| PMNs | 0.46±0.23 | 15.0±2.9 | 1.2±0.4 | 9.9±3.0 |
| macros | 8.9±0.3 | 46.4±4.8 | 14.5±2.2 | 31.6±2.7 |
| lymphs | 0.2±0.03 | 1.9±0.7 | 0.35±0.09 | 0.62±0.27 |
| EPO | 7.8±2.0 | 0.64±0.09 | 5.0±1.1 | 1.4±0.1 |
| MPO | 39.5±8.1 | 0.61±0.1 | 97.9±60.5 | 2.68±0.6 |

In conclusion the dissociation between the massive inflammatory cell influx into the BAL on the one hand and decreased activation status of these (probably previously activated. but presently exhausted) cells on the other hand might explain the absence of bronchial hyperresponsiveness after chronic antigen-exposure in this model.

### Effects of CS-518, a thromboxane synthase inhibitor, on eosinophil accumulation and activation

*K. Itoh, O. Mukaiyama, E. Takahashi, Y. Satoh and T. Yamaguchi*
Biological Research Laboratories, Sankyo Co., Ltd., 1-2-58 Hiromachi, Shinagawa, 140 Tokyo, Japan.

The effects of CS-518, a thromboxane $A_2$ ($TXA_2$) synthase inhibitor, on eosinophil accumulation and activation were investigated in an experimental asthmatic guinea-pig model and in several cellular models, such as $TXA_2$ production, eosinophil peroxidase (EPO) release, cell adherence to serum coated plates and chemotaxis measured by modified Boyden method. In the *in vivo* studies, CS-518 inhibited eosinophil accumulation and EPO release into the bronchoalveolar lavage fluid (BALF) of the late asthmatic guinea-pig model. This compound also inhibited PAF, $LTD_4$ and IL-5-induced eosinophil accumulation and $LTD_4$-

induced release of eosinophil chemotactic factor into BALF. In the *in vitro* studies, CS-518 more markedly suppressed $TXA_2$ production, EPO release and cell adherence than chemotaxis. This compound potentiated production of prostaglandin $I_2$ ($PGI_2$), which inhibited chemotaxis and EPO release from guinea-pig eosinophils. The present studies provide further support that $TXA_2$ and $PGI_2$ are involved in modulation of the eosinophil functions and indicate that CS-518 is a potent inhibitor of eosinophil activation.

### Models of bronchial allergic inflammation induced by ovalbumin in guinea-pig, rat and mouse

*J. P. Tarayre, M. Aliaga, M. Barbara, N. Malfetes*
Centre de Recherche Pierre Fabre, 17 avenue Jean-Moulin, 81100 Castres, France.

Various *in vivo* models of bronchial allergic inflammation have been induced in guinea-pigs, rats and mice after sensitization and aerosol challenge with ovalbumin. In guinea-pigs actively sensitized there was an increase, generally maximum 24–48 h after challenge, in the number of eosinophils, neutrophils and mononuclears (with quantitative differences according to the mode of sensitization) in bronchoalveolar lavage fluid (BALF). After sensitization with complete Freund's adjuvant, the number of airways epithelial cells was also increased at 24–48 h. Tle prevention of the symptoms of anaphylactic shock by pretreatment with mepyramine did not modify the spectrum of inflammatory cells in BALF at 24 h. The allergic reaction induced in Brown Norway (BN) and Sprague–Dawley rats actively sensitized with ovalbumin and *Bordetella pertussis* suspension was characterized by an increase in eosinophils and neutrophils in BALF maximum also at 24–48 h. In guinea-pigs and rats, passive sensitization and challenge induced no bronchial inflammation (rat) or an inflammation inferior to that obtained after active sensitization (guinea-pig). In Swiss mice actively sensitized with sc ovalbumin in incomplete Freund adjuvant we did not obtain a bronchial inflammation superior to the non-specific increase in neutrophils in non-sensitized animals. Drugs administered 5 min and 5 h after challenge have been experimented on the 24-h inflammation in guinea-pigs and BN rats. Dexamethasone acetate only reduced the number of eosinophils in guinea-pigs and in rats its effects were superior on eosinophils than on neutrophils. Cromoglycate invariably decreased the eosinophils; its action on the other leucocytes was different according to the model. Theophylline always reduced the accumulation of eosinophils with activity on mononuclears in guinea-pigs and neutrophils in rats. Cetirizine, ketotifen, and salbutamol decreased eosinophils only in rats. Mepyramine and indomethacin were inactive. Some drugs have been experimented only in BN rats while cyclosporine A, methotrexate, rolipram and furosemide were active on eosinophils and neutrophils, chloroquine, aurothiopropanol sulphonate and pinacidil decreased only eosinophils; methysergide and atropine were inactive.

### Dissociation between airway eosinophilia and bronchial responsiveness in IL-5 transgenic mice

*J. Lefort, D. Leduc, C. M. Bachelet and B. B. Vargaftig*
Unité de Pharmacologie Cellulaire, Unité Associée
Institut Pasteur-INSERM U285, 25, rue du Docteur Roux, 75015, Paris, France.

To determine if bronchopulmonary hyperresponsiveness and eosinophil recruitment into the airways are associated in mice, lung inflammation (histology and counts in bronchoalveolar lavage fluid, BALF) was correlated to bronchoconstriction by 10–320 µg/kg of 5HT or methacholine i.v. in Swiss and IL-5 transgenic mice (donated by Drs. C.J. Sanderson and C.M. Hetherington; see Dent et al., J. Exp. Med., 172, 1425, 1990). After immunization on days 0 and 7 with sc ovalbumin (100 g in 1.6 mg of aluminium hydroxide), 1–100 µg OA were instilled into both nostrils on day 14. Naïve, sham immunized and sham challenged Swiss mice contained no eosinophils in their airways nor in the BALF, provocation being followed at 24 and 96 h (but not 6 h) by a dose-dependent increase up to 35% of eosinophils in the BALF, which was suppressed by dexamethasone (2.5–5.0 mg/kg) and returned to basal values in 14 days. Lungs had a normal microscopic aspect up to 2 h after challenge, and were inflamed 6 h to 7 days thereafter, with eosinophils localized essentially around central airways and blood vessels. IL-5 transgenics had eosinophils in lungs and blood, but not in the BALF; saline-challenged immunized animals had approximately 10%, and OA-challenged animals had 40–65%, eosinophils in the BALF. After anaesthesia (i.p. urethane, 45 mg/10 g and pancuronium, 0.25 mg/kg), the trachea was cannulated and lung resistance and compliance were measured with a Mumed PR800 system. The threshold for bronchoconstriction, peak effects and PD20, PD60 and PD90 (doses which increased the responses by 20, 60 and 90%, respectively) were not significantly modified 24 or 96 h after antigen challenge in both Swiss and IL-5 mice, with a tendency for augmented responses at 3 h, quite before eosinophil recruitment into the BALF. Eosinophil recruitment alone does not cause hyperresponsiveness, their further activation being probably required. Alternatively, early signs of hyperresponsiveness, which will be further studied in appropriate mice strains, suggest that hyperresponsiveness may precede overt eosinophil infiltration.

### The effects on andolast (CR 2039) on Sephadex-induced eosinophilia and lung hyperresponsiveness in the rat

*F. Ferrari, L. Revel, O. Letari and F. Makovec*
Rotta Research Laboratorium, Via Valosa di Sopra 7, 20052 Monza, Milano, Italy.

Lung eosinophilia and hyperreactivity are characteristic of chronic asthma. We induced eosinophilia and hyperreactivity in the lungs of rats by intravenous injection of Sephadex particles, according to the method of Spicer *et al.*[1] and Cashin *et al.*[2] Following single injection of

Sephadex G100 or repeated injections of Sephadex G200, we observed an increase in number of eosinophils and a fall in number of mononuclear cells in the bronchoalveolar lavage (BAL) fluids of the rats. Moreover, the animals were hyperresponsive to the bronchoconstriction induced by acetylcholine (Ach) *in vivo* and to the spasmogenic effect of 5-hydroxytryptamine (5-HT) *in vitro*. In this context, we examined the effect of andolast (4-(lH-tetrazol-5-yl)-N-[4-(lH-tetrazol-5-yl] phenylbenzamide), a putative new antiallergic compound[3] in comparison with disodium cromoglycate (DSCG). Andolast administered i.m. or s.c. in a dose range of 3–30 mg/kg reduced the increase in number of eosinophils in the BAL fluids of rats treated with Sephadex i.v. Besides, at the same doses, it reduced hyperreactivity to 5-HT *in vitro* and the hyperresponsiveness to Ach *in vivo*. DSCG was ineffective up to 100 mg/kg i.m. or s.c. in reducing lung eosinophilia. Hyperreactivity to 5-HT *in vitro* and hyperresponsiveness to Ach *in vivo* were also not reduced by DSCG.

**References**

1. B.A.Spicer, R.C. Baker, P.A. Hatt, S.M. Laycock and N. Smith, *Brit. J. Pharmacol.* (1990), **101**, 821–828.
2. C.H. Cashin, P.J. Dunford, P. Newbold and H.J. Wood, *New Drugs in Allergy and Asthma*, Davos (CH) Congress, July 1992.
3. Revel L., Colombo C., Ferrari F., Folco G.C., Rovati L.C. and Makovec F., *Eur. J. Pharmacol.* (1992), **229**, 45–55.

### Cytokine secretion by monocytes in patients with primary immunodeficent state

*F. Abdullayev, A. Umnyashkin and O. Abdullayev*
Quarter 2514, Zar dabi Street, Baku 370118, Azerbaijan Republic, Institute of Phtisiology and Pulmonology.

Monocytes and macrophages play an important role in the cellular immune response of inflammation and have immunoregulatory properties by production and secretion of cytokines in patients with primary immunodeficient state (PIS). We have measured the production of interleukin 1 (IL-1), interleukin 6 (IL-6), and tumour necrosis factor (TNF) by unstimulated monocytes and monocytes stimulated with lipopolysaccharide isolated from the peripheral blood of patients with hereditary glucose-6-phosphate dehydrogenase deficiency (G6PD), which apply to PIS (89 persons) and healthy control (70 persons). Spontaneous and lipopolysaccharide-induced cytokine production were not significantly different between patients and control. Median lipopolysacharide-stimulated cytokine secretion for patients and control was 1.55 and 4.29 U/ml for IL-1, 469 and 630 U/ml for IL-6, and 458 and 592 pg/ml for TNF. It has been speculated that some clinical signs of PIS such as fever and cachexia could be ascribed to the action of cytokines as IL-1 or TNF. In addition, the high level of immunoglobulins observed in the course of the PIS have been ascribed to increased secretion of B cell differentiation factors such as IL-6 induced by PIS.

### Defensin-induced response of peripheral blood monocytes in bronchial asthma: a new diagnostic test

*A. Kubatiev, O. Fesenko, S. Tkachenko and E. Gembitski*
Department of General Pathology and Pathophysiology, National Center of Postgraduate Medical Training, 2 Barrikadnaya Street 123836 Moscow, Russia.

Defensin (D) is referred to the family of non-fermentative cationic peptides with a broad spectrum of microbicidal, antiviral and cytotoxic activity. In experiments *in vitro* D of human neutrophils (1 μg/ml–100 μg/ml) causes a distinct dose-dependent aggregation of donors monocytes (M) intensifying under the influence of arachidonic acid (AA) 40 μM and phorbol myristate acetate (PMA) 10 nM. Additional stimulation of donor's M by D exhibits no changes of their aggregational degree. However, in patients with bronchial asthma D exerts no essential influence on M aggregational activity stimulated by AA and PMA. It appears to be accounted for by non-ability of M activated by D present in patient's plasma to react to additional D stimulation *in vitro*. Thus investigation data permit to regard D-induced aggregation of M as a new sensitive test for estimating their functional condition in patients with bronchial asthma.

### Elafin, a potent inhibitor of human leukocyte elastase

*O. Wiedow, J.-M. Schröder and E. Christophers*
Dept. of Dermatology, University of Kiel, Schittenhelmstr.7, D-W-2300 Kiel, Germany.

In acute and chronic inflammation leukocyte infiltration and tissue damage are the major events. In particular in lung emphysema the inflammatory infiltrate is dominated by neutrophils and macrophages. Both cells have potent serine proteases, like human leukocyte elastase, proteinase 3, and cathepsin G, which are able to degrade a wide variety of matrix proteins. Since human leukocyte elastase and proteinase 3 are able to produce emphysema-like lung destruction in animals, inhibitors of these enzymes are suspected to have therapeutical effects in inflammatory lung diseases like emphysema.

Elafin, a potent inhibitor of human leukocyte elastase (app. Ki: 6 x $10^{-10}$M) and proteinase 3, had first been purified from horny layers of patients suffering from psoriasis. This inhibitor is able to prevent the metabolism of insoluble matrix proteins like elastin or keratin by elastases. From investigations of Sallenave *et al.* (*Biol Chem Hoppe-Seyler* 1992; **373**: 27.) It became clear that this inhibitor is also present in bronchial mucus. Elafin is an acid and alkaline stable 57 amino acid peptide. We could now isolate several elafins with N-terminal deletions (up to 5 AA) and elongations (up to 19 AA) from human skin with the same inhibitory activities. The N-terminus of the elongated molecules contains transglutamination sites, which make it likely that the native molecule is coupled to matrix proteins. Therefore, elafin seems to be an important antiinflammatory compound *in vivo*. Since recombinant elafin is now available, its potential for therapeutic use in several diseases with neutrophil-mediated tissue damage has to be investigated.

### Neurotransmitters modify the responsiveness of stimulated neutrophils

[1]M. Gajewski, [2]H. Laskowska-Bozek, [1]S. Maslinski and J. Ryzewski[2]
[1]Departments of Biochemistry and [2]Pathophysiology, Institute of Rheumatology, Warsaw, Poland

Reactive oxygen species (ROS) have attracted increasing attention for their possible role in promoting inflammation. However, ROS metabolism of phagocytic cells may be substantially modified by neurotransmitters of the autonomic system. Human peripheral neutrophils were studied to assess the effect of adrenergic and muscarinic cholinergic agonists (adrenaline and carbachol, respectively) on neutrophils luminol-enhanced chemiluminescence (CL). Both agonists were ineffective when resting neutrophils were tested. We confirmed that adrenaline inhibited CL evoked in activated neutrophils. An unexpected further increase in CL of zymosan-activated neutrophils was observed following carbachol treatment. This increase correlated with effects of cholinergic agents on neutrophil lysosomal enzyme release. Both effects were prevented by 1 μM atropine. The increase in the luminol-enhanced CL seems to correlate with the expression of muscarinic receptors on neutrophils. We conclude that the opposite effects of adrenergic and muscarinic receptor agonists on the activity of neutrophils may reflect their opposing influences on inflammation, and may support the idea of 'proinflammatory' effect of parasympathetic system.

### 5-lipoxygenase-activating protein binds arachidonate and increases its utilization by 5-lipoxygenase

*P. Vickers, J. Mancini, E. Wong, S. Charleson, M. Cox, and M. Abramovitz*
Merck Frosst Centre for Therapeutic Research, P.O. Box 1005, Pointe Claire-Dorval, Quebec H9R 4P8, Canada.

5-Lipoxygenase (5-LO) and 5-lipoxygenase-activating protein (FLAP) are both essential for cellular leukotriene (LT) synthesis and represent alternative targets for LT biosynthesis inhibitors. However, the mechanism by which FLAP activates 5-LO has remained unclear. We have expressed human FLAP and human 5-LO to high levels, both alone and in combination, in *Spodoptera frugiperda* (Sf9) insect cells using recombinant baculoviruses. As 5-LO synthesizes $LTA_4$ and 5-HPETE in intact Sf9 cells in response to exogenous arachidonic acid (AA) and calcium ionophore A23187, analysis of 5-LO activity in Sf9 cells in the absence or presence of FLAP provides a system to study the specific effects of FLAP upon cellular 5-LO activity. In this system, FLAP increased the total amount of $LTA_4$ and 5-HPETE synthesized from AA, allowed 5-LO activity at low AA concentrations and increased the $LTA_4$:5-HPETE product ratio relative to cells expressing 5-LO alone. These effects of FLAP upon 5-LO activity were inhibited in a concentration-dependent manner by MK-886, an LT biosynthesis inhibitor which specifically binds to FLAP. We have also demonstrated that FLAP, both in human leukocytes and expressed in Sf9 cells, specifically binds [$^{125}$I]-L-739,059, a photoaffinity analogue of AA, with this binding being inhibited both by unlabelled AA ($IC_{50}$ of 10–20 mM) and MK-886, but not by analogues of MK-886 which are not effective as inhibitors. The demonstration that FLAP specifically binds AA and increases the utilization of this fatty acid suggests that FLAP may function as an arachidonate transfer protein for 5-LO.

### Ammonium chloride decreases cell surface IL-8 receptor expression and chemotactic responsiveness of neutrophils (PMN)

*J. K. Shute, J. E. Parry, M. C. A. Clarke, J. O. Warner, M. K. Church and T. P. Dean*
Immunopharmacology and Child Health Groups, Southampton General Hospital, Southampton S09 4XY, UK.

Neutrophils are the primary target of IL-8, a chemokine associated with several inflammatory conditions, such as psoriasis, rheumatoid arthritis and, as we have recently shown, cystic fibrosis. In this study, we compared the effects of red blood cell (RBC) lysis using isotonic $NH_4Cl$ or hypotonic NaCl on the subsequent determination of cell surface IL-8 receptors and chemotactic responsiveness of PMN. PMN were purified from peripheral blood of normal and atopic individuals by dextran sedimentation of RBC followed by removal of mononuclear cells on Lymphoprep. RBC contaminating the granulocyte pellet were lysed in 0.15 M $NH_4Cl$ for 15 min at 4°C or 0.2% NaCl for 45 s at 20°C. Scatchard analysis of the binding of radioiodinated IL-8 to granulocytes revealed no significant difference between normal ($n$=5) and atopic ($n$=4) donors when cells were prepared by either method. However, both the number and the $K_D$ of IL-8 receptors were significantly ($p$ <0.001) reduced when $NH_4Cl$ was used to lyse RBC compared with NaCl ($n$=9). The number of receptors on cells exposed to $NH_4Cl$ was less than 35% of that measured on the same cells exposed to hypotonic NaCl. Correspondingly, the *in vitro* chemotactic response of PMN to an optimum concentration of IL-8 was significantly ($p$ <0.01) reduced by 40% ($n$=10). $NH_4Cl$ is a weak base that raises intralysosomal pH, interfering with recycling of receptors to the cell surface following binding of ligand and internalization. We conclude that the use of $NH_4Cl$ should be avoided in the preparation of granulocytes for investigation of IL-8 responsiveness and quantification of surface receptors.

### Effect of chronic polymyxin B and ovalbumin (OA) inhalation on bronchoalveolar lavage (BAL) cell influx and cell activation status in sensitized guinea-pigs: massive inflammatory cell infux contrasts to bronchial hyporesponsiveness after polymyxin B

*H. O. Heuer, B. Wenz, [1]R. H. Gundel, H. M. Jennewein and K. Urich*

Dept. of Pharmacology, Boehringer Ingelheim KG, W-6507 lngleheim/Rhein, [1]Boehringer Ingleheim Pharmaceuticals Inc., Ridgefield, CT, USA and Faculty of Biology, Johannes Gutenberg University of Mainz, W-6500 Mainz (FRG).

The following study was aimed at studying the effect of chronic/weekly polymyxin B inhalation on bronchoalveolar cell influx and activation status, their interaction with intermittent ovalbumin (OA) inhalation and bronchial responsiveness in sensitized guinea-pigs (GPs). *Methods:* (a) GPs were allocated to three groups (I, II, III). GPs of group I and II were sensitized three times (week 3, 5, 20) with 10, 5 and 2 g ovalbumin (OA), respectively, in 10 mg $Al(OH)_3$/GP. A treatment with cyclophosphamid at 30 mg/kg i.p. preceded each sensitization 2 days before. Weekly inhalations of polymyxin B were performed for maximal recruitment and activation of inflammatory cells, starting 2 weeks before (week 1) first sensitization and continued throughout the study period. In difference to group I, group II was exposed to additional and intermittent (up to nine times) OA challenges. Group III received sham-sensitization and sham-inhalations. (b) During weeks 30–31 bronchial responsiveness to inhaled acetylcholine (ACh) was determined 24 h after OA (1% for 5 min under cover of 10 mg/kg i.p. mepyramine, 15 min before OA; group I) and compared to sham-controls (group III). (c) Bronchoalveolar lavage (BAL) was performed 24 h after another OA-/saline-challenge during weeks 38–40 in all three groups and analysed for cell numbers and status of cell activation (eosinophil peroxidase, EPO; myeloperoxidase, MPO). *Results:* (1) 24 h after OA-challenge in multiple polymyxin B inhaled GPs (group 1) a significantly reduced responsiveness to inhaled ACh occurred (hyporesponsiveness!) compared to sham sensitized/sham challenged GPs (Group III) (median $EC_{50}$: group I 0.072 mg/ml ACh, $n$=13; group III 0.025 mg/ml ACh, $n$=14; $p$<0.05). (2) Repeated and weekly inhalation of polymyxin B (without intermittent OA-challenge; group I) led to an unspecific increase of all inflammatory cells (x$10^6$/lung) including eosinophils (eos), neutrophils (PMNs) macrophages (macros), lymphocytes (lymphs) (Group 1, polymyxin B: total 88.1 ± 10.1, eos 19.7 ± 4.7, PMNs 21.1 ± 5.5, macros 42.8 ± .2, lymph 2.8 ± 1.4, $n$=6; vs group III, sham: total 26.9 ± 2.5, eos 3.8 ± 0.7, PMNs 4.2 ± 1.7, macros 17.8 ± 1.7, lymphs 0.7 ± 0.2, $n$=14; $p$<0.05). (3) In contrast, repeated and weekly inhalation of polymyxin B combined with intermittent OA-challenge (group II) led to a preferential and significant influx of eos (group II vs. III; $p$<0.01). Neutrophils, macrophages and lymphocytes were not affected in a relevant manner ($p$>0.05) (group II, polymyxin B + intermittent OA: total 50.2 ± 14.7, eos 20.2 ± 7.4, PMNs 7.7 ± 3.1, macros 20.1 ± 3.9, lymphs 1.1 ± 0.2). (4) EPO-content per eos × absorption units/$10^7$ eos × $ml^{-1}$) as a marker for eos activation was significantly decreased in polymyxin B inhaled GPs (median group I vs. III: 2.5 vs 10.1, $p$<0.05). MPO per PMN (× $10^{-6}$U MPO/PMN) was not affected significantly between groups (median group I vs. III; 1.7 vs. 6.4, $p$>0.05). *Conclusions* (1) Massive inflammatory cell influx may not necessarily result in bronchial, hyperresponsiveness. (2) In contrast, decreased activation status of eo may explain the observed significant bronchial hyporesponsiveness after chronic polymyxin B inhalation. (3) The findings are still consistent with a modulatory role of eos in regulation of bronchial responsiveness.

### Inhibition by rapamycin of LPS-induced leukocyte infiltration in guinea-pig airways

*J. Francischi*, D. M. Conroy and P. Sirois*

Department of Pharmacology, Faculty of Medicine, University of Sherbrooke, PQ, Canada

The effect of rapamycin, a new immunosuppressive drug (Morris, 1992) was studied on LPS-induced cell infiltration in guinea-pig lung. Twenty-four hours following the intratracheal injection (i.t.) of LPS (*E. coli* 0111:B4, 8 μg/kg) to anaesthetized guinea-pigs (Dunkin-Hardey, 300–350 g) there was a significant increase in leukocyte number in blood (2933 ± 452 to 4667 ± 417 cells/$mm^3$) and bronchoalveolar lavage fluid (BAL; 20 ± 5 × $10^6$ to 117 ± 20 × $10^6$, $n$=6) when compared to an i.t. injection of apyrogenic saline. The increase in leukocyte number in BAL was related to a marked neutrophil influx which comprised approximately 60% of the total cells. A two-fold increase in the maximal contraction of superfused strips of bronchial tissues to histamine and acetylcholine (ACh) was observed 24 h following LPS injection although this increase in bronchial reactivity was not statistically significant. Rapamycin (5 m/kg) administered intramuscularly 2 h before LPS injection significantly reduced the leukocyte count in blood (3210 ± 165 cells/$mm^3$) and BAL (32 ± 6 × $10^6$ cells). The decrease in BAL leukocyte total count was related to a significant inhibition on both neutrophil (72 ± 14 to 9 ± 3 × $10^6$ cells) and macrophage (36 ± 5 to 19 ± 3 × $10^6$ cells) infiltration. These results show that rapamycin is an effective inhibitor of leukocyte migration induced by LPS in guinea-pig airways. In addition, we have shown that neutrophil infiltration of guinea-pig airways induced by LPS is not associated with a significant increase in bronchial reactivity to either histamine or ACH.

### References

Morris, W. R. *Transplantation Reviews* 1992; **6**: 39–87.

*Present address: Department of Pharmacology, Federal University of Minas Gerais, Brazil.

### Oxidative activity of pulmonary phagocytes in acute pulmonary infection

*K. Dalhoff, J. Braun, M. Körber, H. Kothe, K. J. Wießann*
Dept. of Internal Medicine, Med. University, Ratzeburger Allee 160, D 2400 Lübeck, FRG.

The generation of reactive oxygen species (ROS) by pulmonary phagocytes is a critical step in non-specific host defence against pulmonary pathogens.We investigated the relative contribution of alveolar macrophages (AM) and neutrophils (PMN), which have different oxidant-forming capacity, to the production of ROS in BAL of 43 patients with acute pulmonary infection and ten controls. The chemiluminescence (CL) of $10^5$ phagocytes/0.5 ml was measured on a LB 953 luminometer (Berthold) continously during 30 min after enhancing with luminol (total Cl incl. halides) and lucigenin (mainly $O_2$); peak levels were taken for calculation. The neutrophil percentage in the BAL differential was markedly elevated in pneumonia (45 ± 34% vs 0.6 ± 0.4% in controls). The basal level of luminol-enhanced CL in patients 103 (controls) was 104.3 ± 123.7 (18.7 ± 9.7) cpm, $p<0.01$, and 1 678.7 ± 2 240.3 (169.7 ± 380.8) $10^3$ cpm, $p<0.01$ after stimulation with PMA. In contrast lucigenin-enhanced CL was not different between the two groups: 76.5 ± 101.3 (69.9 ± 55.1) $10^3$ cpm and 284.8 ± 334.5 (206.7 ± 183.8) $10^3$ cpm. Luminol-enhanced CL correlated strongly to the percentage of PMN in BAL ($r$=0.77, $p<0.001$). The increased oxidative activity in the alveolar compartment in acute pneumonia is mainly due to the influx of activated neutrophils, whereas the contribution of the resident AM to the production of ROS is only marginal.

### Effects of tobacco smokers' bronchoalveolar lavage fluid (sBALF) on neutrophil migration

*A. Verma, [1]T. J. Lockington, N. Walcot, T. D. Tetley, [1]M. C. Barrett and S. F. Smith*
Departments of Medicine & [1]Histopathology, Charing Cross & Westminster Medical School, London W6 8RF, LTK.

As polymorphonuclear neutrophil (PMN) numbers are increased in the lungs of tobacco smokers, we hypothesize that sBALF stimulates PMN recruitment into the lung. We studied the effects of sBALF from four patients on PMN chemotactic (CT) and undirected chemokinetic (CK) migration from heparinized venous blood. PMN were allowed to migrate under agarose from a central well for 4 h at 37°C towards graded doses of sBALF (CT stimulus) and 0.15 M NaCl (control stimulus). PMN were fixed and stained by conventional means. PMN numbers and linear distances moved towards control and CT stimuli were quantified in five fields by image analysis. sBALF had no effect on the distance moved by PMN, but at the highest dose ([total protein] = 625 ± 288 μg/ml, Mean ± SD) it increased the number of migrating PMN by 25% from 128 ± 11 to 163 ± 11 cells/field (mean ± SEM, $n$=4, $p<0.05$, Student's $t$-test). When sBALF was premixed with blood and PMN were then allowed to migrate under agarose towards a known CT stimulus, $10^{-6}$M $N$-formyl-met-leu-phe (FMLP) or towards a control to test for CK, sBALF at the lowest dose ([total protein] = 6 ± 3 μg/ml), inhibited the number of PMN migrating towards FMLP (CT) from 184 ± 19 (mean ± SEM; 216% of control) to 164 ± 29 cells/field (186% of control), but had a positive CK effect on control migration (+sBALF, 50% migrating PMN moved 250 μm; -sBALF, 50% migrating PMN moved 100 μm). Hence one or more components of sBALF have CT and CK properties which could increase PMN migration into the lung, but once there, the cells may be less able to respond to other CT stimuli such as bacteria.

### Inflammatory cells in bronchoalveolar lavage fluid in asthma and COPD

*J. Malolepszy, A. M. Fal and R. Dobek*
Dept of Internal Medicine and Allergology, Medical Academy of Wroclaw, ul. Traugutta 57/59, Wroclaw, Poland.

Bronchoalveolar lavage (BAL) fluid is a reliable tool to assess the inflammatory mechanisms underlying the pathophysiology of obstructive pulmonary diseases. The aim of our study was to evaluate the value of BAL fluid in differentiating COPD from asthma and to identify a link between cell counts and the severity of airflow obstruction. Thirty-five patients entered the study: ten atopic asthmatics (AA), ten non-atopic asthmatics (NA) and 15 subjects with COPD. The diagnosis was based on clinical history, physical examination, spirometry and specific IgE-level determinations (FAST). Each subject underwent bronchofiberoscopy with BAL fluid collection. The number of neutrophils was significantly higher in COPD as compared to AS and NA ($p$=0.01 and 0.05, respectively); on the contrary more eosinophils were found in AA ($p$=0.0016) and in NA ($p$=0.0013), as compared to COPD We did not find statistical differences in the percentage of lymphocytes in BAL fluid between COPD and the two groups of asthma patients, such a difference being observed between AA and NA ($p$=0.0248).

Our study confirms the predominant role of neutrophils in COPD and of eosinophils in asthma. Nevertheless, neutrophils seem to play an important role in the pathophysiology of non-atopic asthma. BAL fluid analysis may be of some value for the differential diagnosis of AA, NA and COPD, but it does not seem to be useful for the prognosis of these diseases.
